# Supplementary material for: β‐RA reduces DMQ/CoQ ratio and rescues the encephalopathic phenotype in Coq9 R239X mice
Source: EMBO Mol Med. 2018 Nov 27;11(1):e9466. doi: 10.15252/emmm.201809466 (PMC6328940; doi:10.15252/emmm.201809466)
Supplement: Supplementary file 1 — Appendix [file EMMM-11-e9466-s001.pdf]

β-RA reduces DMQ/CoQ ratio and recues the encephalopathic phenotype in *Coq9*^{R239X} mice

Agustín Hidalgo-Gutiérrez[1,2], Eliana Barriocanal-Casado[1,2], Mohammed Bakkali[4], M. Elena Díaz-Casado[1,2], Laura Sánchez-Maldonado[1,2], Miguel Romero[5], Ramy K. Sayed[2,6], Cornelia Prehn[7], Germaine Escames[1,2,3], Juan Duarte[5], Darío Acuña-Castroviejo[1,2,3], Luis C. López[1,2,3*]

APPENDIX

Table of contents

Appendix Figure S1, page 3

Appendix Figure S2, page 4

Appendix Figure S3, page 5

Appendix Figure S4, page 6

Appendix Figure S5, page 6

Appendix Figure S6, page 7

Appendix Figure S7, page 8

Appendix Figure S8, page 9

Appendix Figure S9, page 10

Appendix Figure S10, page 11

Appendix Figure S11, page 12

Appendix Figure S12, page 13

Appendix Table S1, page 14

Appendix Statistic Analysis, page 15

Supplemental Material and Methods, page 21

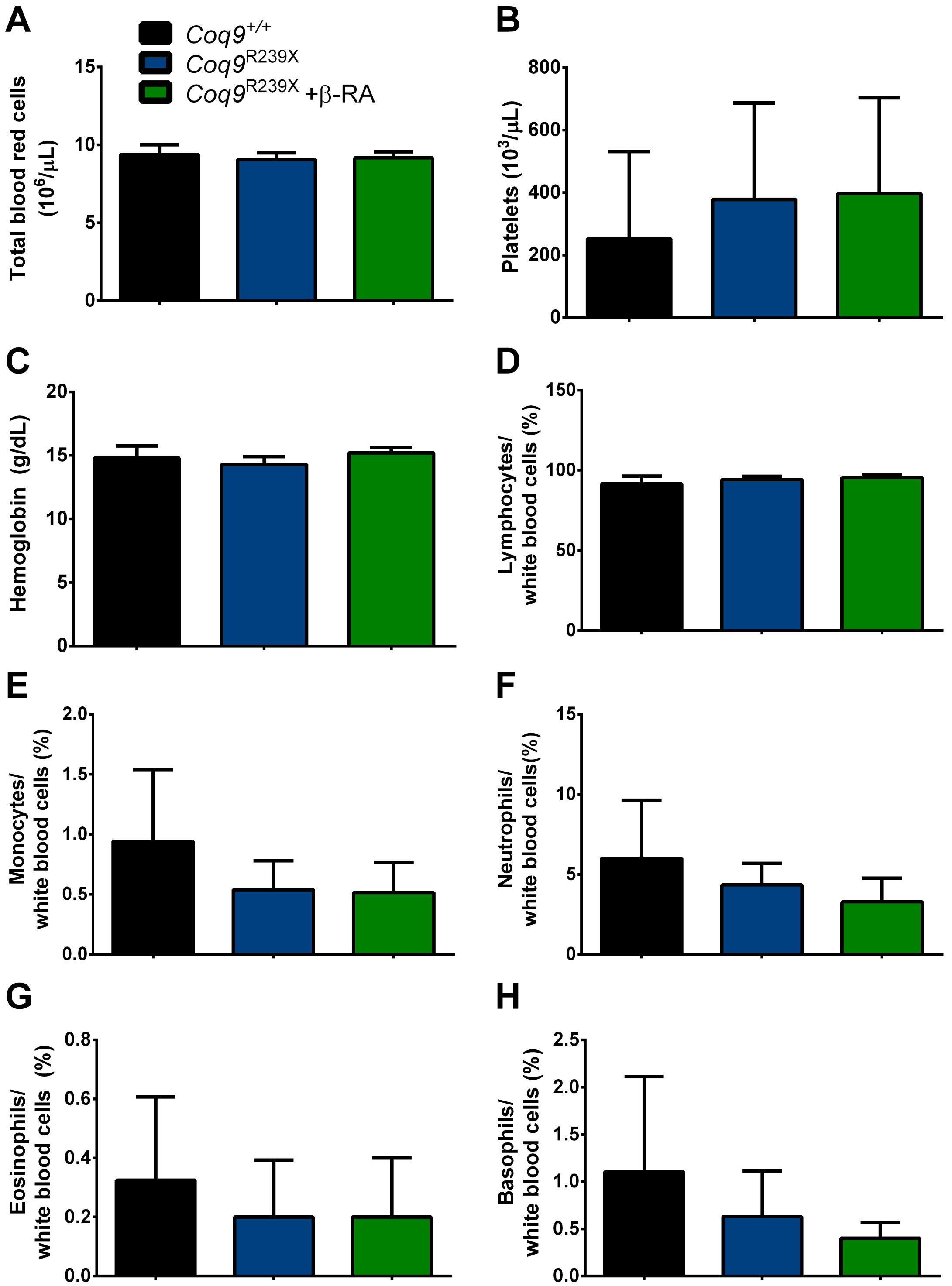

Figure S1. Hemogram after β-RA treatment. (**A**) Total blood cells, (**B**) number of platelets, (**C**) Hemoglobin levels, (**D**) percentage of lymphocytes, (**E**) percentage of monocytes, (**F**) percentage of neutrophils, (**G**) percentage of eosinophils and (**H**) percentage of basophils in *Coq9*$^{+/+}$ mice, *Coq9*^{R239X} mice and *Coq9*^{R239X} mice after β-RA treatment. Data

are expressed as mean ± SD. (one-way ANOVA with Tukey's post hoc test; n = 6–10 for each group). No changes were observed after the treatment.

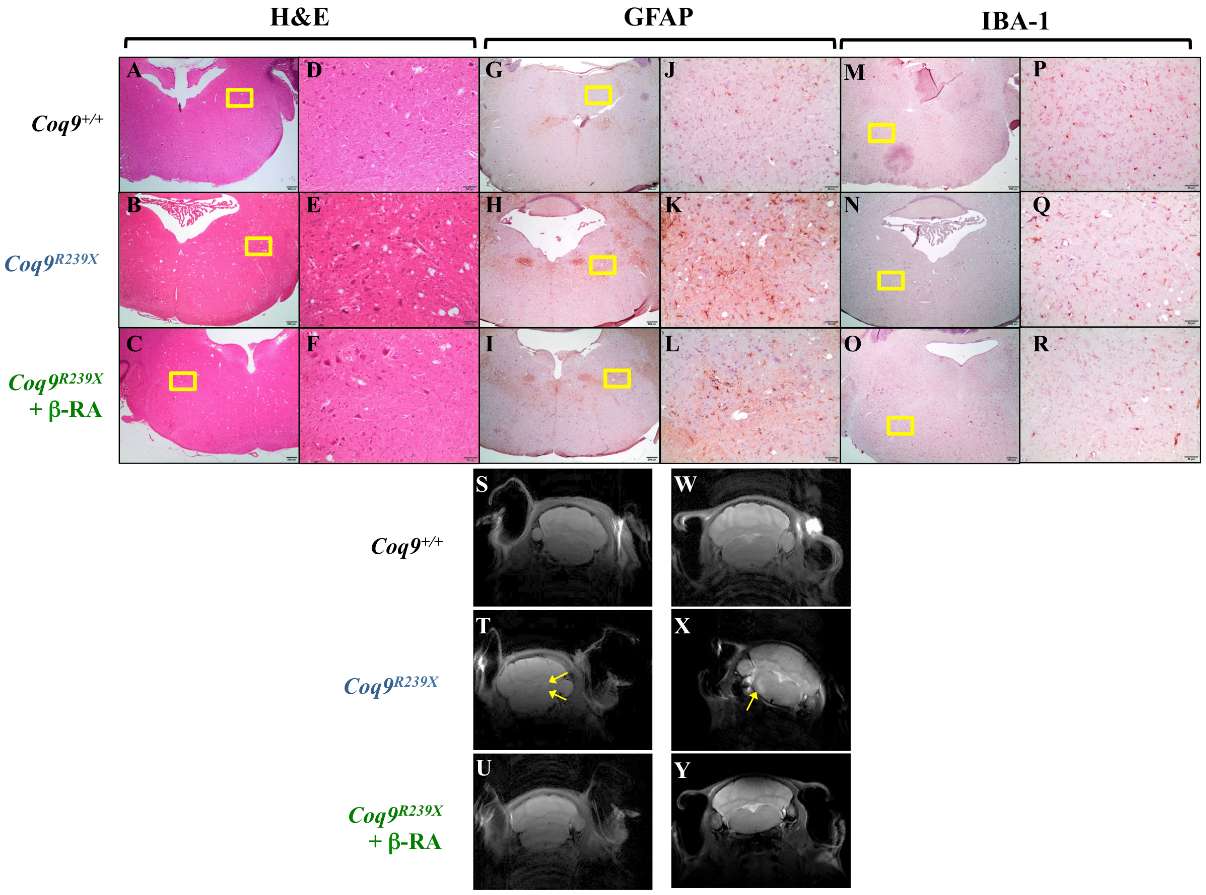

Fig. S2. Structural changes and distribution of astrocytes and microglia in the pons of *Coq9*^{R239X} mice after β-RA treatment.

(**A** to **F**) H&E stain in the pons of *Coq9*$^{+/+}$ mice (**A** and **D**), *Coq9*^{R239X} mice (**B** and **E**) and *Coq9*^{R239X} mice after β-RA treatment (**C** and **F**).

(**G** to **L**) Anti-GFAP stain in the pons of *Coq9*$^{+/+}$ mice (**G** and **J**), *Coq9*^{R239X} mice (**H** and **K**) and *Coq9*^{R239X} mice after β-RA treatment (**I** and **L**).

(**M** to **R**) Anti-Iba1 stain in the pons of *Coq9*$^{+/+}$ mice (**M** and **P**), *Coq9*^{R239X} mice (**N** and **Q**) and *Coq9*^{R239X} mice after β-RA treatment (**O** and **R**).

(**S** to **Y**) Magnetic Resonance Images of the diencephalon of *Coq9*$^{+/+}$ mice (**S and W**), *Coq9*^{R239X} mice (**T and X**) and *Coq9*^{R239X} mice after β-RA treatment (**U and Y**).

Scale bars: 200 µm (A-C); 50 µm (D-F); 200 µm (G-I); 50 µm (J-L); 200 µm (M-O); 50 µm (P-R).

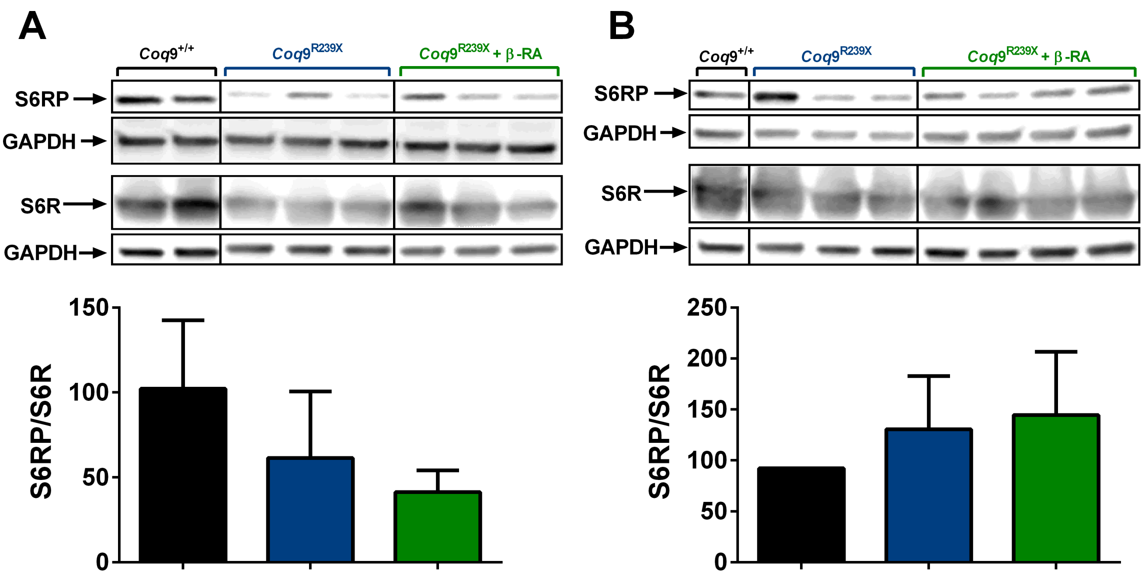

Figure S3. Assessment of the potential capacity of β-RA to inhibit mTORC1. (**A** and **B**) S6PR/S6R ratio in the brain (**A**) and the kidneys (**B**) of *Coq9*$^{+/+}$ mice, *Coq9*^{R239X} mice and *Coq9*^{R239X} mice treated with β-RA. (one-way ANOVA with Tukey's post hoc test; n = 4 for each group).

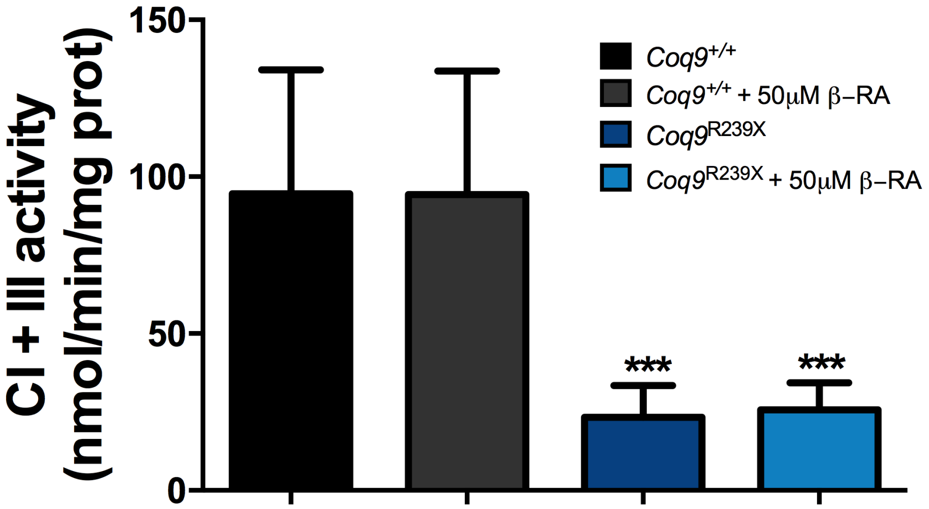

Figure S4. Effect of the addition of 50 µM β-RA into the reaction mix for measuring CI+III activity in isolated mitochondria from kidneys. (one-way ANOVA with Tukey's post hoc test; n = 5–6 for each group).

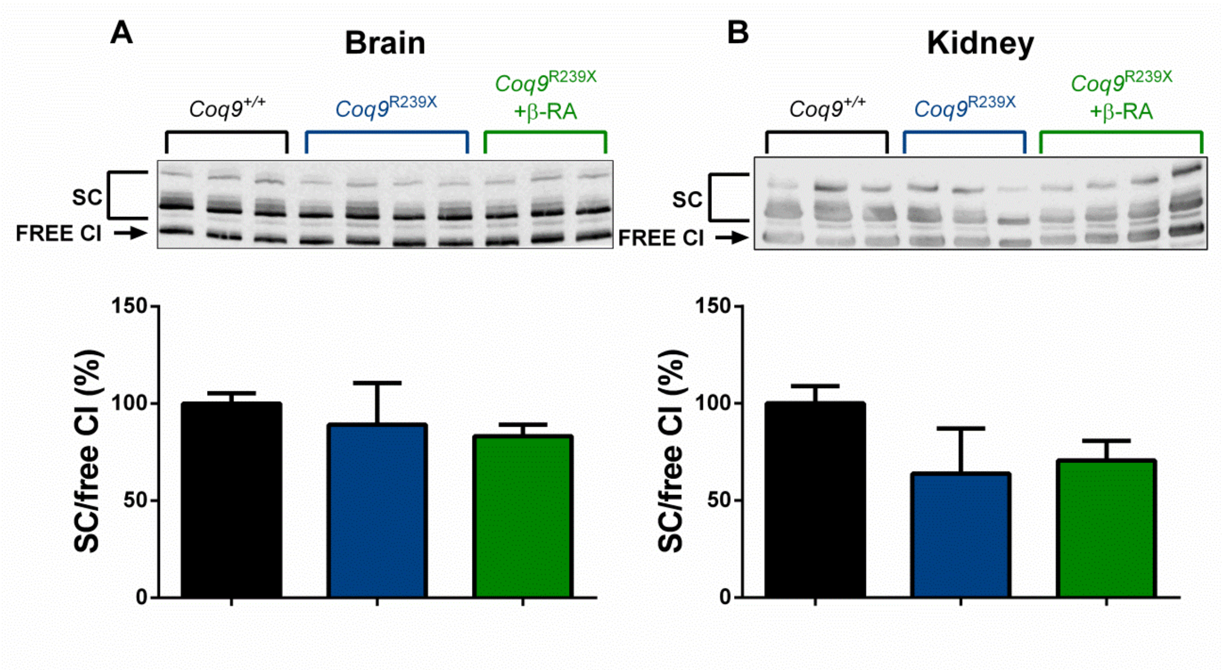

Figure S5. Mitochondrial complex I superassembly in the brain (**A**) and kidneys (**B**) of *Coq9*$^{+/+}$ mice, *Coq9*^{R239X} mice and *Coq9*^{R239X} mice after β-RA treatment. (one-way ANOVA with Tukey's post hoc test; n = 6 for each group).

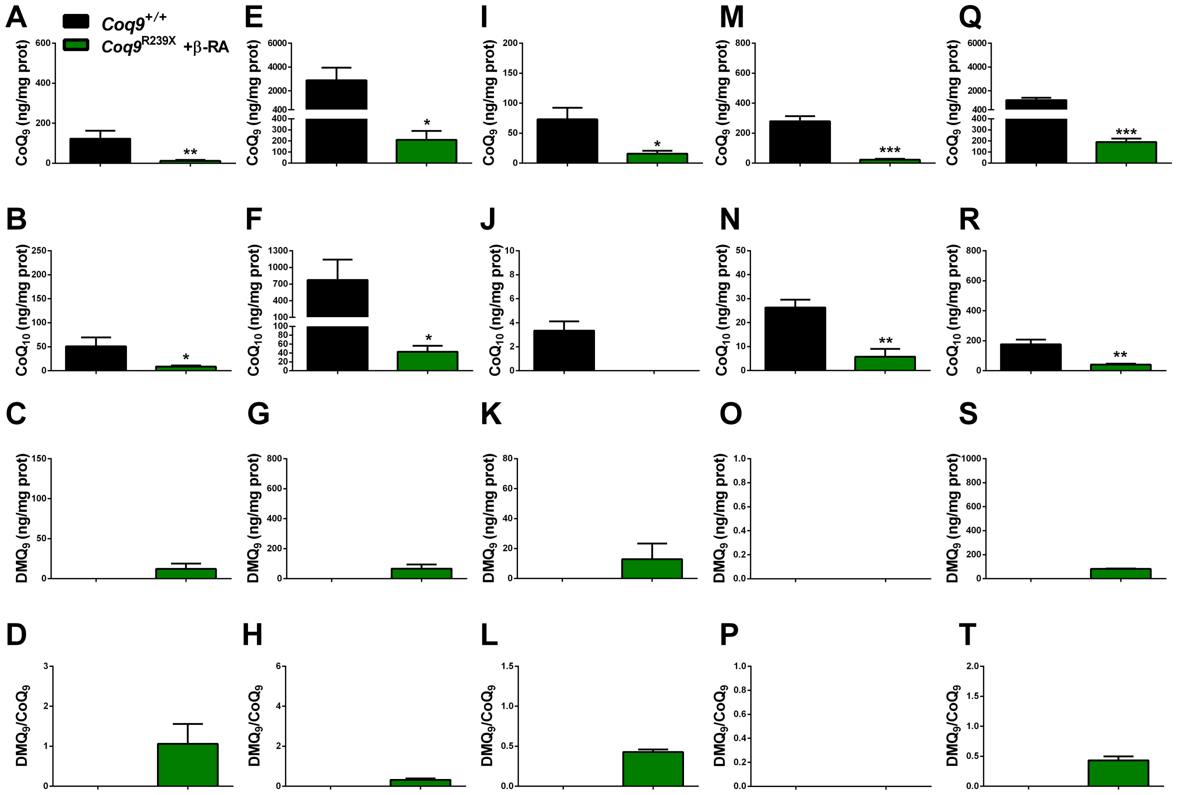

Figure S6 - Tissue-specific differences in the levels of CoQ_9, CoQ_{10} and DMQ_9, and in the DMQ_9/CoQ_9 ratio after 9 months of β-RA treatment in *Coq9*^{R239X} mice.

A-T Levels of CoQ_9 (A, E, I, M, Q), CoQ_{10} (B, F, J, N, R) and DMQ_9 (C, G, K, O, S), as well as and DMQ_9/CoQ_9 ratio (D, H, L, P, T) in brain (A-D), kidney (E-H), liver (I-L), skeletal muscle (M-P) and heart (Q-T) of *Coq9*$^{+/+}$ mice and *Coq9*^{R239X} mice after β-RA treatment. Note that data from *Coq9*^{R239X} mice are not shown since 100 % the animals are dead at 7 months of age. Data are expressed as mean ± SD. *P < 0.05; **P < 0.01; ***P < 0.001; (*t*-test; n = 4 for each group).

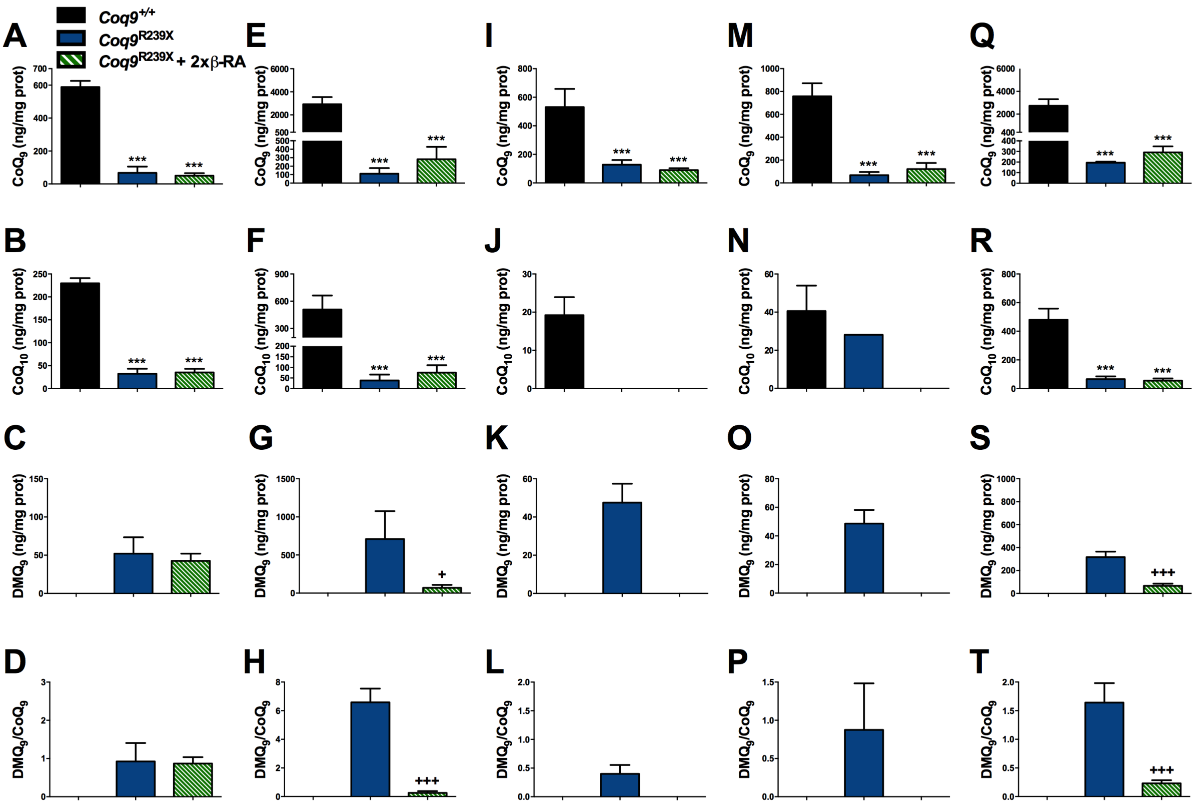

Figure S7 - Tissue-specific differences in the levels of CoQ_9, CoQ_{10} and DMQ_9, and in the DMQ_9/CoQ_9 ratio after 1 month of β-RA treatment at a dose of 2 g/kg b.w./day.

A-T Levels of CoQ_9 (A, E, I, M, Q), CoQ_{10} (B, F, J, N, R) and DMQ_9 (C, G, K, O, S), as well as and DMQ_9/CoQ_9 ratio (D, H, L, P, T) in brain (A-D), kidney (E-H), liver (I-L), skeletal muscle (M-P) and heart (Q-T) of *Coq9*$^{+/+}$ mice and *Coq9*^{R239X} mice after β-RA treatment. 2x = double dose. Data are expressed as mean ± SD. *P < 0.05; **P < 0.01; ***P < 0.001; *Coq9*^{R239X} or *Coq9*^{R239X} after β-RA treatment versus *Coq9*$^{+/+}$. +P < 0.05; ++P < 0.01; +P < 0.001; *Coq9*^{R239X} versus *Coq9*^{R239X} after β-RA treatment (one-way ANOVA with a Tukey's post hoc test; n = 5 for each group).

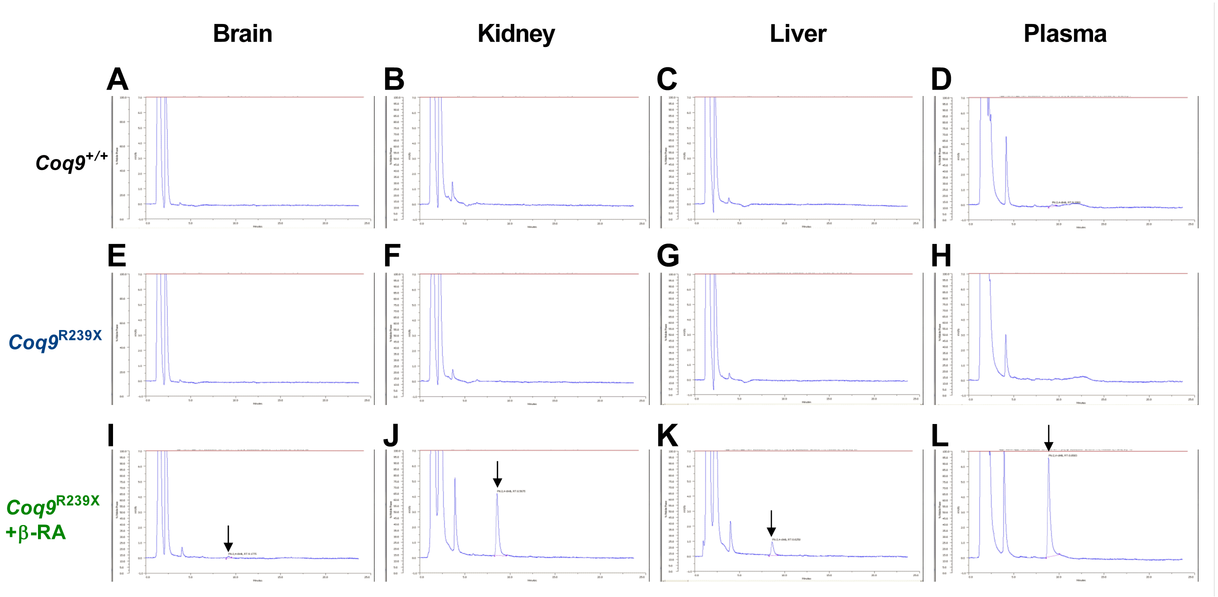

Figure S8. Representative chromatographs showing the peak of β-RA in mice tissues and plasma after the treatment. (**A** to **D**) Chromatographs for β-RA in tissues and plasma from *Coq9*$^{+/+}$ mice; (**E** to **H**) Chromatographs for β-RA in tissues and samples from *Coq9*^{R239X}, (**I** to **L**) Chromatographs for β-RA in tissues and samples from *Coq9*^{R239X} after β-RA treatment.

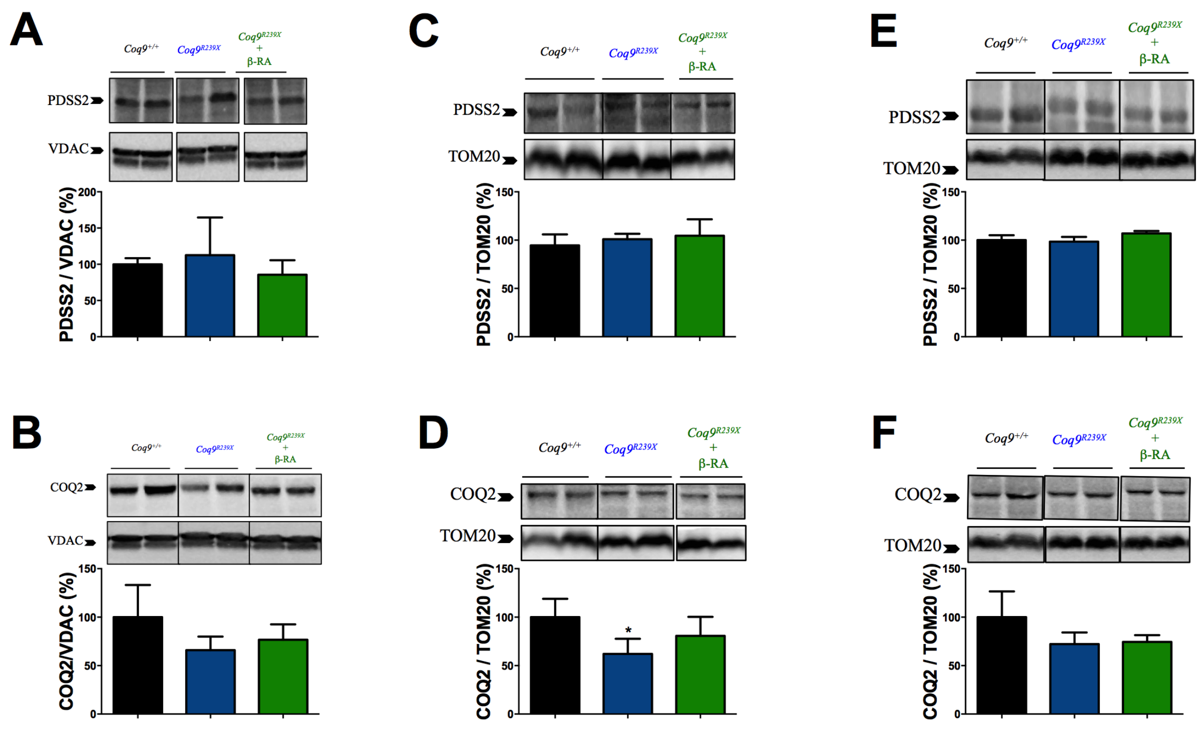

Figure S9. Effect of β-RA treatment in the tissue levels of CoQ biosynthetic proteins.

Representative images of western blots of the CoQ biosynthetic proteins PDSS2 and COQ2 and the quantitation of the protein bands in the brain (**A** and **B**), kidneys (**C** and **D**) and heart (**E** to **F**) of *Coq9*$^{+/+}$, *Coq9*^{R239X} *and Coq9*^{R239X} mice treated with β-RA.

Coq9$^{+/+}$ mice, *Coq9*^{R239X} mice and *Coq9*^{R239X} mice after β-RA treatment were included in all graphs. Data are expressed as mean ± SD. *P < 0.05; **P < 0.01; ***P < 0.001; *Coq9*^{R239X} or *Coq9*^{R239X} after β-RA treatment versus *Coq9*$^{+/+}$. +P < 0.05; *Coq9*^{R239X} versus and *Coq9*^{R239X} after β-RA treatment (one-way ANOVA with a Tukey's post hoc test; n = 4–6 for each group).

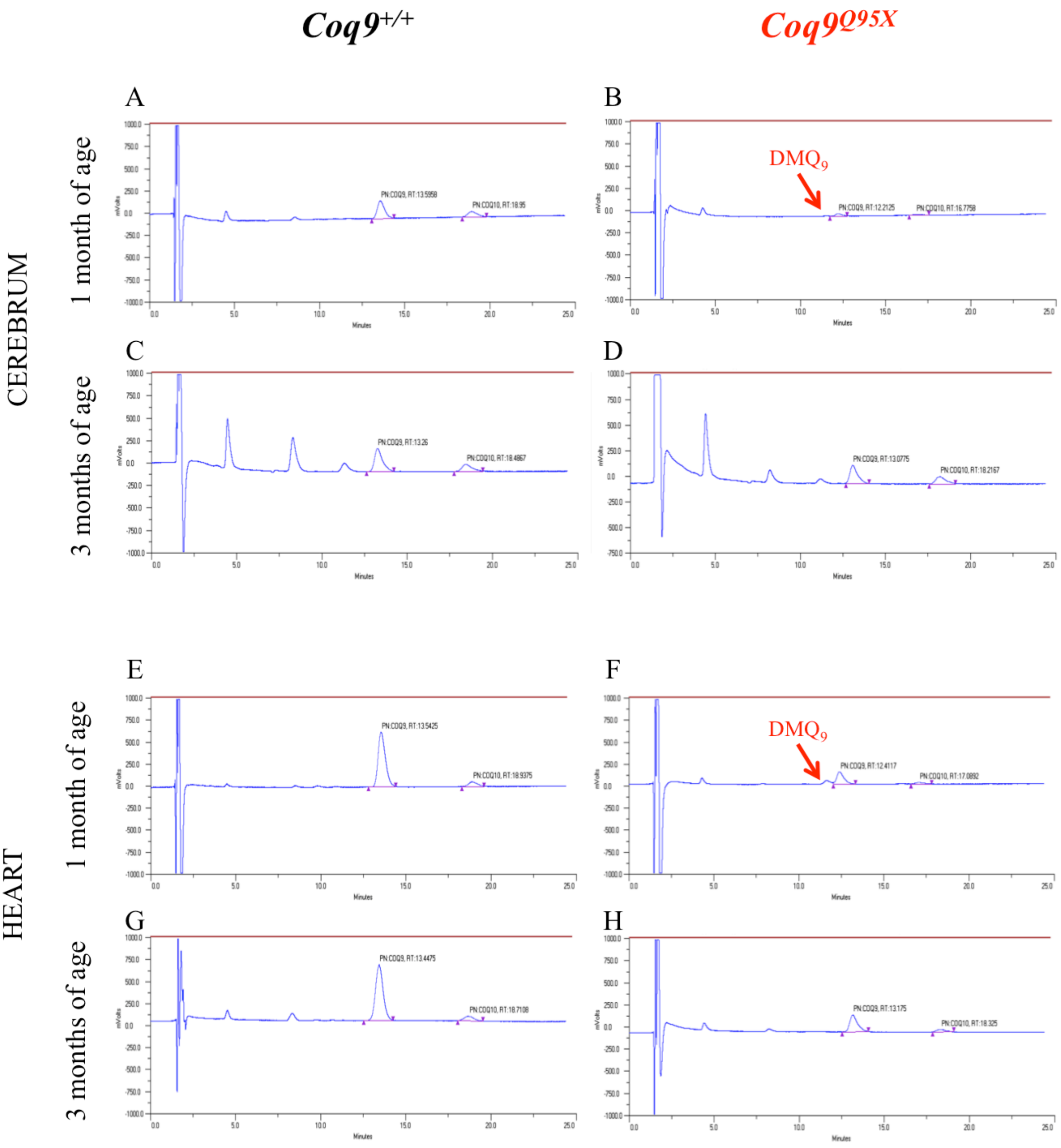

Figure S10. Representative chromatographs showing the peak of DMQ₉ in tissues from *Coq9*$^{+/+}$ and *Coq9*^{Q95X} mice at 1 and 3 months of age. (**A** to **D**) Chromatographs in the cerebrum; (**E** to **H**) Chromatographs in the heart. The DMQ_9 peak is detected in cerebrum and heart of *Coq9*^{Q95X} mice at 1 month of age (**B**, **F** and **J**).

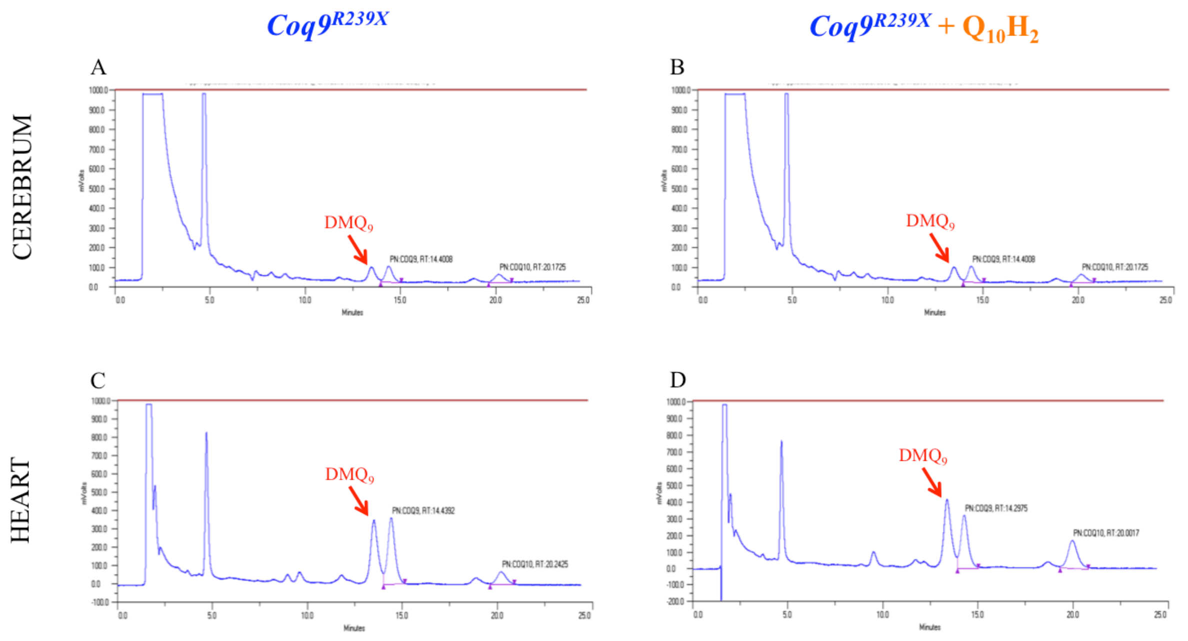

Figure S11. Representative chromatographs showing the peak of DMQ_9 in tissues from *Coq9*^{R239X} mice and *Coq9*^{R239X} mice treated with ubiquinol-10 ($Q_{10}H_2$) during two months. (**A** and **B**) Chromatographs in the cerebrum; (**C** and **D**) Chromatographs in the heart. The treatment with ubiquinol-10 did not modify the levels of DMQ_9.

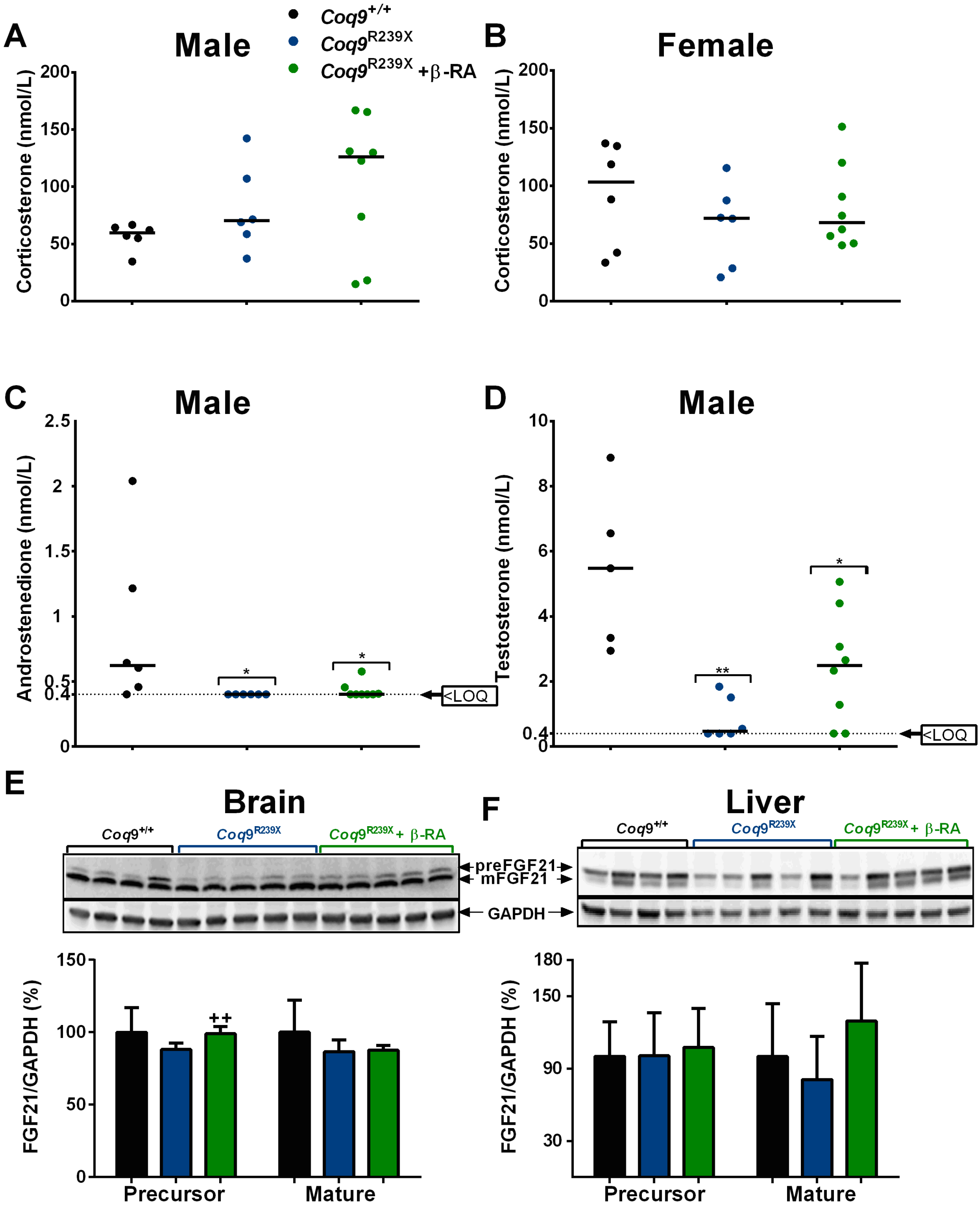

Figure S12. Levels of steroid hormones and FGF21. (**A** and **B**) Corticosterone levels in blood of male and female mice, respectively; (**C**) Androstenedione levels in blood of males mice; and (**D**) Testosterone levels in blood of males mice. (**E and F**) FGF21 levels in mice brains (**E**) and livers (**F**). *Coq9*$^{+/+}$ mice*,* *Coq9*^{R239X} mice and *Coq9*^{R239X} mice after β-RA

treatment were included in all graphs. Data are expressed as median. *P < 0.05; **P < 0.01; *Coq9*^{R239X} and *Coq9*^{R239X} after β-RA treatment mice versus *Coq9*$^{+/+}$ mice. ++P < 0.01; *Coq9*^{R239X} versus *Coq9*^{R239X} after β-RA treatment (Mann-Whitney test; n = 6-8 for each group).

Table S1. Levels of 4-HB in plasma and tissues of the mutant mice after oral or i.p. administration of β-RA.

Genotype	Administration Route	Time of sample collection	Number of samples with detected levels of 4-HB	Type of Samples	Levels of 4-HB (pg/mg prot)
Coq9$^{+/+}$	not treated	7:30-9:00	2/3	Brain	90.63 ± 22.89
Coq9^{R239X}	not treated	7:30-9:00	3/3	Brain	78.69 ± 2.35
Coq9^{R239X}	oral	7:30-9:00	6/6	Brain	82.35 ± 48.91
Coq9$^{+/+}$	not treated	7:30-9:00	3/3	Liver	61.08 ± 14.53
Coq9^{R239X}	not treated	7:30-9:00	2/3	Liver	39.16 ± 6.24
Coq9^{R239X}	oral	7:30-9:00	6/6	Liver	82.30 ± 35.73
Coq9$^{+/+}$	not treated	7:30-9:00	3/3	Kidney	144.72 ± 76.52
Coq9^{R239X}	not treated	7:30-9:00	3/3	Kidney	105.78 ± 40.93
Coq9^{R239X}	oral	7:30-9:00	6/6	Kidney	139.86 ± 37.5

APPENDIX STATISTIC ANALYSIS

Table S2. Statistical results for fig 1.

Figure 1A			
**Log-rank (Mantel-Cox) test**	**Significant?**	**Summary**	***p* value**
Coq9$^{+/+}$ vs. *Coq9*^{R239X}	Yes	***	<0.0001
Coq9$^{+/+}$ vs. *Coq9*^{R239X} + β-RA	Yes	**	0.0014
Coq9^{R239X} vs. *Coq9*^{R239X} + β-RA	Yes	***	<0.0001
Coq9$^{+/+}$ vs. *Coq9*^{R239X} + $Q_{10}H_2$	Yes	***	<0.0001
Coq9^{R239X} vs. *Coq9*^{R239X} + $Q_{10}H_2$	Yes	***	0.0006
Coq9^{R239X} + β-RA vs. *Coq9*^{R239X} + $Q_{10}H_2$	Yes	***	<0.0001
**Gehan-Breslow-Wilcoxon test**	**Significant?**	**Summary**	***p* value**
Coq9$^{+/+}$ vs. *Coq9*^{R239X}	Yes	***	<0.0001
Coq9$^{+/+}$ vs. *Coq9*^{R239X} + β-RA	Yes	**	0.0077
Coq9^{R239X} vs. *Coq9*^{R239X} + β-RA	Yes	***	<0.0001
Coq9$^{+/+}$ vs. *Coq9*^{R239X} + $Q_{10}H_2$	Yes	***	<0.0001
Coq9^{R239X} vs. *Coq9*^{R239X} + $Q_{10}H_2$	Yes	**	0.0024
Coq9^{R239X} + β-RA vs. *Coq9*^{R239X} + $Q_{10}H_2$	Yes	***	<0.0001
**Figure 1C**			
**Tukey's multiple comparisons test**	**Significant?**	**Summary**	***p* value**
2mo, *Coq9*$^{+/+}$ vs. *Coq9*^{R239X}	Yes	***	<0.0001
2mo, *Coq9*$^{+/+}$ vs. *Coq9*^{R239X} + β-RA	Yes	***	<0.0001
2mo, *Coq9*^{R239X} vs. *Coq9*^{R239X} + β-RA	No	ns	0.9095
3mo, *Coq9*$^{+/+}$ vs. *Coq9*^{R239X}	Yes	***	<0.0001
3mo, *Coq9*$^{+/+}$ vs. *Coq9*^{R239X} + β-RA	Yes	***	<0.0001
3mo, *Coq9*^{R239X} vs. *Coq9*^{R239X} + β-RA	Yes	+	0.0432
**t-test**	**Significant?**	**Summary**	***p* value**
7mo, *Coq9*$^{+/+}$ vs. *Coq9*^{R239X} + β-RA	Yes	***	0.0001
12mo, *Coq9*$^{+/+}$ vs. *Coq9*^{R239X} + β-RA	Yes	***	<0.0001
17mo, *Coq9*$^{+/+}$ vs. *Coq9*^{R239X} + β-RA	Yes	***	0.0001
22mo, *Coq9*$^{+/+}$ vs. *Coq9*^{R239X} + β-RA	Yes	***	0.0004
**Figure 1D**			
**Tukey's multiple comparisons test**	**Significant?**	**Summary**	***p* value**
2mo, *Coq9*$^{+/+}$ vs. *Coq9*^{R239X}	Yes	***	<0.0001
2mo, *Coq9*$^{+/+}$ vs. *Coq9*^{R239X} + β-RA	Yes	***	<0.0001
2mo, *Coq9*^{R239X} vs. *Coq9*^{R239X} + β-RA	No	ns	0.6012
3mo, *Coq9*$^{+/+}$ vs. *Coq9*^{R239X}	Yes	***	<0.0001
3mo, *Coq9*$^{+/+}$ vs. *Coq9*^{R239X} + β-RA	Yes	***	<0.0001
3mo, *Coq9*^{R239X} vs. *Coq9*^{R239X} + β-RA	No	ns	0.1062
**t-test**	**Significant?**	**Summary**	***p* value**
7mo, *Coq9*$^{+/+}$ vs. *Coq9*^{R239X} + β-RA	Yes	***	0.0007
12mo, *Coq9*$^{+/+}$ vs. *Coq9*^{R239X} + β-RA	Yes	**	0.0016
17mo, *Coq9*$^{+/+}$ vs. *Coq9*^{R239X} + β-RA	Yes	***	0.0001
22mo, *Coq9*$^{+/+}$ vs. *Coq9*^{R239X} + β-RA	Yes	*	0.0286
**Figure 1E**			
**Tukey's multiple comparisons test**	**Significant?**	**Summary**	***p* value**

3mo, $Coq9^{+/+}$ vs. $Coq9^{R239X}$	Yes	***	<0.0001
3mo, $Coq9^{+/+}$ vs. $Coq9^{R239X}$ + β-RA	Yes	**	0.0096
3mo, $Coq9^{R239X}$ vs. $Coq9^{R239X}$ + β-RA	No	ns	0.1657
**t-test**	**Significant?**	**Summary**	***p* value**
7mo, $Coq9^{+/+}$ vs. $Coq9^{R239X}$ + β-RA	No	ns	0.9685
**Figure 1F**			
**Tukey's multiple comparisons test**	**Significant?**	**Summary**	***p* value**
3mo, $Coq9^{+/+}$ vs. $Coq9^{R239X}$	Yes	**	0.0089
3mo, $Coq9^{+/+}$ vs. $Coq9^{R239X}$ + β-RA	No	ns	0.1689
3mo, $Coq9^{R239X}$ vs. $Coq9^{R239X}$ + β-RA	No	ns	0.1076
**t-test**	**Significant?**	**Summary**	***p* value**
7mo, $Coq9^{+/+}$ vs. $Coq9^{R239X}$ + β-RA	Yes	*	0.4895
**Figure 1 G**			
**Tukey's multiple comparisons test**	**Significant?**	**Summary**	***p* value**
$Coq9^{+/+}$ vs. $Coq9^{R239X}$	Yes	***	0.0008
$Coq9^{+/+}$ vs. $Coq9^{R239X}$ + β-RA	No	ns	0.7930
$Coq9^{R239X}$ vs. $Coq9^{R239X}$ + β-RA	Yes	++	0.0020

Table S3. Statistical results for fig 3.

**Figure 3D**			
**Tukey's multiple comparisons test**	**Significant?**	**Summary**	***p* value**
Control vs. LPS	Yes	***	<0.0001
Control vs. LPS + β-RA	Yes	***	0.0002
LPS vs. LPS + β-RA	No	ns	0.0664
**Figure 3E**			
**Tukey's multiple comparisons test**	**Significant?**	**Summary**	***p* value**
IL1-β, Control vs. LPS	No	ns	0.5822
IL1-β, Control vs. LPS + β-RA	No	ns	0.4158
IL1-β, LPS vs. LPS + β-RA	No	ns	0.9443
IL2, Control vs. LPS	Yes	*	0.0294
IL2, Control vs. LPS + β-RA	No	ns	0.1258
IL2, LPS vs. LPS + β-RA	No	ns	0.5093
IFN-γ, Control vs. LPS	No	ns	>0.9999
IFN-γ, Control vs. LPS + β-RA	No	ns	0.7455
IFN-γ, LPS vs. LPS + β-RA	No	ns	0.7455
Gro-α, Control vs. LPS	No	ns	0.1339
Gro-α, Control vs. LPS + β-RA	No	ns	0.3106
Gro-α, LPS vs. LPS + β-RA	No	ns	0.7869
TNF-α, Control vs. LPS	Yes	**	0.0069
TNF-α, Control vs. LPS + β-RA	Yes	***	0.0005
TNF-α, LPS vs. LPS + β-RA	No	ns	0.0505
**Figure 3F**			
**Tukey's multiple comparisons test**	**Significant?**	**Summary**	***p* value**
IL1-β, $Coq9^{+/+}$ vs. $Coq9^{R239X}$	No	ns	0.9759

IL1-β, $Coq9^{+/+}$ vs. $Coq9^{R239X}$ + β-RA	No	ns	0.1621
IL1-β, $Coq9^{R239X}$ vs. $Coq9^{R239X}$ + β-RA	No	ns	0.2293
IL2, $Coq9^{+/+}$ vs. $Coq9^{R239X}$	No	ns	0.9922
IL2, $Coq9^{+/+}$ vs. $Coq9^{R239X}$ + β-RA	No	ns	>0.9999
IL2, $Coq9^{R239X}$ vs. $Coq9^{R239X}$ + β-RA	No	ns	0.9390
IFN-γ, $Coq9^{+/+}$ vs. $Coq9^{R239X}$	No	ns	0.9754
IFN-γ, $Coq9^{+/+}$ vs. $Coq9^{R239X}$ + β-RA	No	ns	0.9899
IFN-γ, $Coq9^{R239X}$ vs. $Coq9^{R239X}$ + β-RA	No	ns	0.9999
Gro-α, $Coq9^{+/+}$ vs. $Coq9^{R239X}$	No	ns	0.9999
Gro-α, $Coq9^{+/+}$ vs. $Coq9^{R239X}$ + β-RA	No	ns	>0.9999
Gro-α, $Coq9^{R239X}$ vs. $Coq9^{R239X}$ + β-RA	No	ns	0.7869
TNF-α, $Coq9^{+/+}$ vs. $Coq9^{R239X}$	No	ns	0.9136
TNF-α, $Coq9^{+/+}$ vs. $Coq9^{R239X}$ + β-RA	No	ns	0.0612
TNF-α, $Coq9^{R239X}$ vs. $Coq9^{R239X}$ + β-RA	No	ns	0.1114
**Figure 3I**			
**Log-rank (Mantel-Cox) test**	**Significant?**	**Summary**	***p* value**
$Ndufs4^{-/-}$ vs. $Ndufs4^{-/-}$ + β-RA	No	ns	0.6209

Table S4. Statistical results for fig 4.

**Figure 4**			
**Tukey's multiple comparisons test**	**Significant?**	**Summary**	***p* value**
A, $Coq9^{+/+}$ vs. $Coq9^{R239X}$	No	**	0.0048
A, $Coq9^{+/+}$ vs. $Coq9^{R239X}$ + β-RA	Yes	***	0.0008
A, $Coq9^{R239X}$ vs. $Coq9^{R239X}$ + β-RA	No	ns	0.6859
B, $Coq9^{+/+}$ vs. $Coq9^{R239X}$	Yes	***	0.0008
B, $Coq9^{+/+}$ vs. $Coq9^{R239X}$ + β-RA	Yes	***	0.0001
B, $Coq9^{R239X}$ vs. $Coq9^{R239X}$ + β-RA	No	ns	0.6473
C, $Coq9^{+/+}$ vs. $Coq9^{R239X}$	Yes	***	<0.0001
C, $Coq9^{+/+}$ vs. $Coq9^{R239X}$ + β-RA	Yes	***	<0.0001
C, $Coq9^{R239X}$ vs. $Coq9^{R239X}$ + β-RA	No	ns	0.8374
D, $Coq9^{+/+}$ vs. $Coq9^{R239X}$	Yes	**	0.0027
D, $Coq9^{+/+}$ vs. $Coq9^{R239X}$ + β-RA	Yes	***	0.0005
D, $Coq9^{R239X}$ vs. $Coq9^{R239X}$ + β-RA	No	ns	0.8549
F, $Coq9^{+/+}$ vs. $Coq9^{R239X}$	Yes	*	0.0478
F, $Coq9^{+/+}$ vs. $Coq9^{R239X}$ + β-RA	No	ns	0.8840
F, $Coq9^{R239X}$ vs. $Coq9^{R239X}$ + β-RA	Yes	+	0.0195
G, $Coq9^{+/+}$ vs. $Coq9^{R239X}$	No	ns	0.1521
G, $Coq9^{+/+}$ vs. $Coq9^{R239X}$ + β-RA	No	ns	0.9794
G, $Coq9^{R239X}$ vs. $Coq9^{R239X}$ + β-RA	No	ns	0.0909
H, $Coq9^{+/+}$ vs. $Coq9^{R239X}$	Yes	**	0.0034
H, $Coq9^{+/+}$ vs. $Coq9^{R239X}$ + β-RA	Yes	*	0.0113
H, $Coq9^{R239X}$ vs. $Coq9^{R239X}$ + β-RA	No	ns	0.6915
I, $Coq9^{+/+}$ vs. $Coq9^{R239X}$	No	ns	0.0754
I, $Coq9^{+/+}$ vs. $Coq9^{R239X}$ + β-RA	No	ns	0.2106

I, *Coq9*^{R239X} vs. *Coq9*^{R239X} + β-RA	No	ns	0.7579
K, *Coq9*$^{+/+}$ vs. *Coq9*^{R239X}	Yes	*	0.0121
K, *Coq9*$^{+/+}$ vs. *Coq9*^{R239X} + β-RA	No	ns	0.2272
K, *Coq9*^{R239X} vs. *Coq9*^{R239X} + β-RA	No	ns	0.2767
L, *Coq9*$^{+/+}$ vs. *Coq9*^{R239X}	No	ns	0.2464
L, *Coq9*$^{+/+}$ vs. *Coq9*^{R239X} + β-RA	No	ns	0.6080
L, *Coq9*^{R239X} vs. *Coq9*^{R239X} + β-RA	No	ns	0.7050
M, *Coq9*$^{+/+}$ vs. *Coq9*^{R239X}	Yes	**	0.0023
M, *Coq9*$^{+/+}$ vs. *Coq9*^{R239X} + β-RA	Yes	*	0.0176
M, *Coq9*^{R239X} vs. *Coq9*^{R239X} + β-RA	No	ns	0.5302
N, *Coq9*$^{+/+}$ vs. *Coq9*^{R239X}	Yes	*	0.0182
N, *Coq9*$^{+/+}$ vs. *Coq9*^{R239X} + β-RA	No	ns	0.1242
N, *Coq9*^{R239X} vs. *Coq9*^{R239X} + β-RA	No	ns	0.5745
O, *Coq9*$^{+/+}$ vs. *Coq9*^{R239X}	Yes	*	0.0253
O, *Coq9*$^{+/+}$ vs. *Coq9*^{R239X} + β-RA	No	ns	0.0762
O, *Coq9*^{R239X} vs. *Coq9*^{R239X} + β-RA	No	ns	0.6296

Table S5. Statistical results for fig 5.

Figure 5			
**Tukey's multiple comparisons test**	**Significant?**	**Summary**	***p* value**
A, *Coq9*$^{+/+}$ vs. *Coq9*^{R239X}	Yes	**	0.0010
A, *Coq9*$^{+/+}$ vs. *Coq9*^{R239X} + β-RA	Yes	***	0.0004
A, *Coq9*^{R239X} vs. *Coq9*^{R239X} + β-RA	No	ns	0.9454
B, *Coq9*$^{+/+}$ vs. *Coq9*^{R239X}	Yes	**	0.0032
B, *Coq9*$^{+/+}$ vs. *Coq9*^{R239X} + β-RA	Yes	***	0.0009
B, *Coq9*^{R239X} vs. *Coq9*^{R239X} + β-RA	No	ns	0.8785
E, *Coq9*$^{+/+}$ vs. *Coq9*^{R239X}	Yes	***	<0.0001
E, *Coq9*$^{+/+}$ vs. *Coq9*^{R239X} + β-RA	Yes	***	<0.0001
E, *Coq9*^{R239X} vs. *Coq9*^{R239X} + β-RA	No	ns	0.6727
F, *Coq9*$^{+/+}$ vs. *Coq9*^{R239X}	Yes	***	<0.0001
F, *Coq9*$^{+/+}$ vs. *Coq9*^{R239X} + β-RA	Yes	***	<0.0001
F, *Coq9*^{R239X} vs. *Coq9*^{R239X} + β-RA	No	ns	0.8952
I, *Coq9*$^{+/+}$ vs. *Coq9*^{R239X}	Yes	***	0.0003
I, *Coq9*$^{+/+}$ vs. *Coq9*^{R239X} + β-RA	Yes	***	<0.0001
I, *Coq9*^{R239X} vs. *Coq9*^{R239X} + β-RA	No	ns	0.5556
J, *Coq9*$^{+/+}$ vs. *Coq9*^{R239X}	Yes	#	#
J, *Coq9*$^{+/+}$ vs. *Coq9*^{R239X} + β-RA	Yes	#	#
J, *Coq9*^{R239X} vs. *Coq9*^{R239X} + β-RA	No	#	#
M, *Coq9*$^{+/+}$ vs. *Coq9*^{R239X}	Yes	***	<0.0001
M, *Coq9*$^{+/+}$ vs. *Coq9*^{R239X} + β-RA	Yes	***	<0.0001
M, *Coq9*^{R239X} vs. *Coq9*^{R239X} + β-RA	No	ns	0.9059
N, *Coq9*$^{+/+}$ vs. *Coq9*^{R239X}	Yes	***	<0.0001
N, *Coq9*$^{+/+}$ vs. *Coq9*^{R239X} + β-RA	Yes	***	<0.0001
N, *Coq9*^{R239X} vs. *Coq9*^{R239X} + β-RA	No	ns	0.4343

Q, *Coq9*$^{+/+}$ vs. *Coq9*^{R239X}	Yes	**	0.0002
Q, *Coq9*$^{+/+}$ vs. *Coq9*^{R239X} + β-RA	Yes	*	<0.0001
Q, *Coq9*^{R239X} vs. *Coq9*^{R239X} + β-RA	No	ns	0.9992
R, *Coq9*$^{+/+}$ vs. *Coq9*^{R239X}	Yes	**	0.0016
R, *Coq9*$^{+/+}$ vs. *Coq9*^{R239X} + β-RA	Yes	***	0.0004
R, *Coq9*^{R239X} vs. *Coq9*^{R239X} + β-RA	No	ns	0.9991
**Figure 5**			
**t-test**	**Significant?**	**Summary**	***p* value**
C, *Coq9*^{R239X} vs. *Coq9*^{R239X} + β-RA	No	ns	0.8050
D, *Coq9*^{R239X} vs. *Coq9*^{R239X} + β-RA	No	ns	0.0590
G, *Coq9*^{R239X} vs. *Coq9*^{R239X} + β-RA	Yes	++	0.0014
H, *Coq9*^{R239X} vs. *Coq9*^{R239X} + β-RA	Yes	++	0.0034
K, *Coq9*^{R239X} vs. *Coq9*^{R239X} + β-RA	Yes	+	0.0173
L, *Coq9*^{R239X} vs. *Coq9*^{R239X} + β-RA	No	ns	0.1089
O, *Coq9*^{R239X} vs. *Coq9*^{R239X} + β-RA	#	#	0.7186
P, *Coq9*^{R239X} vs. *Coq9*^{R239X} + β-RA	#	#	0.0761
S, *Coq9*^{R239X} vs. *Coq9*^{R239X} + β-RA	Yes	++	0.0018
T, *Coq9*^{R239X} vs. *Coq9*^{R239X} + β-RA	Yes	++	0.0069

Table S6. Statistical results for fig 6.

**Figure 6**			
**Tukey's multiple comparisons test**	**Significant?**	**Summary**	***p* value**
A-Brain COQ4, *Coq9*$^{+/+}$ vs. *Coq9*^{R239X}	Yes	***	<0.0001
A-Brain COQ4, *Coq9*$^{+/+}$ vs. *Coq9*^{R239X} + β-RA	Yes	***	<0.0001
A-Brain COQ4, *Coq9*^{R239X} vs. *Coq9*^{R239X} + β-RA	No	ns	0.4267
B-Brain COQ5, *Coq9*$^{+/+}$ vs. *Coq9*^{R239X}	Yes	***	<0.0001
B-Brain COQ5, *Coq9*$^{+/+}$ vs. *Coq9*^{R239X} + β-RA	Yes	***	<0.0001
B-Brain COQ5, *Coq9*^{R239X} vs. *Coq9*^{R239X} + β-RA	No	ns	0.5206
C-Brain COQ6, *Coq9*$^{+/+}$ vs. *Coq9*^{R239X}	Yes	**	0.0025
C-Brain COQ6, *Coq9*$^{+/+}$ vs. *Coq9*^{R239X} + β-RA	Yes	**	0.0055
C-Brain COQ6, *Coq9*^{R239X} vs. *Coq9*^{R239X} + β-RA	No	ns	0.8672
D-Brain COQ7, *Coq9*$^{+/+}$ vs. *Coq9*^{R239X}	#	#	#
D-Brain COQ7, *Coq9*$^{+/+}$ vs. *Coq9*^{R239X} + β-RA	#	#	#
D-Brain COQ7, *Coq9*^{R239X} vs. *Coq9*^{R239X} + β-RA	#	#	#
E-Brain COQ8A, *Coq9*$^{+/+}$ vs. *Coq9*^{R239X}	Yes	**	0.0028
E-Brain COQ8A, *Coq9*$^{+/+}$ vs. *Coq9*^{R239X} + β-RA	Yes	**	0.0014
E-Brain COQ8A, *Coq9*^{R239X} vs. *Coq9*^{R239X} + β-RA	No	ns	0.8641
F-Kidney COQ4, *Coq9*$^{+/+}$ vs. *Coq9*^{R239X}	Yes	***	<0.0001
F-Kidney COQ4, *Coq9*$^{+/+}$ vs. *Coq9*^{R239X} + β-RA	Yes	***	<0.0001
F-Kidney COQ4, *Coq9*^{R239X} vs. *Coq9*^{R239X} + β-RA	No	ns	0.6088
G-Kidney COQ5, *Coq9*$^{+/+}$ vs. *Coq9*^{R239X}	Yes	***	<0.0001

G-Kidney COQ5, *Coq9*$^{+/+}$ vs. *Coq9*^{R239X} + β-RA	Yes	***	<0.0001
G-Kidney COQ5, *Coq9*^{R239X} vs. *Coq9*^{R239X} + β-RA	No	ns	0.3238
H-Kidney COQ6, *Coq9*$^{+/+}$ vs. *Coq9*^{R239X}	Yes	**	0.0014
H-Kidney COQ6, *Coq9*$^{+/+}$ vs. *Coq9*^{R239X} + β-RA	Yes	*	0.0338
H-Kidney COQ6, *Coq9*^{R239X} vs. *Coq9*^{R239X} + β-RA	No	ns	0.1542
I-Kidney COQ7, *Coq9*$^{+/+}$ vs. *Coq9*^{R239X}	Yes	**	0.0026
I-Kidney COQ7, *Coq9*$^{+/+}$ vs. *Coq9*^{R239X} + β-RA	Yes	**	0.0015
I-Kidney COQ7, *Coq9*^{R239X} vs. *Coq9*^{R239X} + β-RA	No	ns	0.9980
J-Kidney COQ8A, *Coq9*$^{+/+}$ vs. *Coq9*^{R239X}	Yes	**	0.0075
J-Kidney COQ8A, *Coq9*$^{+/+}$ vs. *Coq9*^{R239X} + β-RA	Yes	ns	0.5417
J-Kidney COQ8A, *Coq9*^{R239X} vs. *Coq9*^{R239X} + β-RA	No	+	0.0365
K-Heart COQ4, *Coq9*$^{+/+}$ vs. *Coq9*^{R239X}	Yes	***	<0.0001
K-Heart COQ4, *Coq9*$^{+/+}$ vs. *Coq9*^{R239X} + β-RA	Yes	***	<0.0001
K-Heart COQ4, *Coq9*^{R239X} vs. *Coq9*^{R239X} + β-RA	No	ns	0.6598
L-Heart COQ5, *Coq9*$^{+/+}$ vs. *Coq9*^{R239X}	Yes	***	<0.0001
L-Heart COQ5, *Coq9*$^{+/+}$ vs. *Coq9*^{R239X} + β-RA	Yes	***	<0.0001
L-Heart COQ5, *Coq9*^{R239X} vs. *Coq9*^{R239X} + β-RA	No	ns	0.0761
M-Heart COQ6, *Coq9*$^{+/+}$ vs. *Coq9*^{R239X}	Yes	**	0.0010
M-Heart COQ6, *Coq9*$^{+/+}$ vs. *Coq9*^{R239X} + β-RA	Yes	*	0.0110
M-Heart COQ6, *Coq9*^{R239X} vs. *Coq9*^{R239X} + β-RA	No	ns	0.1376
O-Heart COQ7, *Coq9*$^{+/+}$ vs. *Coq9*^{R239X}	Yes	***	<0.0001
O-Heart COQ7, *Coq9*$^{+/+}$ vs. *Coq9*^{R239X} + β-RA	Yes	***	<0.0001
O-Heart COQ7, *Coq9*^{R239X} vs. *Coq9*^{R239X} + β-RA	No	ns	0.9059
P-Heart COQ8A, *Coq9*$^{+/+}$ vs. *Coq9*^{R239X}	Yes	***	0.0004
P-Heart COQ8A, *Coq9*$^{+/+}$ vs. *Coq9*^{R239X} + β-RA	Yes	***	0.0006
P-Heart COQ8A, *Coq9*^{R239X} vs. *Coq9*^{R239X} + β-RA	No	ns	0.7186

# COQ7 protein in the brain of Coq9^{R239X} *mice (Fig 7D) is undetectable, so the statistic analysis is not possible in that case.*

Table S7. Statistical results for fig 7.

**Figure 7A**			
**t-test**	**Significant?**	**Summary**	***p* value**
Brain, *Coq9*^{R239X} vs. *Coq9*^{Q95X}	Yes	***	<0.0001

Kidney, $Coq9^{R239X}$ vs. $Coq9^{Q95X}$	Yes	***	<0.0001
Liver, $Coq9^{R239X}$ vs. $Coq9^{Q95X}$	Yes	**	0.0088
Muscle, $Coq9^{R239X}$ vs. $Coq9^{Q95X}$	Yes	***	<0.0001
Heart, $Coq9^{R239X}$ vs. $Coq9^{Q95X}$	Yes	***	0.0003
**Figure 7C**			
**t-test**	**Significant?**	**Summary**	***p* value**
Brain, $Coq9^{R239X}$ vs. $Coq9^{Q95X}$	Yes	**	0.0021
Kidney, $Coq9^{R239X}$ vs. $Coq9^{Q95X}$	Yes	***	<0.0001
Liver, $Coq9^{R239X}$ vs. $Coq9^{Q95X}$	Yes	***	0.0003
Muscle, $Coq9^{R239X}$ vs. $Coq9^{Q95X}$	Yes	**	0.0011
Heart, $Coq9^{R239X}$ vs. $Coq9^{Q95X}$	Yes	***	0.0001
**Figure 7E**			
**t-test**	**Significant?**	**Summary**	***p* value**
Brain, $Coq9^{R239X}$ vs. $Coq9^{R239X}$ + QH_2	No	ns	0.9254
Kidney, $Coq9^{R239X}$ vs. $Coq9^{R239X}$ + QH_2	No	ns	0.8124
Liver, $Coq9^{R239X}$ vs. $Coq9^{R239X}$ + QH_2	No	ns	0.1062
Muscle, $Coq9^{R239X}$ vs. $Coq9^{R239X}$ + QH_2	No	ns	0.7364
Heart, $Coq9^{R239X}$ vs. $Coq9^{R239X}$ + QH_2	No	ns	0.4820

SUPPLEMETAL MATERIAL AND METHODS

Experimental design

$Coq9^{+/+}$, $Coq9^{R239X}$, $Ndufs4^{+/+}$ and $Ndufs4^{-/-}$ mice were used in the study. All mice have a mix of C57BL/6N and C57BL/6J genetic background. Mice were housed in the Animal Facility of the University of Granada under an SPF zone with lights on at 7:00 AM and off at 7:00 PM. Mice had unlimited access to water and rodent chow. The $Coq9^{R239X}$ mouse model (http://www.informatics.jax.org/allele/key/8292711) was previously generated and characterized (Garcia-Corzo et al, 2013). Only $Coq9^{+/+}$ mice, $Coq9^{R239X}$ mice and $Coq9^{R239X}$ mice treated with β-RA were used in the study. Also, a pilot survival study was done with β-RA in *Ndufs4* knockout mice, which show microgliosis and neuroinflammation (Quintana et al, 2010).

Mice were housed in the Animal Facility of the University of Granada under a SPF zone with lights on at 7:00 AM and off at 7:00 PM. Mice had unlimited access to water and rodent

chow. Animals were randomly assigned in experimental groups. Data were randomly collected and processed as well. All experiments were performed according to a protocol approved by the Institutional Animal Care and Use Committee of the University of Granada (procedures Granada, Spain. n°: 9-CEEA-OH-2013) and in accordance with the European Convention for the Protection of Vertebrate Animals used for Experimental and Other Scientific Purposes (CETS # 123), directive 2010/63/EU on the protection of animals used for scientific purposes and the Spanish law (R.D. 53/2013).

β-RA was incorporated in the chow at a concentration of 1 %. Therefore, the treatment consisted of administering β-RA in the chow at a starting dose of 3 g/kg bw/day, which was decreasing until 1 g/kg bw/day with the increase of the animal body weight. The treatment started at 1 month of age, and the mice were sacrificed for the experimental assays at 3 months of age. For the survival curve, we performed an analysis starting the treatment at 1 month of age (before the start of the severe disease symptoms) and a second analysis where the treatment started at 3 months of age (when the severe symptoms start). In the *Ndufs4* knockout mice, the treatment started at 21 days old.

The body weights of the animals were collected at 2, 3, 7, 12, 17 and 22 months of age. The motor coordination was evaluated by rotarod test at different months of age. Systolic blood pressure (SBP) and heart rate (HR) were measured in conscious, prewarmed, and restrained mice by tail-cuff plethysmography (Digital Pressure Meter LE 5001, Letica S.A., Barcelona, Spain) as described previously (Gomez-Guzman et al, 2014; Luna-Sanchez et al, 2017).

The therapeutic potential of β-RA was also assessed in zebrafish embryos treated with the neurotoxin 1-methyl-4-phenyl- 1,2,3,6-tetrahydropyridine (MPTP), which is used to induce key features of Parkinson Disease, including CI inhibition and neuroinflammation (Diaz-Casado et al, 2018). Zebrafish embryos at 24 hpf (hours postfertilization) were dechorionated manually and were randomly distributed in 24-well plate (six embryos per well) containing

E3 medium with or without the drugs in a total volume of 1 mL. Stock solution of MPTP (Sigma-Aldrich, Madrid, Spain) was freshly prepared in E3 medium and administered at a concentration of 600 µM. β-RA was dissolved in E3 medium and administered at a concentration of 1 or 10 mM . The embryos were exposed to MPTP from 24 hpf to 72 hpf, and β-RA was added to the wells from 72 hpf to 120 hpf. Half of the solution of each group was replaced daily with a fresh prewarmed solution. After completing the experiment protocol, at 120 hpf, the mortality rate was calculated as the percentage of dead embryos with respect to total embryos. Oedema, the most common malformations, as well as tail and yolk abnormalities were macroscopically quantified (Diaz-Casado et al, 2018). Adult zebrafish (*Danio rerio*) of the AB line were provided by ZFBiolabs S.L (Madrid, Spain) and used as breeding stocks. The fish line was maintained in the University of Granada's facility at a water temperature of 28.5 1°C and under a photoperiod of 14:10 hr (lights on at 08:00 hr) in a recirculation aquaculture system (Aquaneering Incorporated, Barcelona, Spain).

Experiments in mice were performed according to a protocol approved by the Institutional Animal Care and Use Committee of the University of Granada (procedures 9-CEEA-OH-2013) and were in accordance with the European Convention for the Protection of Vertebrate Animals used for Experimental and Other Scientific Purposes (CETS # 123) and the Spanish law (R.D. 53/2013). Experiments in zebrafish were performed according to a protocol approved by the Institutional Animal Care and Use Committee of the University of Granada (procedures CEEA 2010-275).

Histology and Immunohistochemistry

Mice tissues were formalin-fixed and paraffin-embedded. Multiple sections (4 µm thickness) were deparaffinized with xylene and stained with hematoxylin and eosin (H&E).

Immunohistochemistry was carried out in the same sections, using the following primary antibodies: anti-GFAP (Millipore, MAB360, dilution 1:400) and anti-Iba-1 (Wako, 019-19741, dilution 1:500). The detection of carbonyl groups as a marker of protein oxidation was performed using a commercial kit (OxyIHC™ Oxidative Stress Detection Kit (S7450, Millipore) (Garcia-Corzo et al, 2013). Dako Animal Research Kit for mouse primary antibodies (Dako Diagnóstico S.A., Spain) was used for the qualitative identification of antigens by light microscopy. Sections were examined at 40–400 magnifications using an OLYMPUS CX41 microscope, and the images were scanned under equal light conditions using the CELL A computer program.

Type I and type II skeletal muscle fibers were examined through histochemical detection of cytochrome oxidase (COX) and succinate dehydrogenase (SDH) enzymes activity (Tanji & Bonilla, 2008). Gastrocnemius muscle samples were dissected from excess connective tissue, embedded in OCT compound (Tissue-Tek), and oriented so that fibers could be cut transversely. Muscle specimens were then frozen in pre-cooled isopentane in liquid nitrogen. 8 µm-thick cryosections were cut by using a Leica CM1510S Cryostat, placed on Superfrost microscope slides at -20°C, and stained for detection of COX or SDH activities. For COX stain, the sections were incubated at 37˚C for 40 min in 10 ml of 5 mM phosphate buffer (pH 7.4), containing 0.1% 3,30-diaminobenzidine (DAB), 0.1% cytochrome c (from horse heart), and 0.02% catalase, while for SDH stain, the sections were incubated at 37˚C for 7 min in 10 ml of 5 mM phosphate buffer (pH 7.4), containing 5 mM ethylenediaminetetraacetic (EDTA), 1 mM potassium cyanide (KCN), 0.2 mM phenazine methosulfate (PMS), 50 mM succinic acid, and 1.5 mM nitroblue tetrazolium (NBT). The pH of the incubation buffers was adjusted to 7.6 with potassium hydroxide (KOH), and then the buffers were filtered with filter paper n 1 before use. The sections were then rinsed 3 times for 5 min in distilled water at room temperature, mounted on glass slides with warm glycerin gel, and examined with

digital images acquired using a Carl Zeiss Primo Star Optic microscope and a Magnifier AxioCam ICc3 digital camera.

In vivo MRI and proton MRS

MRI and MRS studies were conducted using a 7 T horizontal bore magnet Bruker Biospec TM 70/20 USR designed for small animal experimentation. Mice were anesthetized by spontaneous breathing of 0.4–2.0 % (0.4-0.6 % in KI and 1.5-2.0% in WT) isofluorane and oxygen mixture through a nose cone using a MSS (Medical Suplies and Services) veterinary anesthesia unit. The mice were placed in an MRI-compatible cradle and their body temperature was maintained at 37ºC by a water bath circulation system. A pneumatic pillow and an accompanying pressure transducer monitored their respiratory rates. Both temperature and breathing were recorded by a small animal monitoring system. Anesthesia was adjusted in order to obtain a respiratory rate of about 40-60 breaths per minute. Following localizer scans, high- resolution axial and coronal T2-weighted datasets were acquired in order to visualize any cerebral or structural atrophy and to investigate for potential focal pathologies. A fast-spin echo sequence was used to acquire inter- leaved multislice T2-weighted, high-resolution mouse brain images with TR/TE = 2500 / 33 ms, field-of-view = 20 x 20 mm, slice thickness = 0.7 mm, inter-slice gap 1⁄3 0.3 mm, and 48 excitations per phase-encoding step. The data were zero-filled to yield a final image with 78 x 78 mm in-plane resolution (Diaz et al, 2012). Water-suppressed point-resolved spectroscopy was acquired in order to glean the metabolic status from the mouse brain. The MRI dataset was collected with the following settings: a voxel size of 2 × 2 × 2 mm, a bandwidth of 3 kHz, 2048 acquisition points, a TR period of 2500 ms, a TE period of 144 ms and 256 averages. The voxel was centered in the brainstem region (Diaz et al, 2012).

Transcriptome analysis by RNA-seq

The RNeasy Lipid Tissue Mini Kit (Qiagen) was used to extract total RNAs from the braintem of five animals in each experimental group. The quality and quantity of the RNAs were evaluated by gel electrophoresis and using a NanoDrop® ND-1000 Spectrophotometer. 5 μg of the total RNA of each sample were then added to a separate tube containing 100 μl of ethanol plus 10% 3 M sodium acetate, pH 5.2. The tubes were then shipped in dry ice to Macrogen Inc. (Korea) for sequencing. There, the RNAs were precipitated and their quality and quantity assessed using an Agilent Bioanalyzer 2100 and an RNA 6000 chip (Agilent Technologies). The cDNA libraries were then constructed using the TruSeq RNA Sample Prep Kit v2 (Illumina, Inc.) and their quality checked using an Agilent Bioanalyzer 2100 and a DNA 1000 chip (Agilent Technologies). The libraries were Paired End sequenced in a HiSeq 4000 system (Illumina, Inc.). We aimed for 4 to 5 Giga Bases outcome per sample. The quality of the resulting sequencing reads was assessed using FastQC. The GRCm38.p5 fasta and gtf files of the reference mouse genome were downloaded from the Ensembl database and indexed using the bwtsw option of BWA (Linden et al, 2012). BWA, combined with *xa2multi.pl* and SAMtools (Li et al, 2009), was also used for aligning the sequencing reads against the reference genome, and HTSeq was used for counting the number of reads aligned to each genomic locus (Mishanina et al, 2015). The alignments and counting were carried out in our local server following the protocols as described (Brzywczy et al, 2002; Di Meo et al, 2011).

After elimination of the genomic loci that aligned to less than 5 reads in less than 5 samples and normalization of the read counts by library size, the differential gene expression was detected using the Generalized Linear Model (glmLRT option) statistic in EdgeR (Hine et al, 2015). We used a 0.05 *p*-level threshold after False Discovery Rate correction for type I

error. The heatmap figure was made using the heatmap function in R (https://www.r-project.org/). Annotation of the differentially expressed genes was obtained from the Mouse Genome Informatics (http://www.informatics.jax.org/).

Subcellular fractionation

Mitochondrial isolation was performed as previously described (Fernandez-Vizarra et al, 2002). Tissues were homogenized in a glass–Teflon homogenizer. Brain was homogenized (1:5, w/v) in the homogenization medium A plus 0.2% free fatty acids BSA; and kidney was homogenized (1:4, w/v) in the homogenization medium A (0.32 M sucrose, 1 mM EDTA, 10 mM Tris–HCl, ph 7.4). Brain and kidney homogenates were centrifuged at 1,000*g* for 5 min at 4°C to remove nuclei and debris. Mitochondria were collected from supernatants after centrifuging at 14,400*g* for 2 min at 4 °C (twice). The final crude mitochondrial pellet was store at 80°C (Garcia-Corzo et al, 2014).

Mitochondrial respiration

To isolate fresh mitochondria, mice were sacrificed and the organs were immediately extracted and placed in ice. The brain were homogenized at a 1:10 w/v ratio in a respiration buffer C (0.32 M Sucrose, 1 mM EDTA-K^+, 10 mM Tris-HCl, pH7.4) at 500 rpm at 4 °C in a glass-teflon homogenizer. The homogenate was centrifuged at 13,000*g* for 3 min at 4 ºC. The Supernatant (s1) was kept on ice and the pellet was re-suspended in 5 ml of buffer A (250 mM Sucrose, 0.5 mM Na_2EDTA, 10 mM Tris and 1 % free fatty acid albumin) and centrifuged at 13,000*g* for 3 min at 4 ºC. The subsequent supernatant (s2) was combined with s1 and centrifuged at 21,200*g* for 10 min at 4 ºC. The resulting mitochondrial pellet from this step was re-suspended in 1 ml extraction buffer A containing 15% Percoll, poured into

ultracentrifuge tubes containing a Percoll gradient formed by 1 ml 40% Percoll and 1 ml 23% Percoll in buffer A, and centrifuged at 63,000*g* for 30 min at 4ºC. Pure mitochondria, corresponding to the fraction between 23% and 40% Percoll, were collected, washed twice with 1 ml of buffer A at 10,300*g* for 10 min at 4ºC. Mitochondrial pellets were suspended in MAS 1X medium (140 mM sucrose, 440mM mannitol, 20mM KH_2PO_4, 10mM $MgCl_2$, 4mM HEPES, 2mM EGTA). Kidneys were homogenized at 1:10 w/v ratio in a respiration buffer A (250 mM Sucrose, 0.5 mM Na_2EDTA, 10 mM Tris and 1 % free fatty acid albumin) at 800 rpm in a glass-teflon homogenizer. The homogenate was then centrifuged at 500*g* for 7 min at 4 ºC and the supernatant was centrifuged at 7,800*g* for 10 min at 4 ºC. The resulting pellet was re-suspended in respiration buffer B (250 mM Sucrose, 0.5 mM Na_2EDTA and 10 mM Tris) and an aliquot was used for protein quantification. The remaining sample was then centrifuged at 6,000*g* for 10 min at 4ºC. The pellet was resuspended in buffer A and centrifuged again at 6,000*g* for 10 min at 4ºC. The final crude mitochondrial pellet was re-suspended in MAS 1X medium (Luna-Sanchez et al, 2015).

Mitochondrial respiration was measured by using an XFe24 Extracellular Flux Analyzer (Seahorse Bioscience) (Rogers et al, 2011). Mitochondria were first diluted to the needed concentration required for plating in cold MAS 1X (3,5 μg/ in brain; 2 μg/well in kidney). Next, 50 μl of mitochondrial suspension was delivered to each well (except for background correction wells) while the plate was on ice. The plate was then centrifuged at 2,000*g* for 10 min at 4°C. After centrifugation, 450 μl of MAS 1X + substrate (10 mM succinate, 2 mM malate, 2 mM glutamate and 10 mM pyruvate) was added to each well. Respiration by the mitochondria was sequentially measured in a coupled state with the substrate present (basal respiration or State 2), followed by State 3o (phosphorylating respiration, in the presence of ADP and substrates); State 4 (non-phosphorylating or resting respiration) was measured after addition of oligomycin when all ADP was consumed, and then maximal uncoupler-

stimulated respiration (State 3u). Injections were as follows: port A, 50 μl of 40 mM ADP (4 mM final); port B, 55 μl of 30 μg/ml oligomycin (3 μg/ml final); port C, 60 ul of 40 uM FCCP (4 μM final); and port D, 65 μl of 40 μM antimycin A (4 μM final). All data were expressed in pmol/min/mg protein.

CoQ-dependent respiratory chain activities

CoQ dependent respiratory chain activities were measured in submitochondrial particles. To prepare submitochondrial particles, each mitochondrial pellet (100 μg prots) was suspended and sonicated in 100 μl of 0.1 M potassium phosphate buffer, pH 7.5. Complex I + III activity was measured at 30 °C in the presence of 0.5 mM potassium cyanide, 0.2 mM NADH and 0.1 mM cytochrome c, as the rotenone-sensitive reduction of cytochrome c at 550 nm (Garcia-Corzo et al, 2014). The results were expressed in nmol reduced cyt c/min/mg prot. Complex II+III activity was measured at 30 °C in the presence of 0.5 mM KCN, 0.3 mM succinate and 0.01 mM rotenone. The reaction was initiated by addition of 0.1 mM cytochrome c and decrease in absorbance was monitored at 550 nm. To test whether β-RA works as a CoQ substitute in the mitochondrial respiratory chain, we also measure the CI-III activity after adding 50 μM β-RA into the reaction mix. The results were expressed in nmol reduced cyt c/min/mg prot.

Evaluation of supercomplexes formation by BNGE

Blue native gel electrophoresis (BNGE) was performed on crude mitochondrial fractions from mice kidneys and brain. Mitochondrial isolation was performed as previously described (Fernandez-Vizarra et al, 2002). One aliquot of the crude mitochondrial fraction was used for

protein determination. The remaining samples were then centrifuged at 13,000*g* for 3 min at 4 °C. The mitochondrial pellets were suspended in an appropriate volume of medium C (1 M aminocaproic acid, 50 mM Bis-Tris-HCl [pH 7.0]) to create a protein concentration of 10 mg/ml, and the membrane proteins were solubilized with digitonin (4 g/g) and incubated for 10 min in ice. After centrifugation for 30 min at 13,000*g* (4 °C), the supernatant were collected and 3 µl of 5% Brilliant Blue G dye, prepared in 1 M aminocaproic acid, was added. Mitochondrial proteins (100 µg) were then loaded and run on a 3–13% gradient native gel as previously described (Acin-Perez et al, 2008). After electrophoresis, the complexes were electroblotted onto PVDF membranes and sequentially tested with specific antibodies against CI, anti-NDUFA9 (Abcam, ab14713, dilution 1:5,000), CIII, anti-ubiquinol-cytochrome c reductase core protein I (Abcam, ab110252, dilution 1:5,000) and VDAC1 (Abcam, ab14734, dilution 1:5,000).

Sample preparation and western blot analysis in tissues

For Western blot analyses in mouse tissues, samples were homogenized in T-PER® buffer (Thermo Scientific) with protease inhibitor cocktail (Pierce) at 1,100 rpm in a glass–Teflon homogenizer. Homogenates were sonicated and centrifuged at 1,000*g* for 5 min at 4°C, and the resultant supernatants were used for Western blot analysis. For Western blot analyses in brain mitochondria, the pellets containing the mitochondrial fraction were re-suspended in RIPA buffer with protease inhibitor cocktail. 60 µg of protein from the sample extracts was electrophoresed in 12% Mini-PROTEAN TGX™ precast gels (Bio-Rad) using the electrophoresis system mini-PROTEAN Tetra Cell (Bio-Rad). Proteins were transferred onto PVDF 0.45-µm membranes using a mini Trans-blot Cell (Bio-Rad) or Trans-blot Cell (Bio-Rad) and probed with target antibodies. Protein–antibody interactions were detected with

peroxidase-conjugated horse antimouse, anti-rabbit, or anti-goat IgG antibodies using Amersham ECL™ Prime Western Blotting Detection Reagent (GE Healthcare, Buckinghamshire, UK). Band quantification was carried out using an Image Station 2000R (Kodak, Spain) and a Kodak 1D 3.6 software. Protein band intensity was normalized to VDAC1 (mitochondrial protein), TOM20 (mitochondrial protein) or GAPDH (cytosolic protein), and the data were expressed in terms of percent relative to wild-type mice or control cells (Luna-Sanchez et al, 2015; Luna-Sanchez et al, 2017).

The following primary antibodies were used: anti-SQRDL (Proteintech, 17256-1-AP, dilution 1:500), anti-PDSS2 (Proteintech, 13544-1-AP, dilution 1:500), anti-COQ2 (Origene, TA341982, dilution 1:2,500), anti-COQ4 (Proteintech, 16654-1-AP, dilution 1:2,000), anti-COQ5 (Proteintech, 17453-1-AP, dilution 1:1,000), anti-COQ6 (Proteintech, 12481-1-AP, dilution 1:500), anti-COQ7 (Proteintech, 15083-1-AP, dilution 1:1,000), anti-COQ8A (Proteintech, 15528-1-AP, dilution 1:2,500), anti-S6R (Cell Signaling, 2217, dilution 1:1,000), anti-S6RP (Cell Signaling, 2211, dilution 1:1,000), anti-FGF21 (Abcam, ab171941, dilution 1:1,000), anti-VDAC1 (Abcam, ab14734, dilution 1:5,000), anti-TOM20 (Proteintech, 11802-1-AP, dilution 1:5,000) and anti-GAPDH (Santacruz, sc-166574, dilution 1:200).

Quantification of CoQ_9 and CoQ_{10} levels in mice tissues

CoQ_9 and CoQ_{10} levels were determined via reversed phase HPLC coupled to electrochemical (EC) detection (Garcia-Corzo et al, 2013; Lopez et al, 2010). The results were expressed in ng CoQ/mg prot.

Quantification of β-RA levels and screening of potential derivate molecules in mice tissues

To measure the amount of β-RA that reaches each tissue after the treatment, we treated *Coq9*^{R239X} mice with β-RA in the chow at a dose of 2500 mg/kg bw/day during two weeks, and mice were sacrificed at 7:30 -9:00 am. For an acute evaluation, we treated the mutant mice with an intraperitoneal (i.p.) injection of β-RA in a dose of 250 mg/kg bw, and mice were sacrificed 30 minutes after the injection. Blood samples were extracted and centrifuged to obtain the plasma before and after the treatment. Brain, kidney and liver were homogenated in water. The homogenate and the plasma samples were treated with a solution of methanol/water (80:20, v/v), shaked for 1 minute, sonicated for 15 minutes and then centrifuged at 5,000*g* for 25 minutes at 4ºC (Borges et al, 2017). The supernatants were analyzed by HPLC-UV using an Ultra Aqueous C18 column and a mobile phase consisting of 20 mM potassium phosphate and 15% acetonitrile (pH 2.5). The standard curve consisted of β-RA at a concentration of 5, 25 and 100 μg /ml. The results were expressed in μg of β-RA/mg protein.

The supernatants were also analyzed using a Waters Acquity UPLCTM H-Class (Waters, Manchester, UK), consisting of an ACQUITY UPLCTM binary solvent manager and an ACQUITY UPLCTM sample manager coupled to a Xevo TQS detector of mass spectrometer (MS/MS) with an electrospray ionization in negative mode (Waters, Manchester, UK). The analytical separation column was a BEH C18, 1.7 μm, 2.1 × 50 mm column (Waters, Spain) (Garcia-Corzo et al, 2013) and the flow rate was 0.4 ml/min. The mobile phase consisted of two solutions: eluent A (0.1% Formic acid) and eluent B (acetonitrile). Samples were eluted over 10 min with a gradient as follow: 0-7 min, 95% eluent A; 7-7.1 min, 53 % eluent A; 7.1-8 min, 100 % eluent B; 8-10 min, 95% eluent A. Source and desolvation temperatures were set at 140 and 500 °C, respectively. Nitrogen was used as both cone gas (150 l/h) and desolvation gas (500 l/h), and argon was used as collision gas (0.15 ml/min). Mass spectrometry analyses were carried out in full scan mode between 100 and 300 uma. To

quantify the levels of 4-hydroxybenzoic acid (4-HB), we use a standard at a concentration of 10 μg /ml and analyzed in a MS/MS mode.

Quantification of pro-inflammatory mediators in murine RAW 264.7 macrophages and brainstem extracts

Concentrations of interleukin (IL)-1β, IL-2, interferon-γ (IFN-γ), tumor necrosis factor-α (TNF-α) and growth regulated oncogene-α (GRO-α) were quantified in duplicates in the supernatant of the cell culture of murine RAW 264.7 macrophages and brainstem tissue using ProcartaPlexTM Multiplex immunoassays (eBioscience), as previously described and according to the manufacturer instructions (Hinz et al, 2000; Reichmann et al, 2015). The macrophages were stimulated 1 μg/ml LPS *E. Coli* 0111:B4 and the supernatants were collected after 24 h of incubation. 6 h prior to the stimulation with LPS, the cells were preincubated with 1 mM β-RA. As control of the experimental procedure, we collected the cells after 24 h of LPS stimulation and quantify iNOS mRNA levels by RT-PCR using a Taqman probe (Mm00440502_m1 *Nos2*, Applied Biosystems). Target-specific fluorescent signals were measured with the Luminex 200 multiplex suspension array system. Brainstem samples were used at a concentration of 10 mg/ml. The results were expressed in pg/ml (cell culture supernatant) or pg/mg prot (brainstem samples).

Hemogram and steroid hormones quantification

Blood samples were collected in heparin tubes using a golden rod lancet and the submandibular vein of mice as puncture site. The hemograms were obtained using a MYTHIC 22 CT in an automated hematology analyzer.

The plasma was extracted from blood samples by centrifugation at 4,500*g* for 10 minutes at 4ºC. The steroid hormones were quantified using 50 µL of plasma and liquid chromatography-triple quadrupole mass spectrometric measurements. Details of the method have been reported previously reported (Haller et al, 2010).

Statistical analysis

Number of animals in each group were calculated in order to detect gross ~60% changes in the biomarkers measurements (based upon alpha=0.05 and power of beta=0.8). We used the application available in: http://www.biomath.info/power/index.htm. Animals were genotyped and randomly assigned in experimental groups in separate cages by the technician of the animal facility. Most statistical analyses were performed using the Prism 6 scientific software. Data are expressed as the mean ± SD of six-ten experiments per group. A one-way ANOVA with a Tukey's post hoc test was used to compare the differences between three experimental groups. Studies with two experimental groups were evaluated using unpaired Student's t-test. A P-value of < 0.05 was considered to be statistically significant. A Mann-Whitney test was used for the statistic analysis of the hormones measurements. Survival curve was analyzed by log-rank (Mantel-Cox) and the Gehan-Breslow-Wilcoxon tests. The statistic in the transcriptomics analysis is described in its section.

SUPPLEMETAL REFERENCES

Acin-Perez R, Fernandez-Silva P, Peleato ML, Perez-Martos A, Enriquez JA (2008) Respiratory active mitochondrial supercomplexes. Mol Cell 32**:** 529-539

Borges TH, López LC, Pereira JA, Cabrera–Vique C, Seiquer I (2017) Comparative analysis of minor bioactive constituents (CoQ 10 , tocopherols and phenolic compounds) in

Arbequina extra virgin olive oils from Brazil and Spain. Journal of Food Composition and Analysis 63: 47-54

Brzywczy J, Sienko M, Kucharska A, Paszewski A (2002) Sulphur amino acid synthesis in Schizosaccharomyces pombe represents a specific variant of sulphur metabolism in fungi. Yeast 19: 29-35

Di Meo I, Fagiolari G, Prelle A, Viscomi C, Zeviani M, Tiranti V (2011) Chronic exposure to sulfide causes accelerated degradation of cytochrome c oxidase in ethylmalonic encephalopathy. Antioxid Redox Signal 15: 353-362

Diaz F, Garcia S, Padgett KR, Moraes CT (2012) A defect in the mitochondrial complex III, but not complex IV, triggers early ROS-dependent damage in defined brain regions. Hum Mol Genet 21: 5066-5077

Diaz-Casado ME, Rusanova I, Aranda P, Fernandez-Ortiz M, Sayed RKA, Fernandez-Gil BI, Hidalgo-Gutierrez A, Escames G, Lopez LC, Acuna-Castroviejo D (2018) In Vivo Determination of Mitochondrial Respiration in 1-Methyl-4-Phenyl-1,2,3,6-Tetrahydropyridine-Treated Zebrafish Reveals the Efficacy of Melatonin in Restoring Mitochondrial Normalcy. Zebrafish 15: 15-26

Fernandez-Vizarra E, Lopez-Perez MJ, Enriquez JA (2002) Isolation of biogenetically competent mitochondria from mammalian tissues and cultured cells. Methods 26: 292-297

Garcia-Corzo L, Luna-Sanchez M, Doerrier C, Garcia JA, Guaras A, Acin-Perez R, Bullejos-Peregrin J, Lopez A, Escames G, Enriquez JA et al (2013) Dysfunctional Coq9 protein causes predominant encephalomyopathy associated with CoQ deficiency. Hum Mol Genet 22: 1233-1248

Garcia-Corzo L, Luna-Sanchez M, Doerrier C, Ortiz F, Escames G, Acuna-Castroviejo D, Lopez LC (2014) Ubiquinol-10 ameliorates mitochondrial encephalopathy associated with CoQ deficiency. Biochim Biophys Acta 1842: 893-901

Gomez-Guzman M, Jimenez R, Romero M, Sanchez M, Zarzuelo MJ, Gomez-Morales M, O'Valle F, Lopez-Farre AJ, Algieri F, Galvez J et al (2014) Chronic hydroxychloroquine improves endothelial dysfunction and protects kidney in a mouse model of systemic lupus erythematosus. Hypertension 64: 330-337

Haller F, Prehn C, Adamski J (2010) Quantification of steroids in human and mouse plasma using online solid phase extraction coupled to liquid chromatography tandem mass spectrometry. Protocol Exchange

Hine C, Harputlugil E, Zhang Y, Ruckenstuhl C, Lee BC, Brace L, Longchamp A, Trevino-Villarreal JH, Mejia P, Ozaki CK et al (2015) Endogenous hydrogen sulfide production is essential for dietary restriction benefits. Cell 160: 132-144

Hinz B, Kraus V, Pahl A, Brune K (2000) Salicylate metabolites inhibit cyclooxygenase-2-dependent prostaglandin E(2) synthesis in murine macrophages. Biochem Biophys Res Commun 274: 197-202

Li H, Handsaker B, Wysoker A, Fennell T, Ruan J, Homer N, Marth G, Abecasis G, Durbin R, Genome Project Data Processing S (2009) The Sequence Alignment/Map format and SAMtools. Bioinformatics 25: 2078-2079

Linden DR, Furne J, Stoltz GJ, Abdel-Rehim MS, Levitt MD, Szurszewski JH (2012) Sulphide quinone reductase contributes to hydrogen sulphide metabolism in murine peripheral tissues but not in the CNS. Br J Pharmacol 165: 2178-2190

Lopez LC, Quinzii CM, Area E, Naini A, Rahman S, Schuelke M, Salviati L, DiMauro S, Hirano M (2010) Treatment of CoQ(10) deficient fibroblasts with ubiquinone, CoQ analogs, and vitamin C: time- and compound-dependent effects. PLoS One 5: e11897

Luna-Sanchez M, Diaz-Casado E, Barca E, Tejada MA, Montilla-Garcia A, Cobos EJ, Escames G, Acuna-Castroviejo D, Quinzii CM, Lopez LC (2015) The clinical heterogeneity of coenzyme Q10 deficiency results from genotypic differences in the Coq9 gene. EMBO Mol Med 7: 670-687

Luna-Sanchez M, Hidalgo-Gutierrez A, Hildebrandt TM, Chaves-Serrano J, Barriocanal-Casado E, Santos-Fandila A, Romero M, Sayed RK, Duarte J, Prokisch H et al (2017) CoQ deficiency causes disruption of mitochondrial sulfide oxidation, a new pathomechanism associated with this syndrome. EMBO Mol Med 9: 78-95

Mishanina TV, Libiad M, Banerjee R (2015) Biogenesis of reactive sulfur species for signaling by hydrogen sulfide oxidation pathways. Nat Chem Biol 11: 457-464

Quintana A, Kruse SE, Kapur RP, Sanz E, Palmiter RD (2010) Complex I deficiency due to loss of Ndufs4 in the brain results in progressive encephalopathy resembling Leigh syndrome. Proc Natl Acad Sci U S A 107: 10996-11001

Reichmann F, Hassan AM, Farzi A, Jain P, Schuligoi R, Holzer P (2015) Dextran sulfate sodium-induced colitis alters stress-associated behaviour and neuropeptide gene expression in the amygdala-hippocampus network of mice. Sci Rep 5: 9970

Rogers GW, Brand MD, Petrosyan S, Ashok D, Elorza AA, Ferrick DA, Murphy AN (2011) High throughput microplate respiratory measurements using minimal quantities of isolated mitochondria. PLoS One 6: e21746

Tanji K, Bonilla E (2008) Light microscopic methods to visualize mitochondria on tissue sections. Methods 46: 274-280
