# Supplementary material for: β‐RA reduces DMQ/CoQ ratio and rescues the encephalopathic phenotype in Coq9 R239X mice
Source: EMBO Mol Med. 2018 Nov 27;11(1):e9466. doi: 10.15252/emmm.201809466 (PMC6328940; doi:10.15252/emmm.201809466)
Supplement: Supplementary file 2 — Expanded View Figures PDF [file EMMM-11-e9466-s002.pdf]

Expanded View Figures

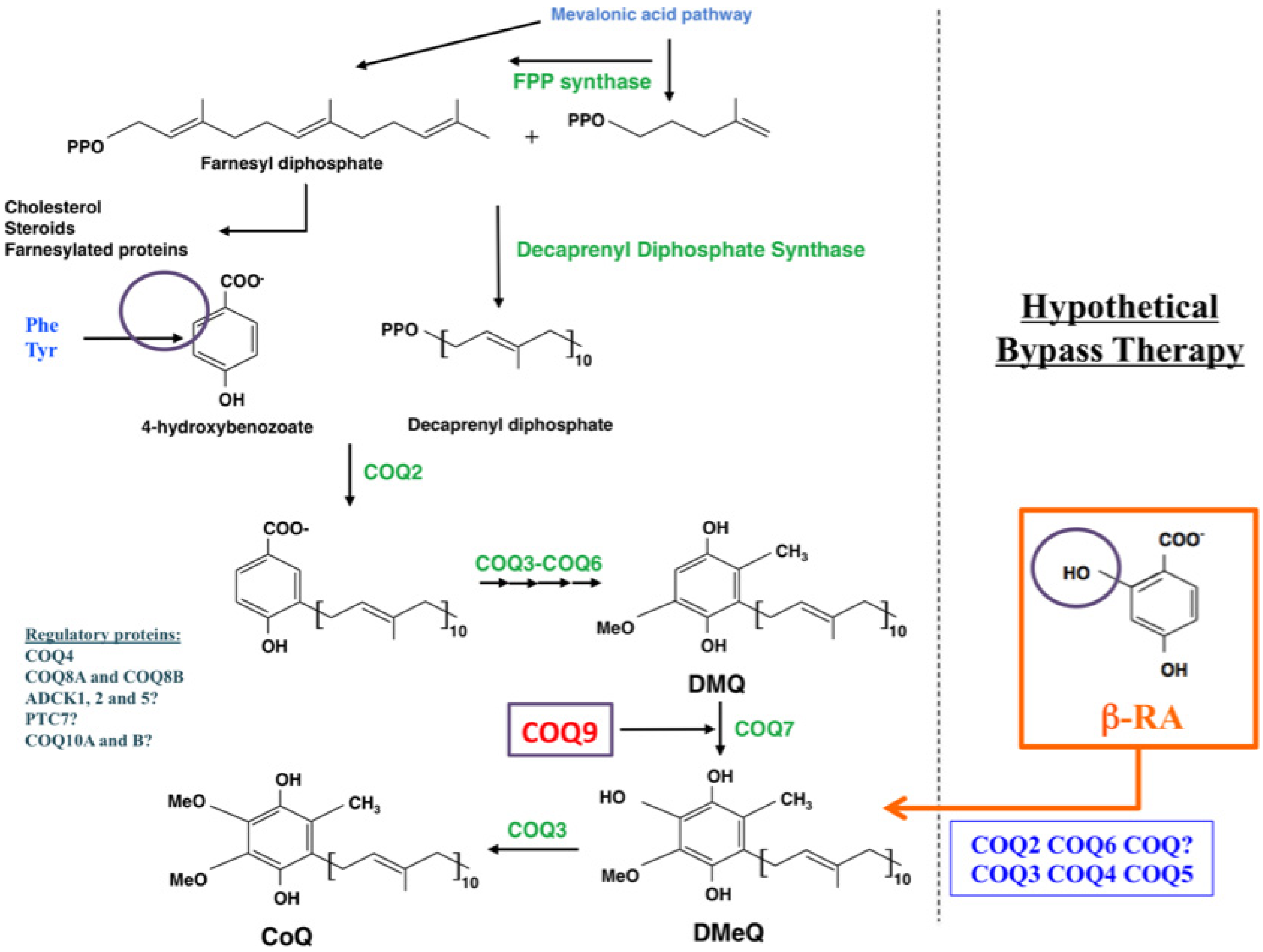

Figure EV1. CoQ biosynthetic pathway, including the bypass hypothesis for defects in COQ9 or COQ7.

4-HB = 4-hydroxybenzoic acid; DMQ = demethoxyubiquinone or 2-polyprenyl-6-methoxy-3-methyl-1,4-benzoquinone; DMeQ = 2-polyprenyl-5-hydroxy-6-methoxy-3-methyl-1,4-benzoquinone; β-RA = β-resorcylic acid. The purple circle indicates the OH group present in the β-RA and absent in the 4-HB. The hydroxylation of DMQ is normally catalyzed by COQ7, which needs COQ9 for its stability and activity. This step is compromised in patients with defects in COQ7 or COQ9. The pathway is exampled with the production of CoQ_{10}.

© 2018 The Authors

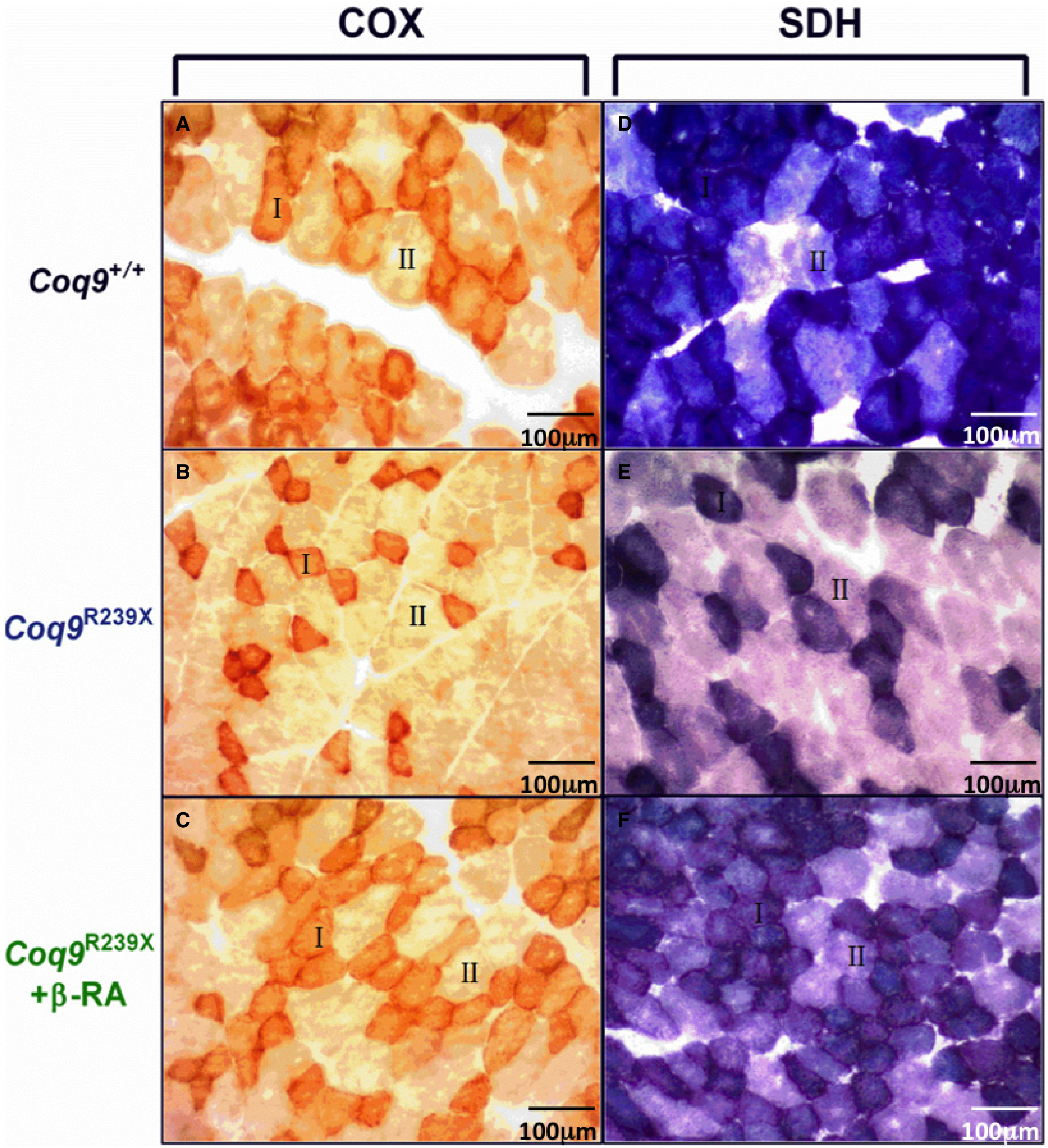

Figure EV2. COX and SDH staining in *gastrocnemius*.

A–C COX stains in *gastrocnemius* of *Coq9*$^{+/+}$ mice (A), *Coq9*^{R239X} mice (B), and *Coq9*^{R239X} mice after β-RA treatment (C).

D–F SDH stain in *gastrocnemius* of *Coq9*$^{+/+}$ mice (D), *Coq9*^{R239X} mice (E), and *Coq9*^{R239X} mice after β-RA treatment (F).

Data information: "I" indicated fiber types I and "II" indicates fiber types II.

 © 2018 The Authors
