# Supplementary material for: β‐RA reduces DMQ/CoQ ratio and rescues the encephalopathic phenotype in Coq9 R239X mice
Source: EMBO Mol Med. 2018 Nov 27;11(1):e9466. doi: 10.15252/emmm.201809466 (PMC6328940; doi:10.15252/emmm.201809466)
Supplement: Supplementary file 3 — Table EV1 [file EMMM-11-e9466-s003.docx]

**Table EV1.** **Relative** **levels CoQ_9_ and DMQ_9_/CoQ_9_ ratio in tissues of mice treated with** **β-RA.**

|  | | **1 g of β-RA/kg b.w./day - 2 months after treatment** | | | |
| --- | --- | --- | --- | --- | --- |
|  |  | ***Coq9^+/+^*** | ***Coq9^R239X^*** | ***Coq9^R239X^* + β-RA** | **Fold change** |
| CoQ_9_ (% relative to *Coq9^+/+^*) | Brain | **100** | 14.34 ± 2.51 | 9.07 ± 2.36 | 0.63 |
|  | Kidney | **100** | 2.00 ± 0.47 | 6.87 ± 1.29 | 3.43 |
|  | Skeletal muscle | **100** | 7.85 ± 1.47 | 10.58 ± 4.26 | 1.34 |
| DMQ_9_/CoQ_9_ | Brain | **0** | 1.21 ± 0.23 | 1.97 ± 0.71 | 1.63 |
|  | Kidney | **0** | 6.14 ± 0.6 | 0.65 ± 0.26 | 0.11 |
|  | Skeletal muscle | **0** | 0.65 ±0.09 | 0 | 0 |
|  | | **1 g of β-RA/kg b.w./day - 9 months after treatment** | | | |
|  |  | ***Coq9^+/+^*** | ***Coq9^R239X^*** | ***Coq9^R239X^* + β-RA** | **Fold change** |
| CoQ_9_ (% relative to *Coq9^+/+^*) | Brain | **100** | - | 9.91 ± 4.16 | - |
|  | Kidney | **100** | - | 7.29 ± 2.8 | - |
|  | Skeletal muscle | **100** | - | 8.28 ± 2.36 | - |
| DMQ_9_/CoQ_9_ | Brain | **0** | - | 1.06 ± 0.5 | - |
|  | Kidney | **0** | - | 0.32 ± 0.07 | - |
|  | Skeletal muscle | **0** | - | 0 | - |
|  | | **2 g of β-RA*kg b.w./day - 1 month after treatment** | | | |
|  |  | ***Coq9^+/+^*** | ***Coq9^R239X^*** | ***Coq9^R239X^* + β-RA** | **Fold change** |
| CoQ_9_ (% relative to *Coq9^+/+^*) | Brain | **100** | 11.47 ± 6.47 | 8.53 ± 2.56 | 0.74 |
|  | Kidney | **100** | 3.79 ± 2.27 | 9.67 ± 5 | 2.55 |
|  | Skeletal muscle | **100** | 8.96 ± 3.58 | 15.95 ± 7.11 | 1.78 |
| DMQ_9_/CoQ_9_ | Brain | **0** | 0.92 ± 0.48 | 0.87 ± 0.17 | 0.95 |
|  | Kidney | **0** | 6.59 ± 0.96 | 0.26 ± 0.13 | 0.04 |
|  | Skeletal muscle | **0** | 0.87 ± 0.61 | 0 | 0 |

The measurements were done in tissues of mice under three experimental conditions: 1) two months after the treatment with β-RA at a dose of 1 g of β-RA/kg b.w./day (upper segment of the table); 2) nine months after the treatment with β-RA at a dose of 1 g of β-RA/kg b.w./day (intermediate segment of the table); and 3) one month after the treatment with β-RA at a dose of 2 g of β-RA/kg b.w./day (lower segment of the table). The fold change values have been calculated by dividing the results in *Coq9^R239X^* mice and *Coq9^R239X^* mice treated with β-RA.
