# Supplementary material for: β‐RA reduces DMQ/CoQ ratio and rescues the encephalopathic phenotype in Coq9 R239X mice
Source: EMBO Mol Med. 2018 Nov 27;11(1):e9466. doi: 10.15252/emmm.201809466 (PMC6328940; doi:10.15252/emmm.201809466)
Supplement: Supplementary file 4 — Table EV2 [file EMMM-11-e9466-s004.docx]

**Table EV2.** **Levels of** **β-RA in plasma and tissues of the mutant mice after oral or i.p. administration of β-RA.**

| **Genotype** | **Administration Route** | **Time of sample collection** | **Number of samples with detected levels of β-RA** | **Type of Samples** | **Levels of β-RA**  **(ng/mg prot)** |
| --- | --- | --- | --- | --- | --- |
| *Coq9^R239X^* | not treated | 7:30-9:00 | 0/3 | Brain | UND |
| *Coq9^R239X^* | oral | 7:30-9:00 | 3/8 | Brain | 21.3 ± 1.32 |
| *Coq9^R239X^* | i.p. | 30 min after injection | 3/3 | Brain | 14.49 ± 2.17 |
| *Coq9^R239X^* | not treated | 7:30-9:00 | 0/3 | Liver | UND |
| *Coq9^R239X^* | oral | 7:30-9:00 | 6/8 | Liver | 37.34 ± 14.28 |
| *Coq9^R239X^* | i.p. | 30 min after injection | 3/3 | Liver | 20.91 ± 6.21 |
| *Coq9^R239X^* | not treated | 7:30-9:00 | 1/3 | Kidney | 21.9 |
| *Coq9^R239X^* | oral | 7:30-9:00 | 8/8 | Kidney | 163.08 ± 133.84 |
| *Coq9^R239X^* | i.p. | 30 min after injection | 3/3 | Kidney | 89.74 ±34.81 |
| *Coq9^R239X^* | not treated | 7:30-9:00 | 0/2 | Plasma | UND |
| *Coq9^R239X^* | oral | 7:30-9:00 | 5/5 | Plasma | 360.71 ± 265.29 |
| *Coq9^R239X^* | i.p. | 30 min after injection | 3/3 | Plasma | 116.3 ±13.62 |

i.p. = intraperitoneal; UND = undetected
