# Supplementary material for: β‐RA reduces DMQ/CoQ ratio and rescues the encephalopathic phenotype in Coq9 R239X mice
Source: EMBO Mol Med. 2018 Nov 27;11(1):e9466. doi: 10.15252/emmm.201809466 (PMC6328940; doi:10.15252/emmm.201809466)
Supplement: Supplementary file 5 — Movie EV1 [file EMMM-11-e9466-s005.zip › EMM-2018-09466_Movie_EV1/EMM-2018-09466_Legend_Movie_EV1.docx]

**EXPANDED VIEW MOVIES**

Movie EV1. Video that shows the difference between a *Coq9^R239X^* mouse and a *Coq9^R239X^* mouse after β-RA treatment, both males at 5 months of age.
