# Supplementary material for: β‐RA reduces DMQ/CoQ ratio and rescues the encephalopathic phenotype in Coq9 R239X mice
Source: EMBO Mol Med. 2018 Nov 27;11(1):e9466. doi: 10.15252/emmm.201809466 (PMC6328940; doi:10.15252/emmm.201809466)

Figure S12E. FGF21 Pre and mature form in brain of wild-type and mutant mice with and without treatment.

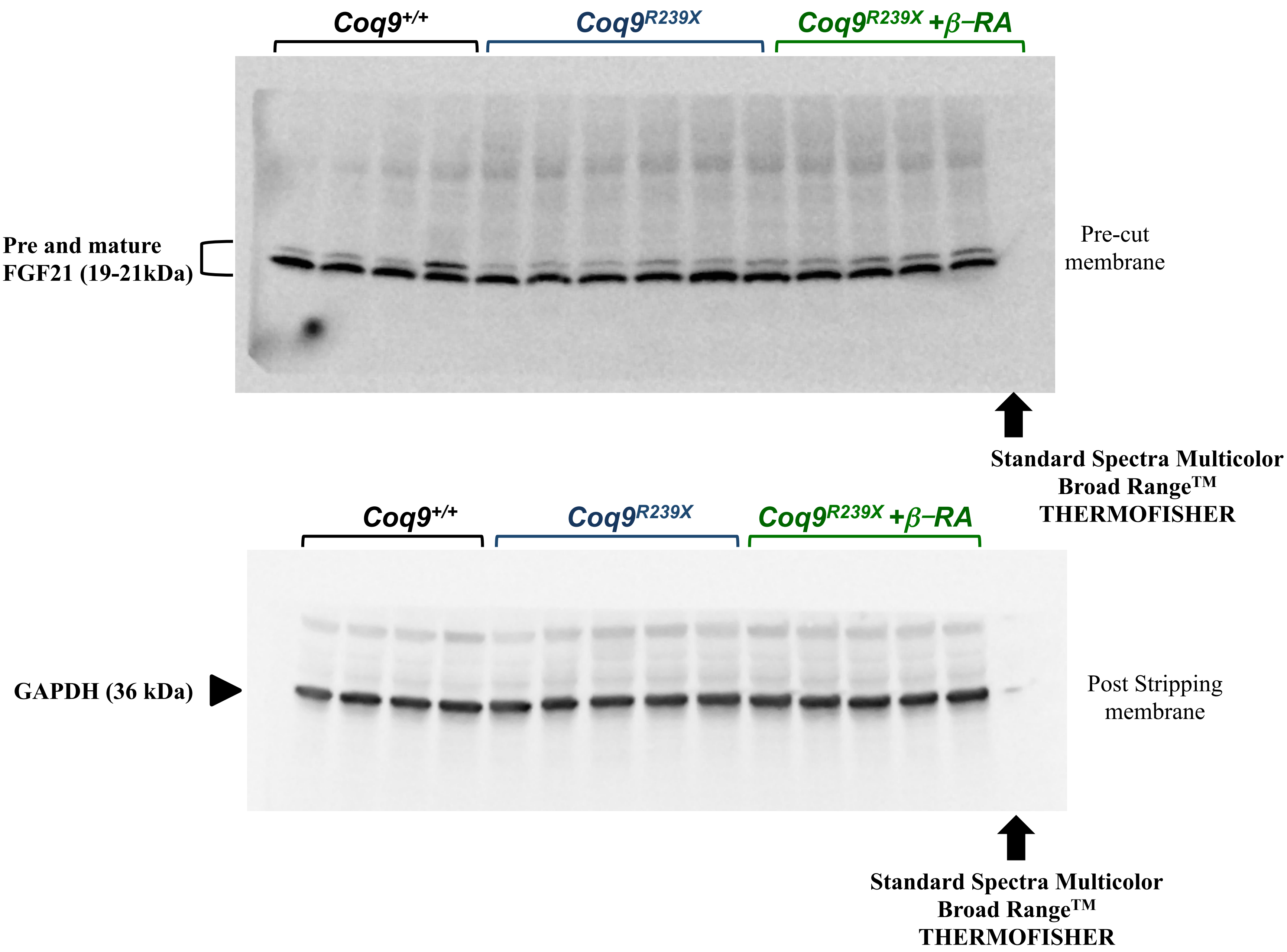

Figure S12F. FGF21 Pre and mature form in liver of wild-type and mutant mice with and without treatment.

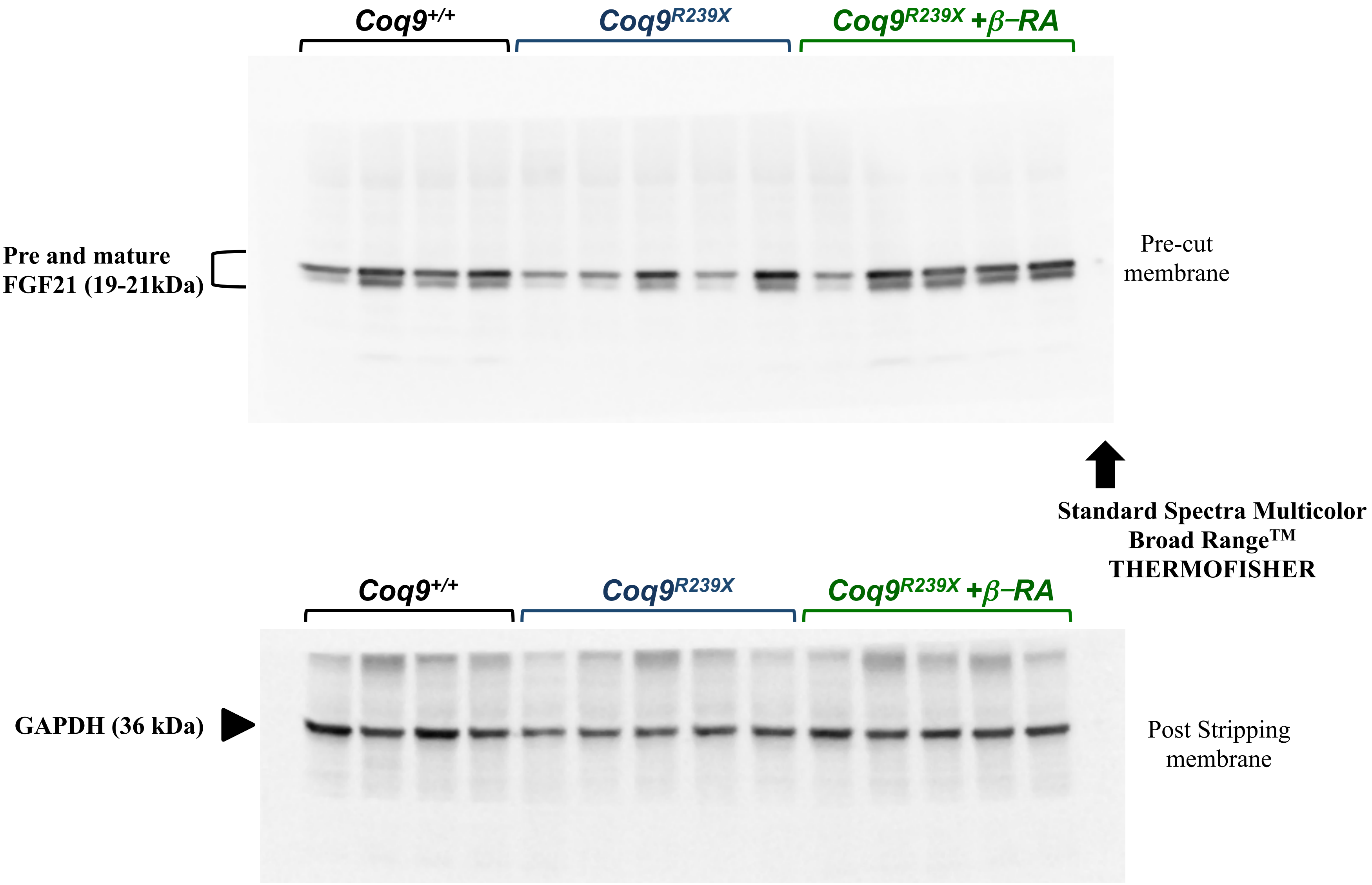

Supplement: Supplementary file 6 — Source Data for Appendix [file EMMM-11-e9466-s008.zip › EMM-2018-0946-Appendix_SourceData-/EMM-2018-09466_SD_FigS12.pdf]
