# Supplementary material for: β‐RA reduces DMQ/CoQ ratio and rescues the encephalopathic phenotype in Coq9 R239X mice
Source: EMBO Mol Med. 2018 Nov 27;11(1):e9466. doi: 10.15252/emmm.201809466 (PMC6328940; doi:10.15252/emmm.201809466)
Supplement: Supplementary file 6 — Source Data for Appendix [file EMMM-11-e9466-s008.zip › EMM-2018-0946-Appendix_SourceData-/EMM-2018-09466_SD_FigS3.pdf]

Figure S3A. S6R in brain of wild-type and mutant mice with and without treatment.

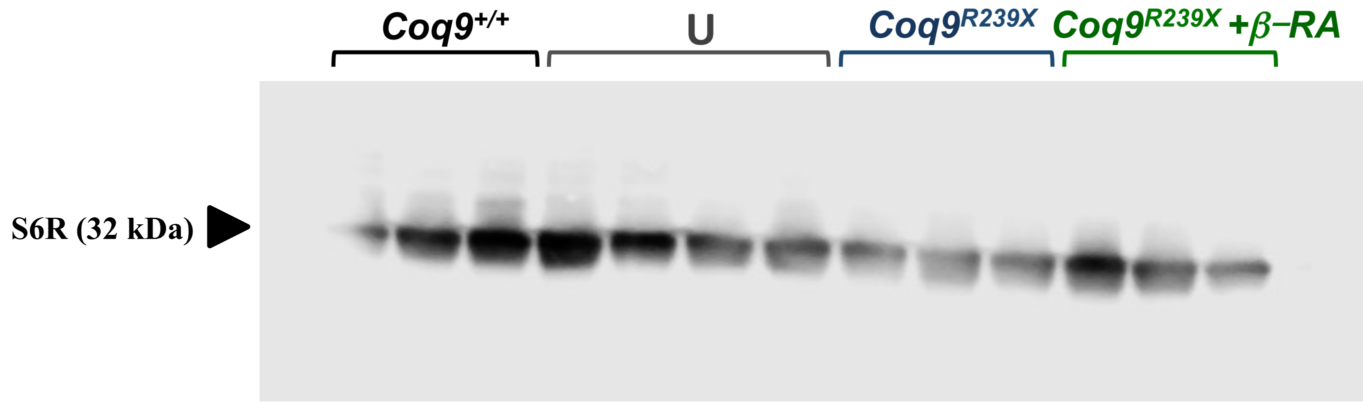

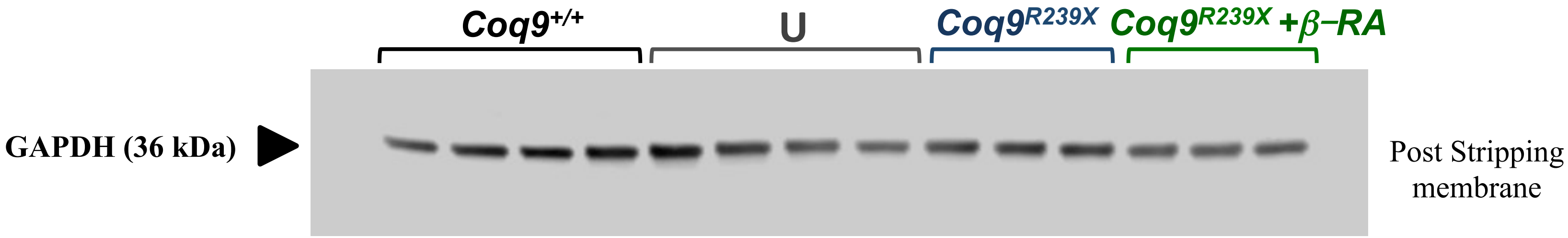

Note: lines 3, 4 and 9-14 are represented in Figure S3A in the main text.

U=Unrelated to this study

Figure S3A. S6RP in brain of wild-type and mutant mice with and without treatment.

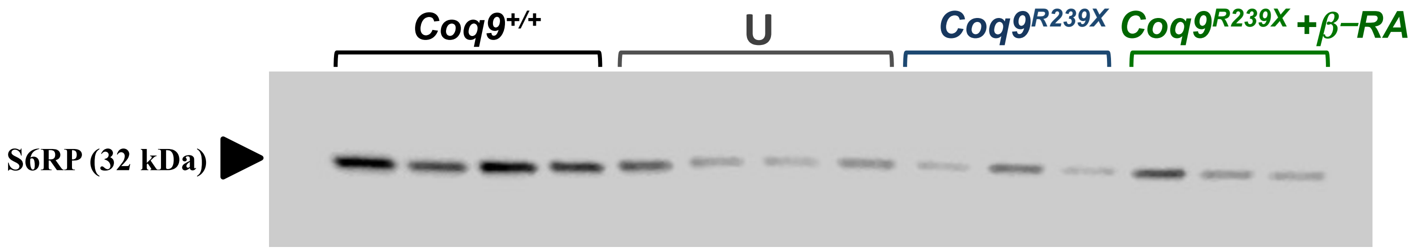

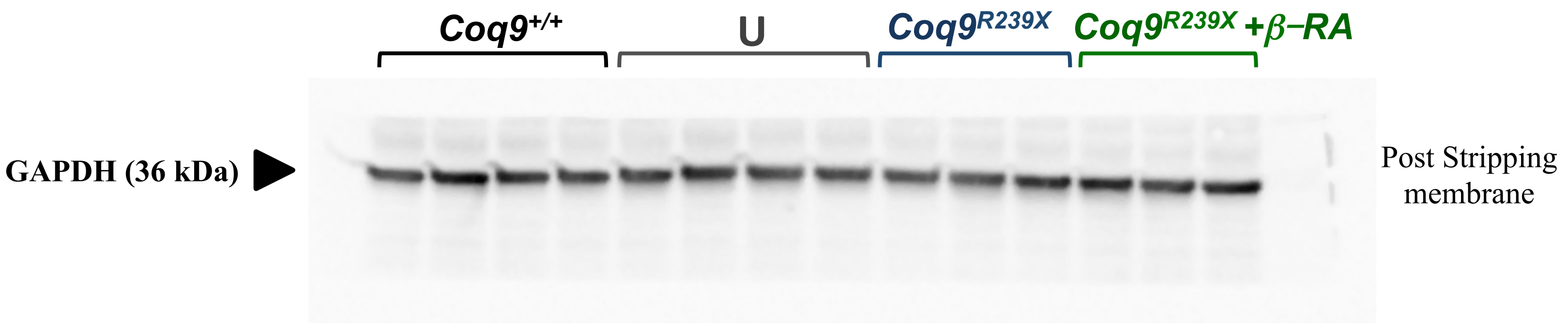

Note: lines 3, 4 and 9-14 are represented in Figure S3A in the main text.

U=Unrelated to this study

Figure S3B. S6R in kidney of wild-type and mutant mice with and without treatment.

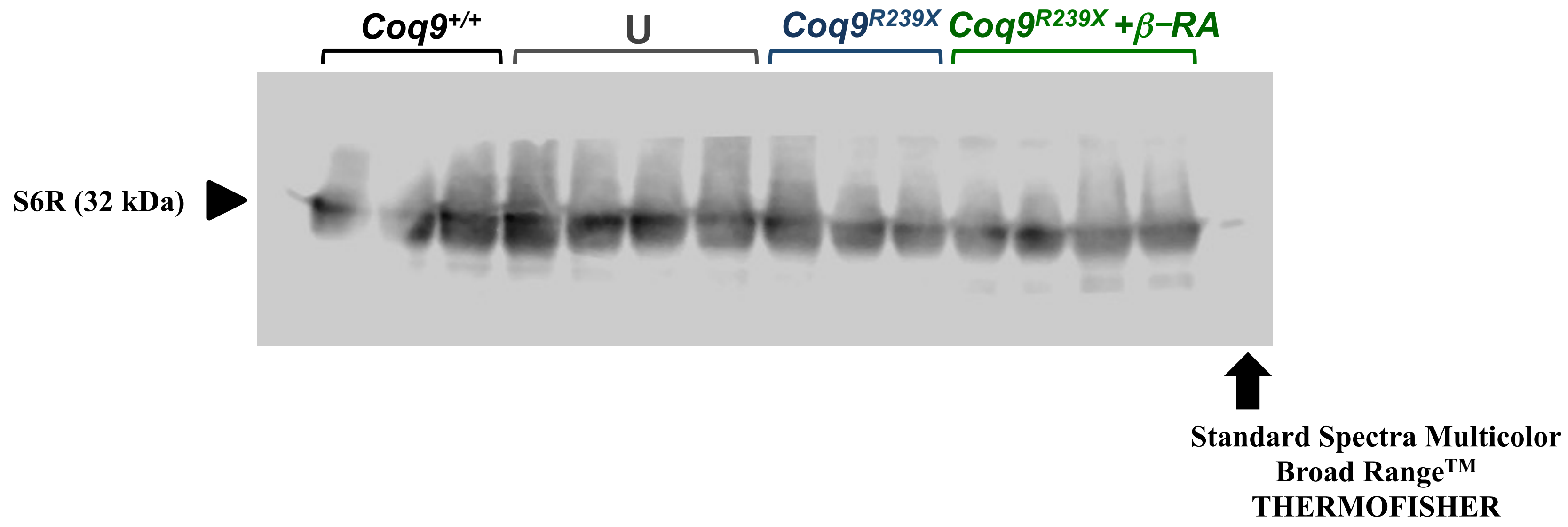

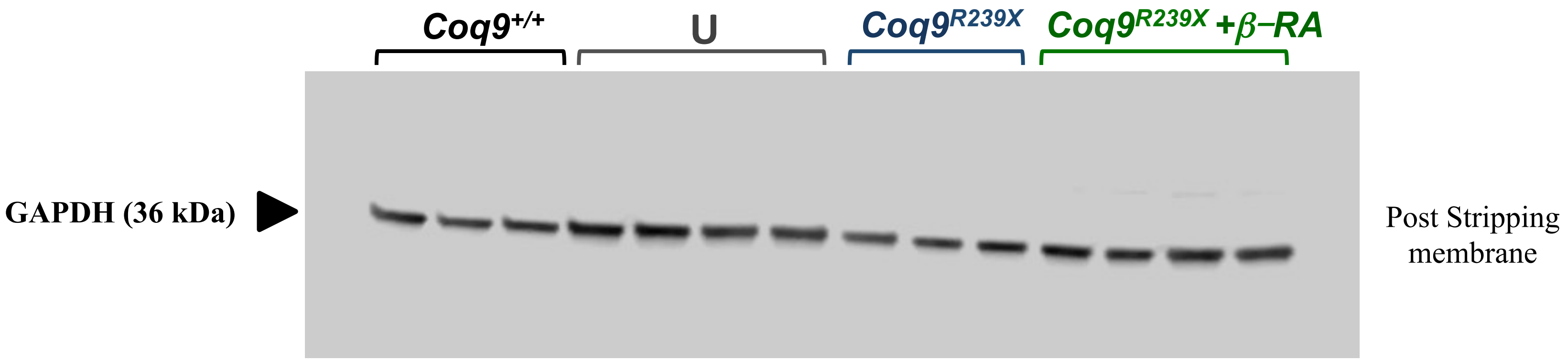

Note: lines 3 and 8-14 are represented in Figure S3B in the main text.

U=Unrelated to this study

Figure S3B. S6RP in kidney of wild-type and mutant mice with and without treatment.

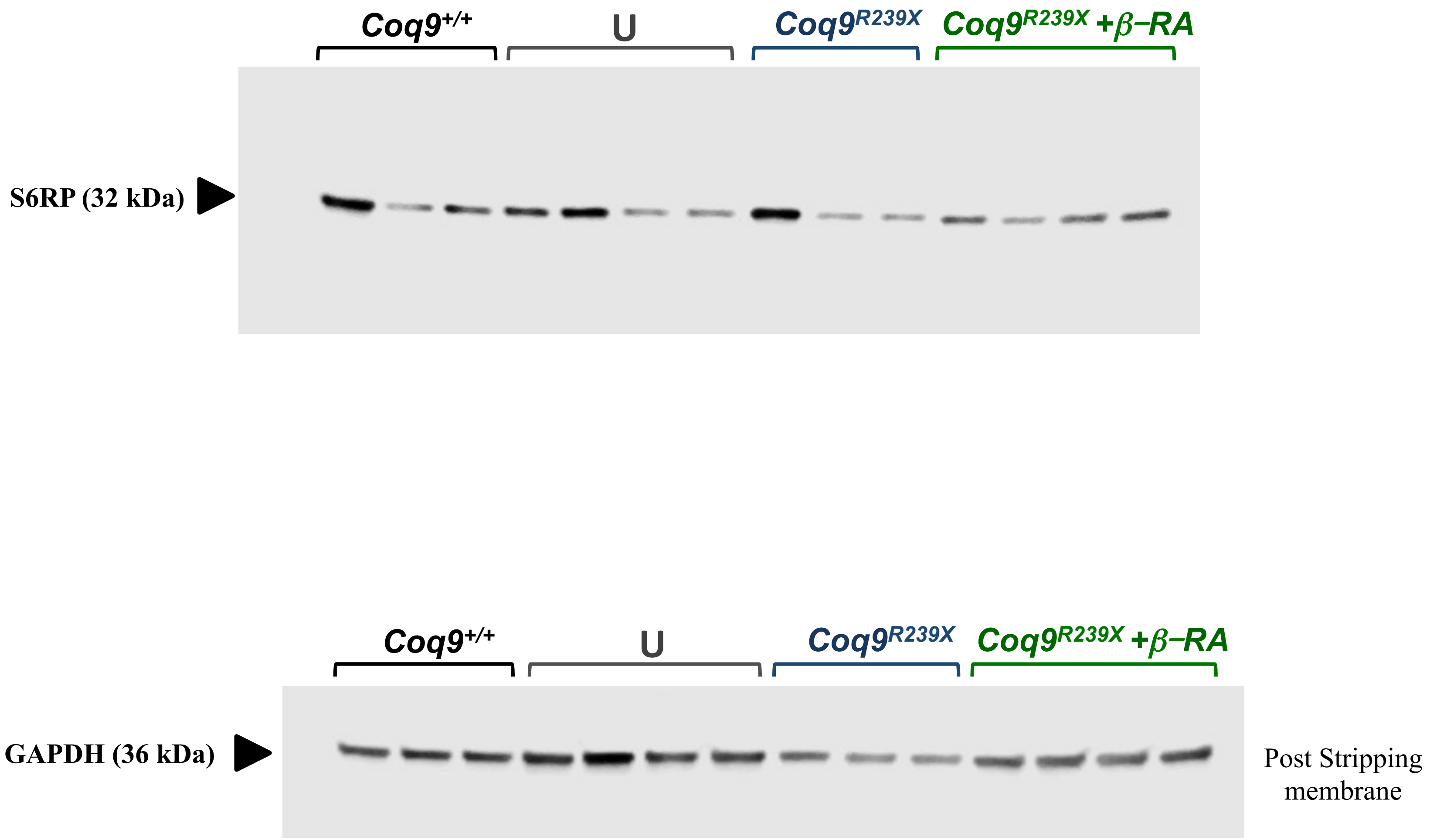

Note: lines 3 and 8-14 are represented in Figure S3B in the main text.

U=Unrelated to this study
