# Supplementary material for: β‐RA reduces DMQ/CoQ ratio and rescues the encephalopathic phenotype in Coq9 R239X mice
Source: EMBO Mol Med. 2018 Nov 27;11(1):e9466. doi: 10.15252/emmm.201809466 (PMC6328940; doi:10.15252/emmm.201809466)
Supplement: Supplementary file 6 — Source Data for Appendix [file EMMM-11-e9466-s008.zip › EMM-2018-0946-Appendix_SourceData-/EMM-2018-09466_SD_FigS9.pdf]

Figure S9A. PDSS2 in brain of wild-type and mutant mice with and without treatment.

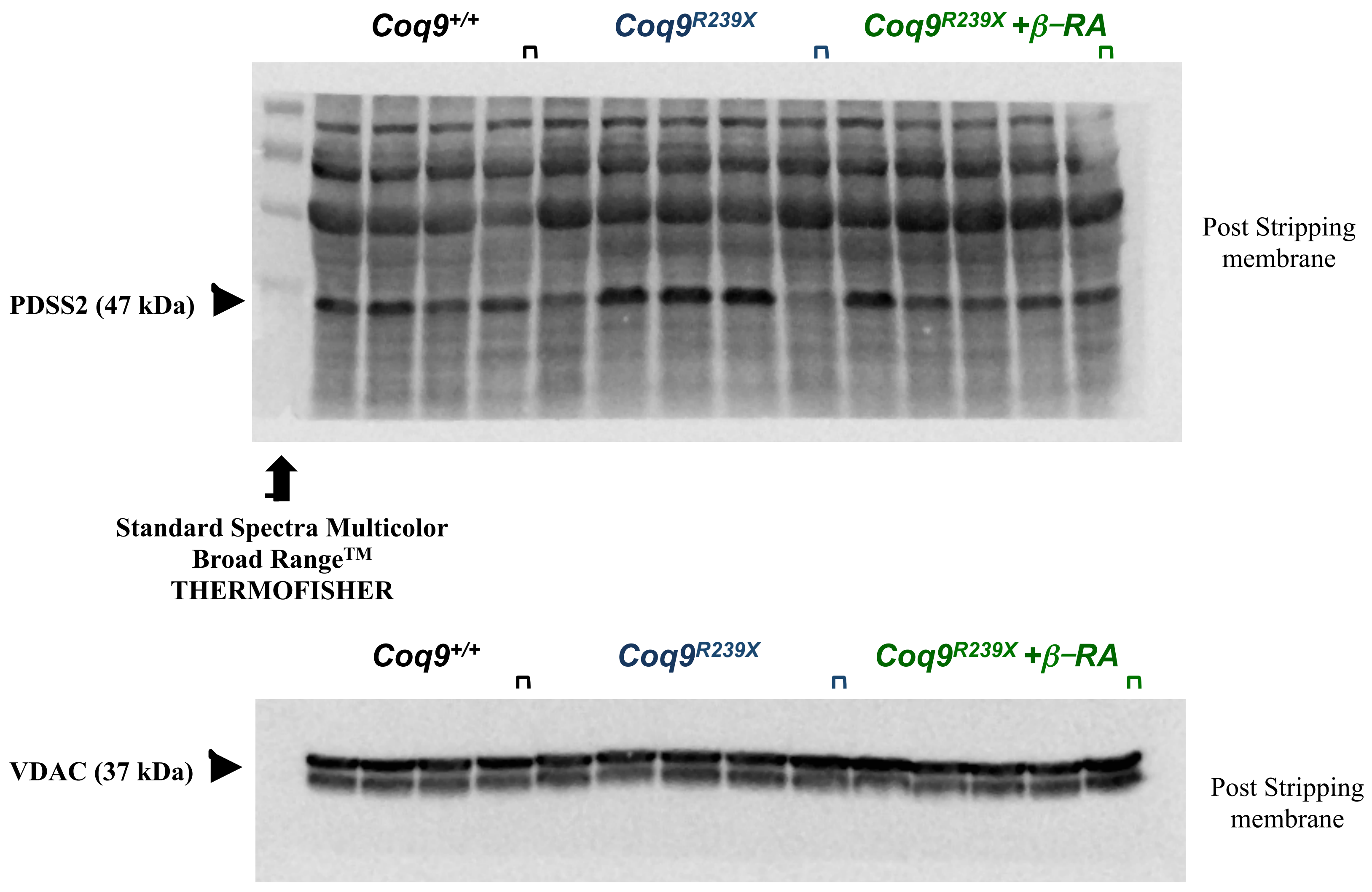

Note: lines 2, 3, 6, 7, 13 and 14 are represented in Figure S9A in the main text.

Figure S9B. COQ2 in brain of wild-type and mutant mice with and without treatment.

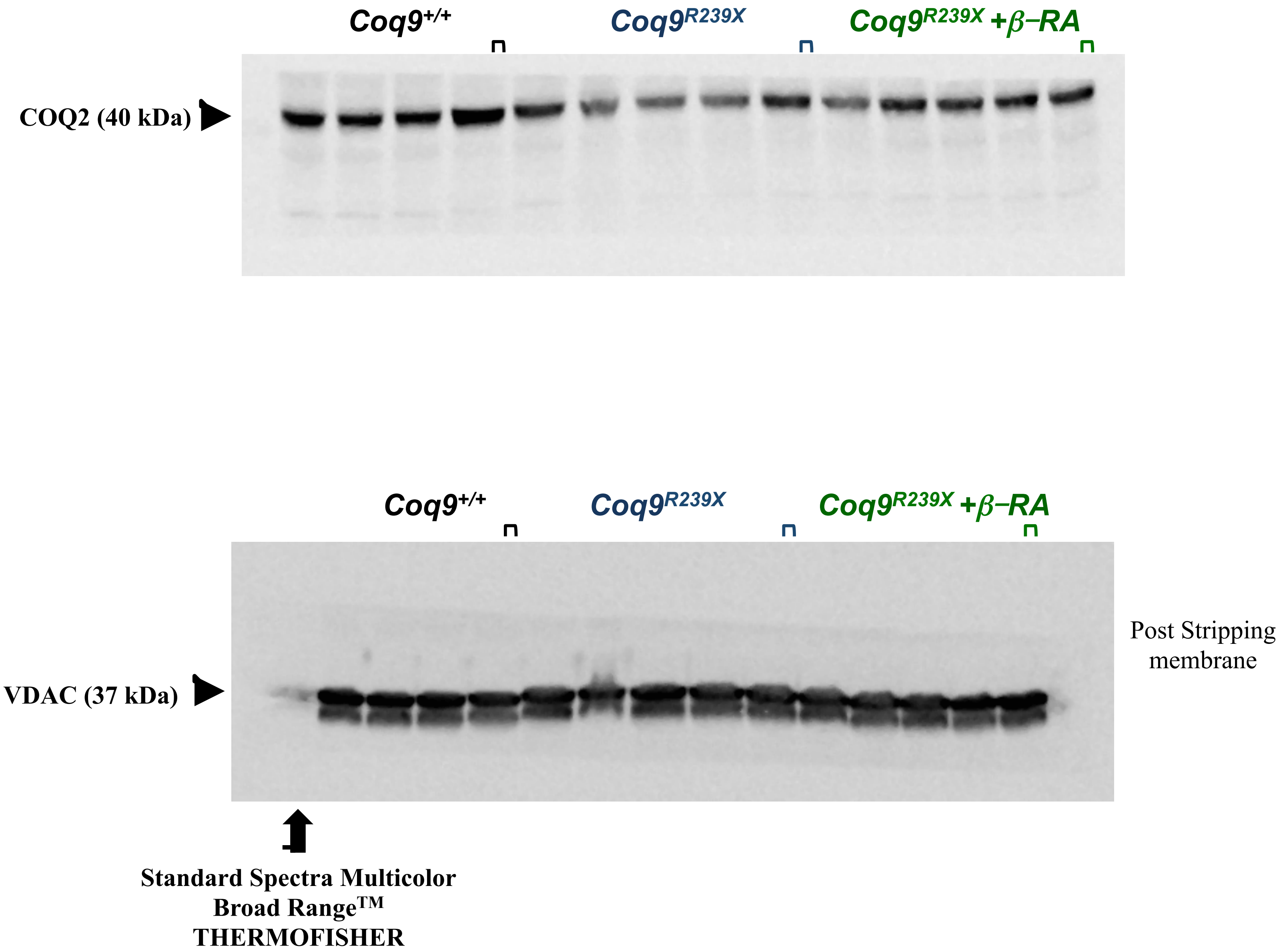

Note: lines 4, 5, 9, 10, 12 and 13 are represented in Figure S9B in the main text.

Figure S9C. PDSS2 in kidney of wild-type and mutant mice with and without treatment.

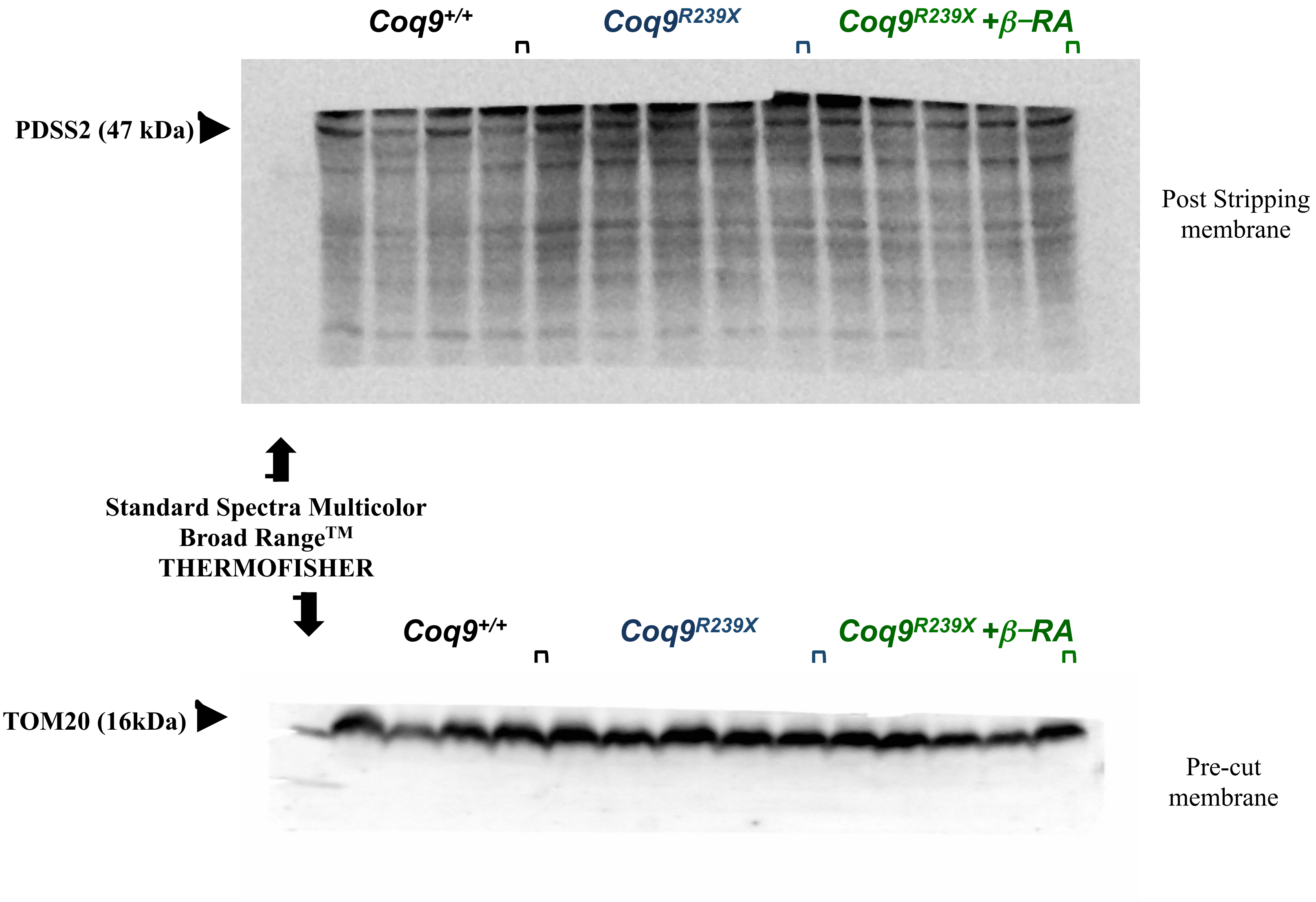

Note: lines 4, 5, 8, 9, 6, 13 and 14 are represented in Figure S9C in the main text.

Figure S9D. COQ2 in kidney of wild-type and mutant mice with and without treatment.

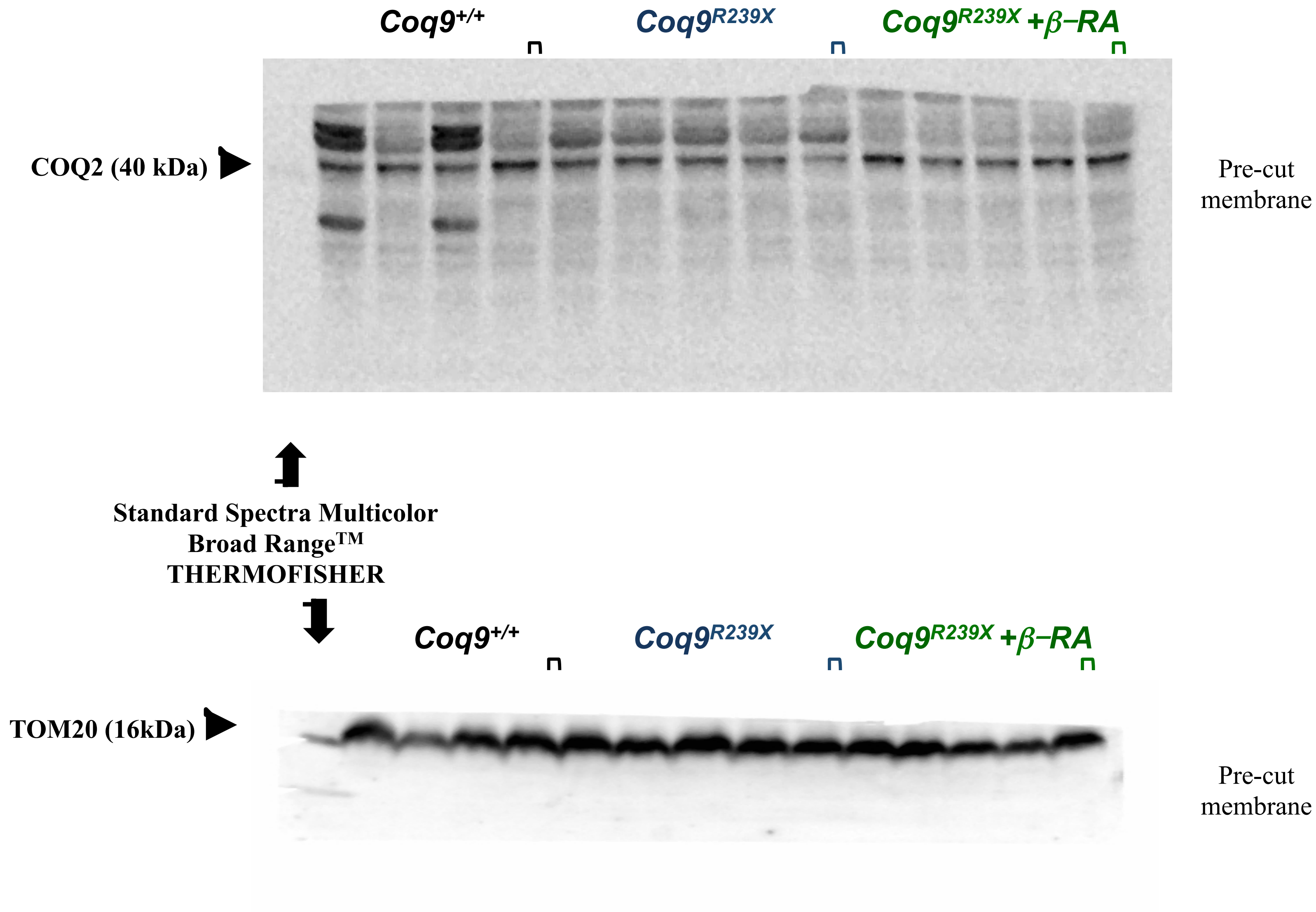

Note: lines 2, 3, 6, 7, 6, 11 and 12 are represented in Figure S9D in the main text.

Figure S9E. PDSS2 in heart of wild-type and mutant mice with and without treatment.

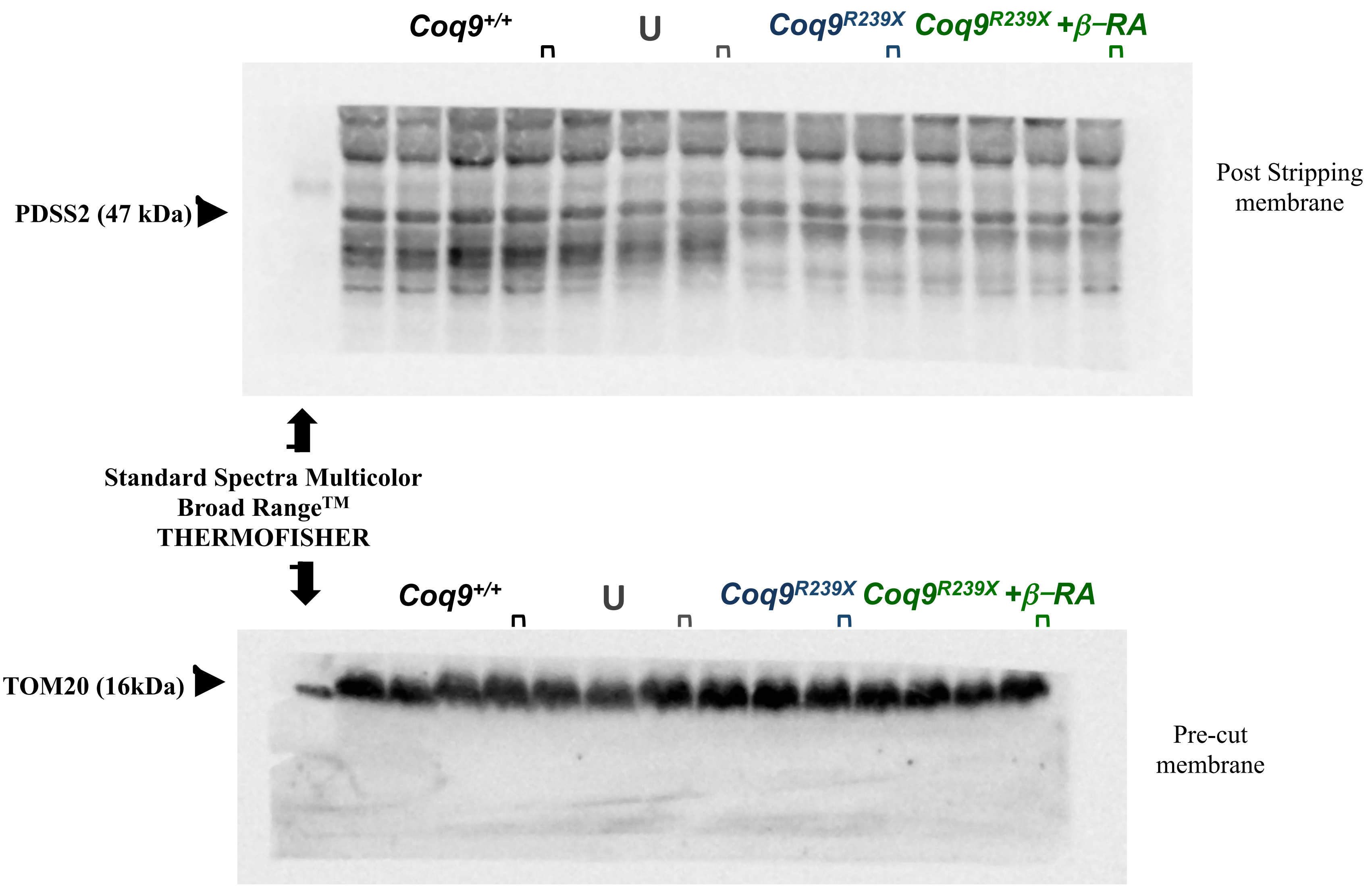

Note: lines 3, 4, 9, 10, 12 and 13 are represented in Figure S9E in the main text.

U=Unrelated to this study

Figure S9F. COQ2 in heart of wild-type and mutant mice with and without treatment.

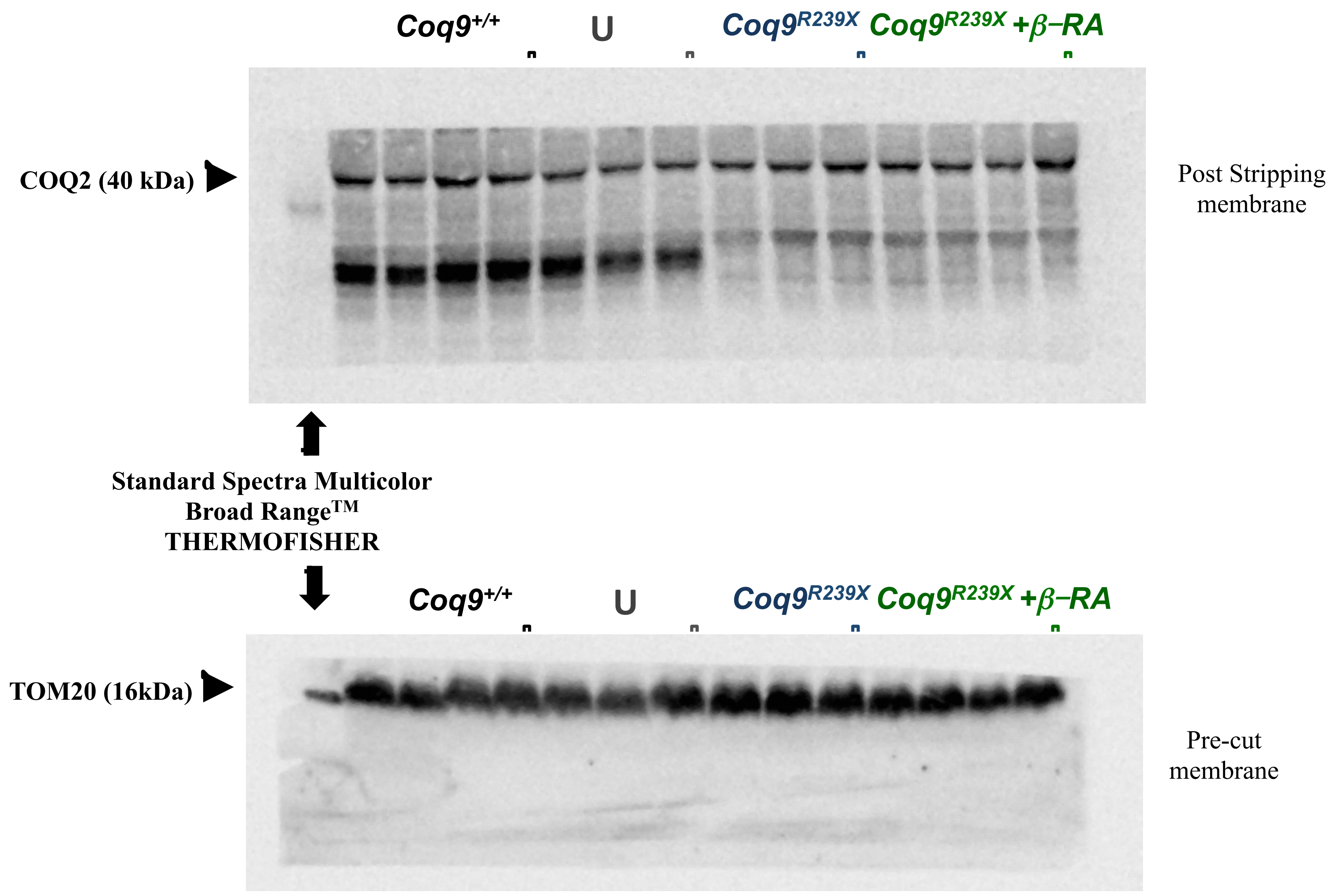

Note: lines 3, 4, 9, 10, 12 and 13 are represented in Figure S9E in the main text.

U=Unrelated to this study
