# Supplementary material for: β‐RA reduces DMQ/CoQ ratio and rescues the encephalopathic phenotype in Coq9 R239X mice
Source: EMBO Mol Med. 2018 Nov 27;11(1):e9466. doi: 10.15252/emmm.201809466 (PMC6328940; doi:10.15252/emmm.201809466)

Figure 4C. SQOR in cerebral mitochondria of wild-type and mutant mice with and without treatment.

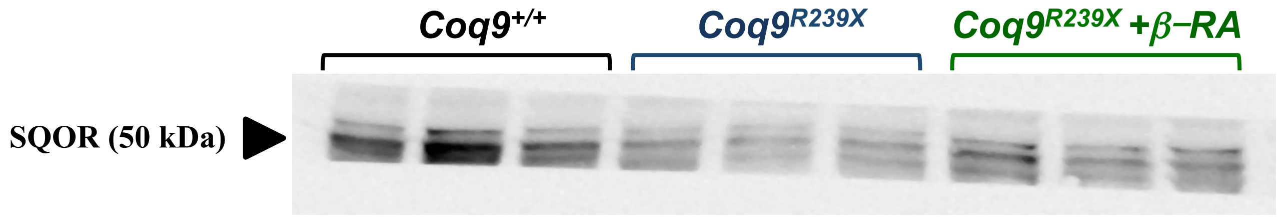

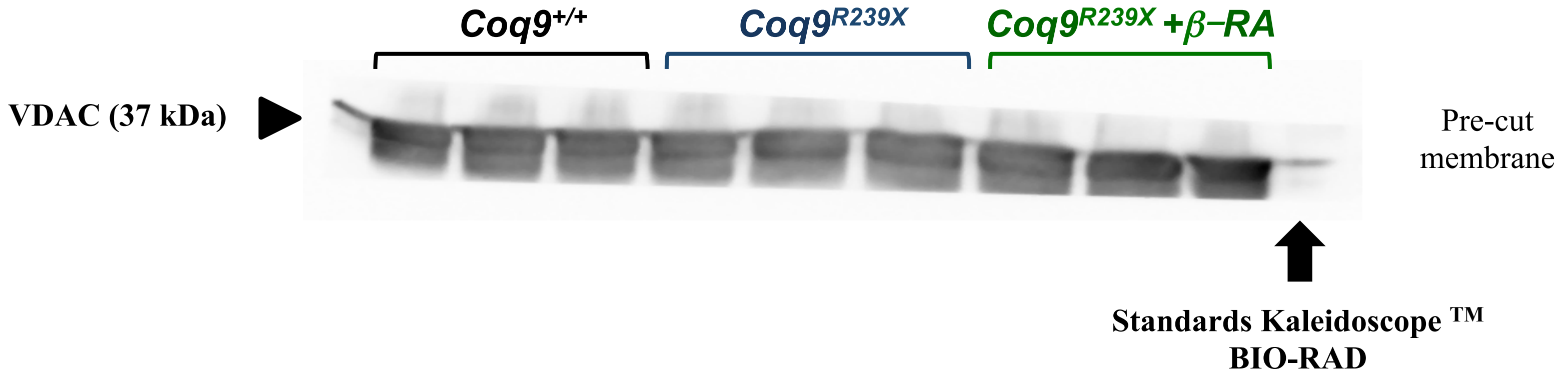

Figure 4H. SQOR in kidney of wild-type and mutant mice with and without treatment.

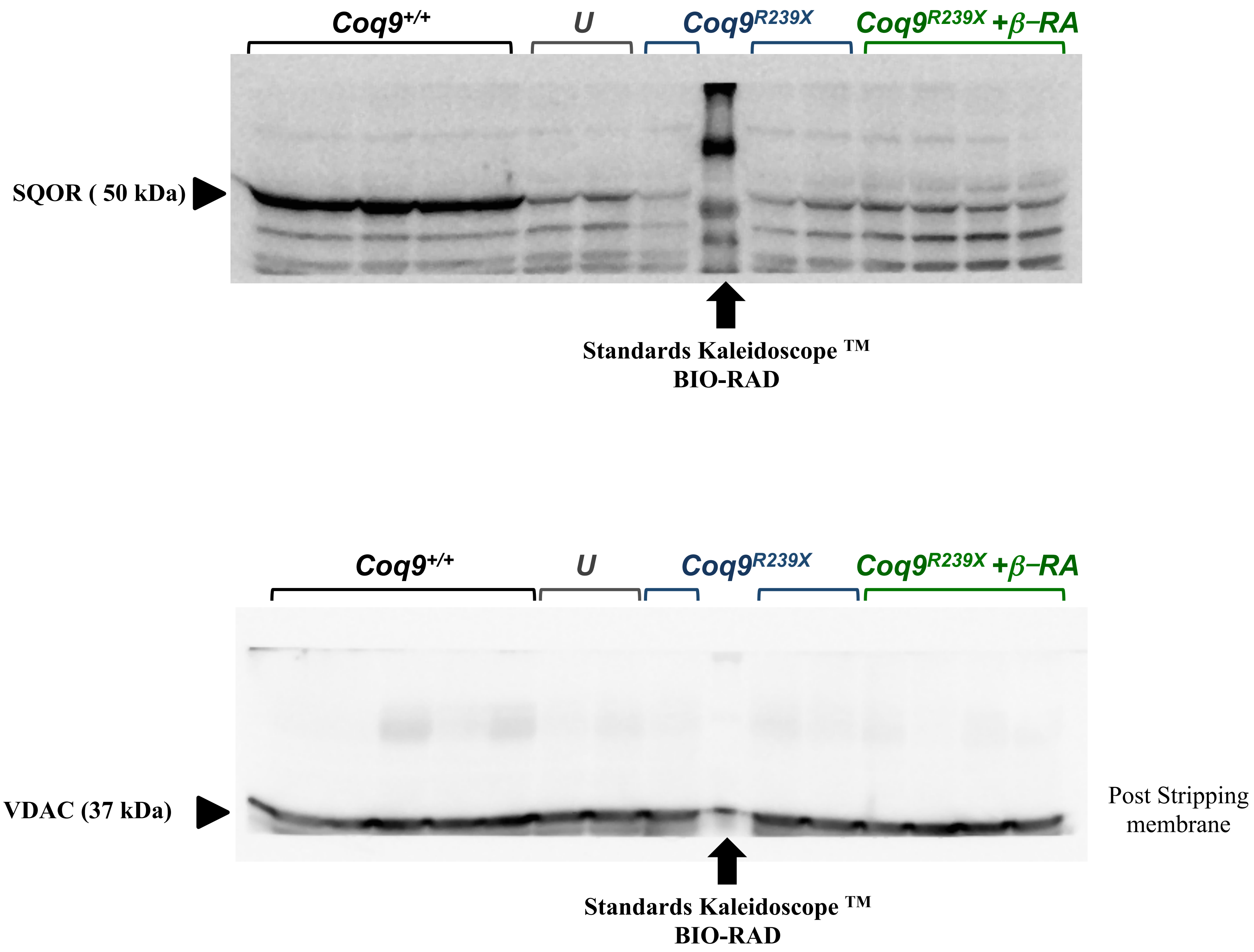

Note: lines 3, 4, 10, 11, 12 and 13 are represented in Figure 4H in the main text.

U=Unrelated to this study

Figure 4O. SQOR in heart of wild-type and mutant mice with and without treatment.

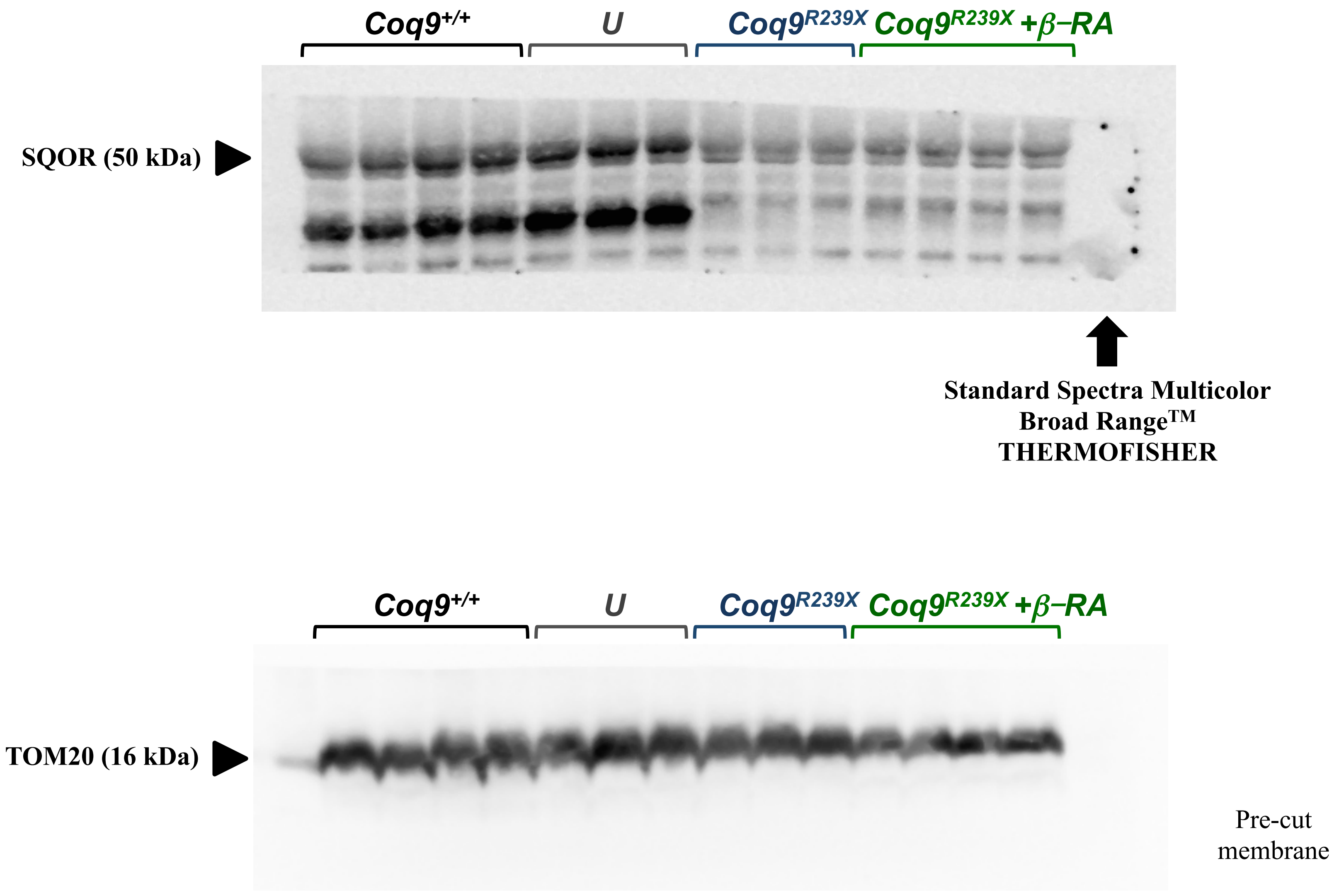

Note: lines 1-4 and 8-14 are represented in Figure 4O in the main text.

U=Unrelated to this study

Figure 4D. Blue native of CIII in brain of wild-type and mutant mice with and without treatment.

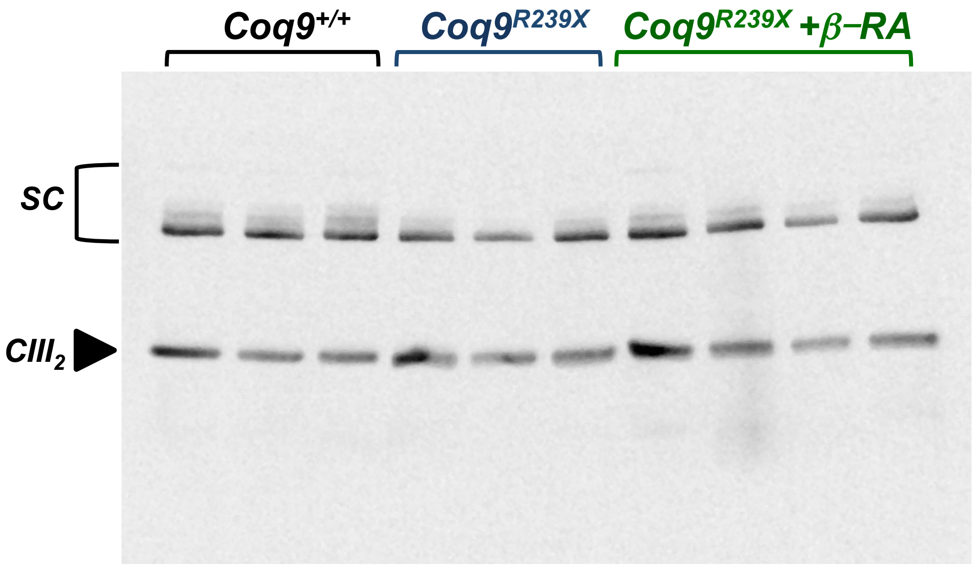

Note: lines 2, 3, 4, 5, 9 y 10 are represented in Figure 4D in the main text.

Figure 4I. Blue native of CIII in kidney of wild-type and mutant mice with and without treatment.

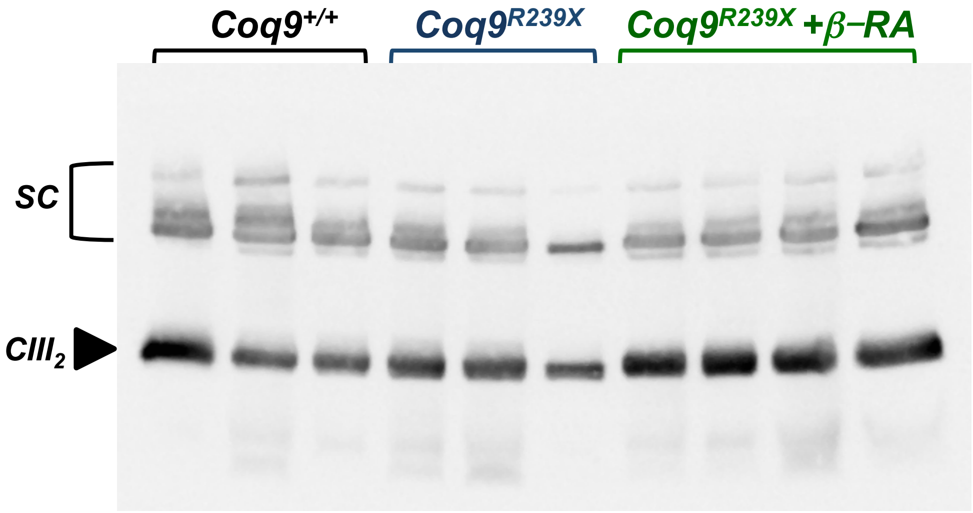

Supplement: Supplementary file 8 — Source Data for Figure 4 [file EMMM-11-e9466-s006.pdf]
