# Supplementary material for: β‐RA reduces DMQ/CoQ ratio and rescues the encephalopathic phenotype in Coq9 R239X mice
Source: EMBO Mol Med. 2018 Nov 27;11(1):e9466. doi: 10.15252/emmm.201809466 (PMC6328940; doi:10.15252/emmm.201809466)
Supplement: Supplementary file 9 — Source Data for Figure 6 [file EMMM-11-e9466-s007.pdf]

Figure 6A. COQ4 in brain of wild-type and mutant mice with and without treatment.

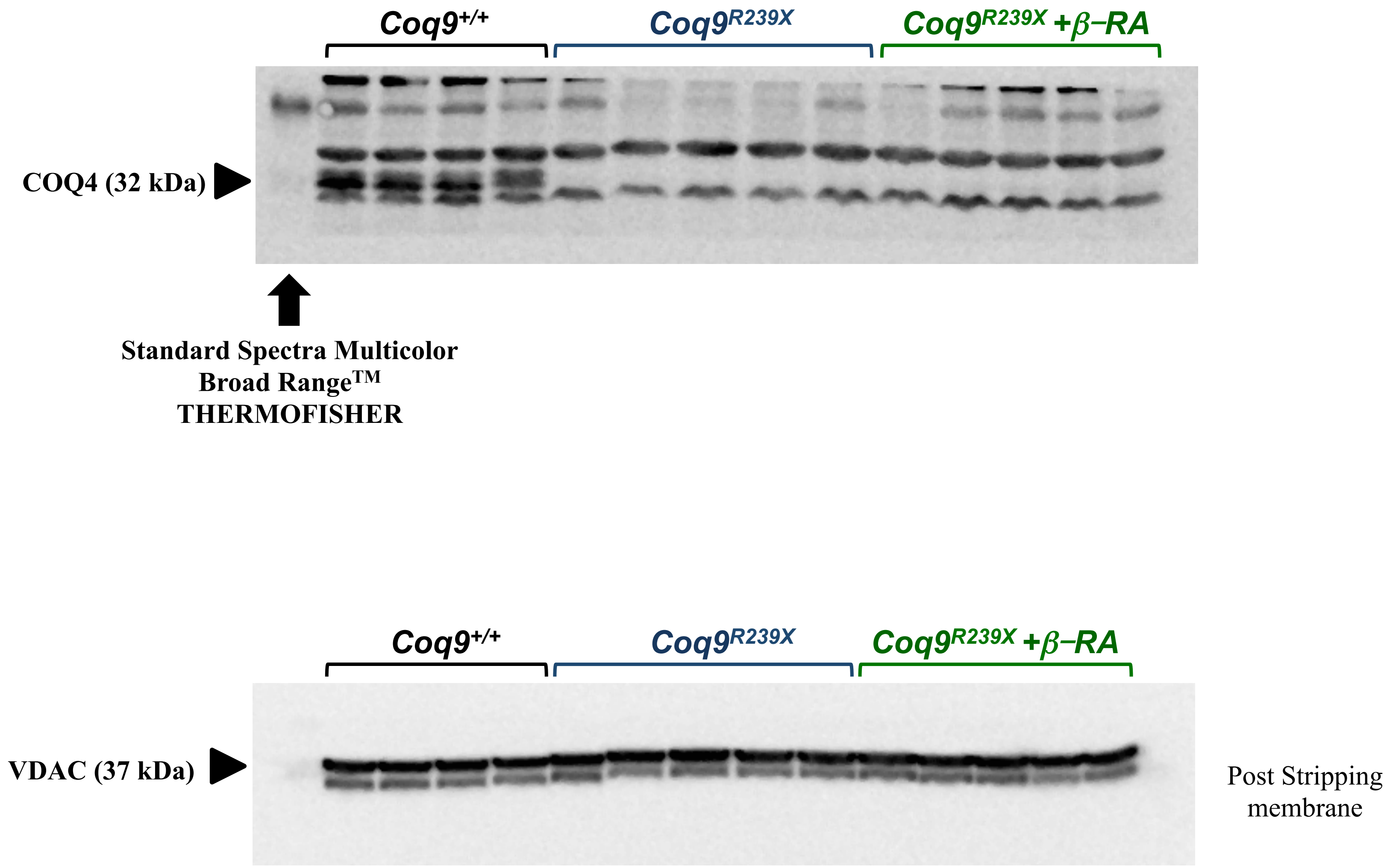

Note: lines 4, 5, 6, 7, 12 and 13 are represented in Figure 6A in the main text.

Figure 6B. COQ5 in brain of wild-type and mutant mice with and without treatment.

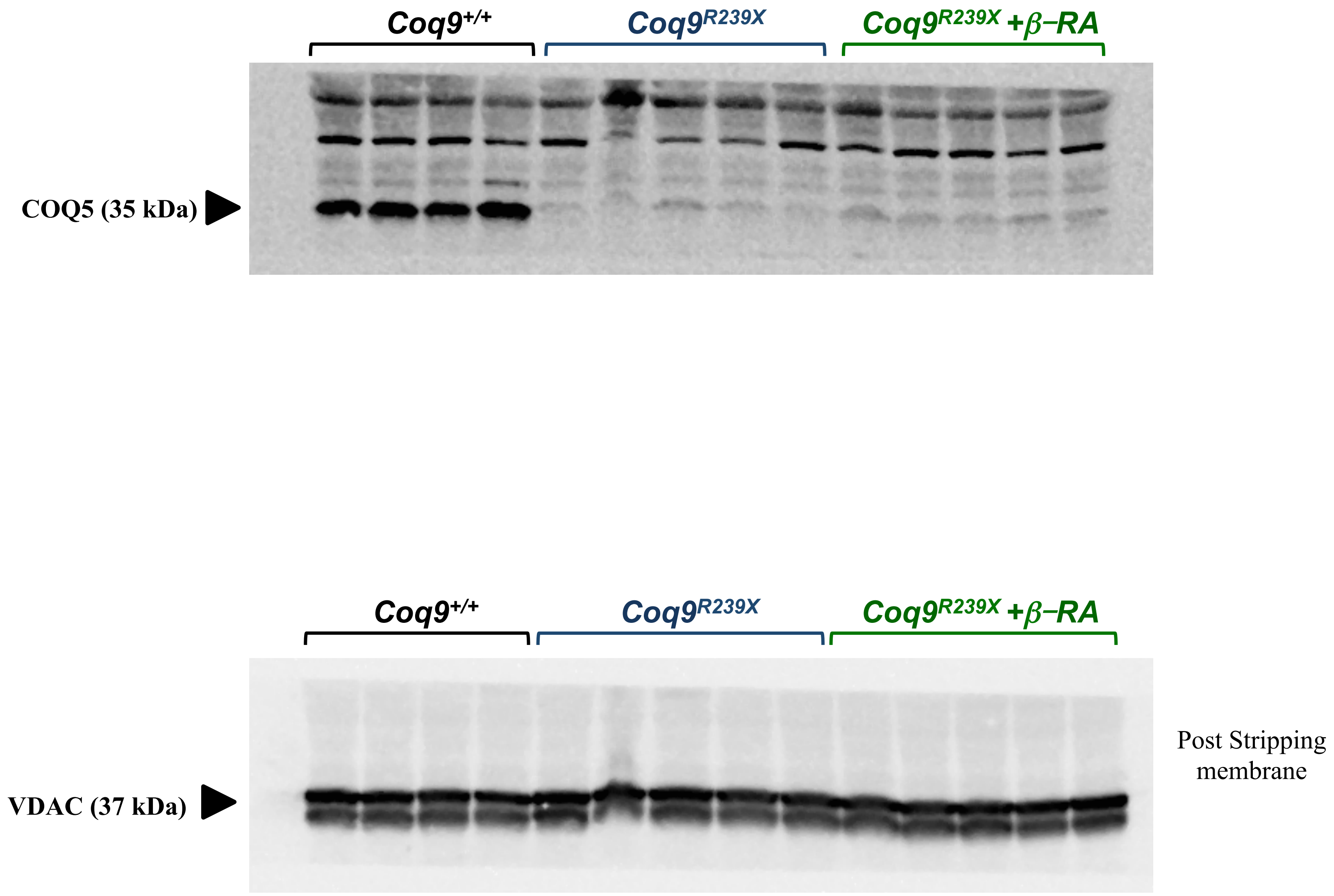

Note: lines 4, 5, 9, 10, 13 and 14 are represented in Figure 6B in the main text.

Figure 6C. COQ6 of brain of wild-type and mutant mice with and without treatment.

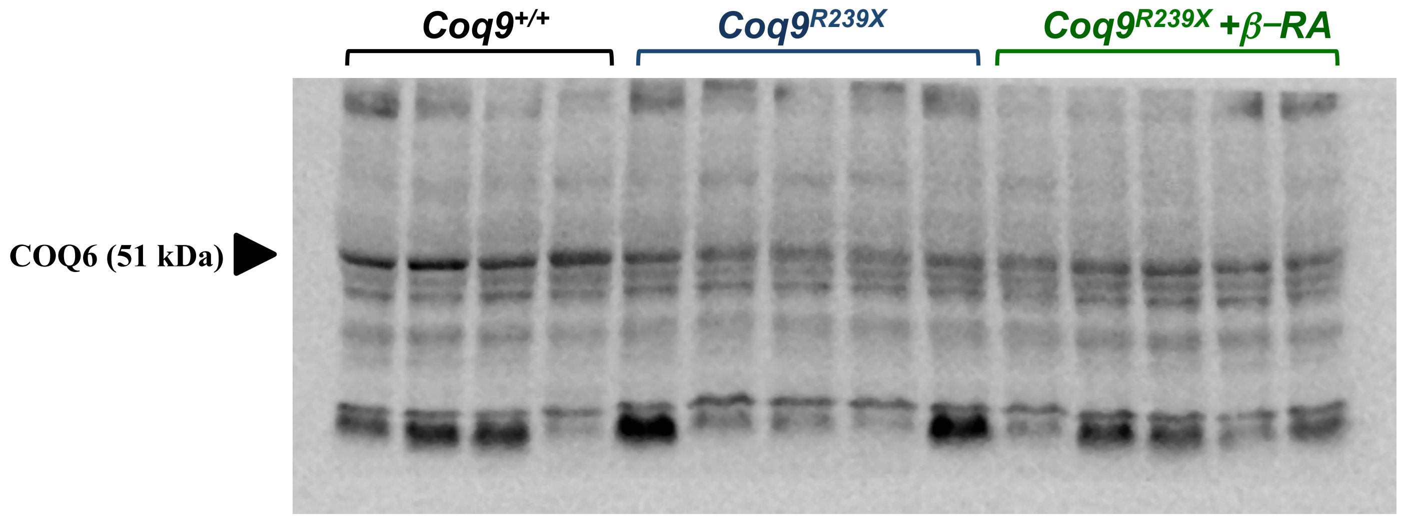

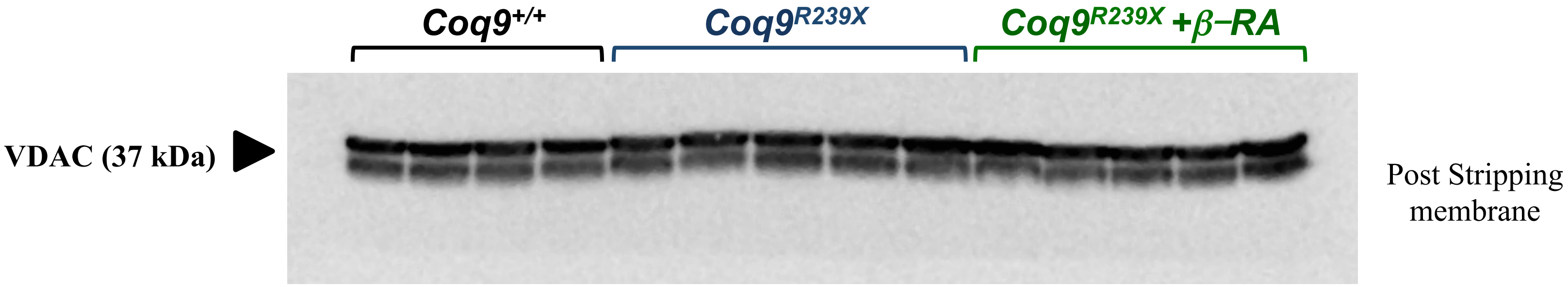

Note: lines 4, 5, 7, 8, 13 and 14 are represented in Figure 6C in the main text.

Figure 6D. COQ7 of brain of wild-type and mutant mice with and without treatment.

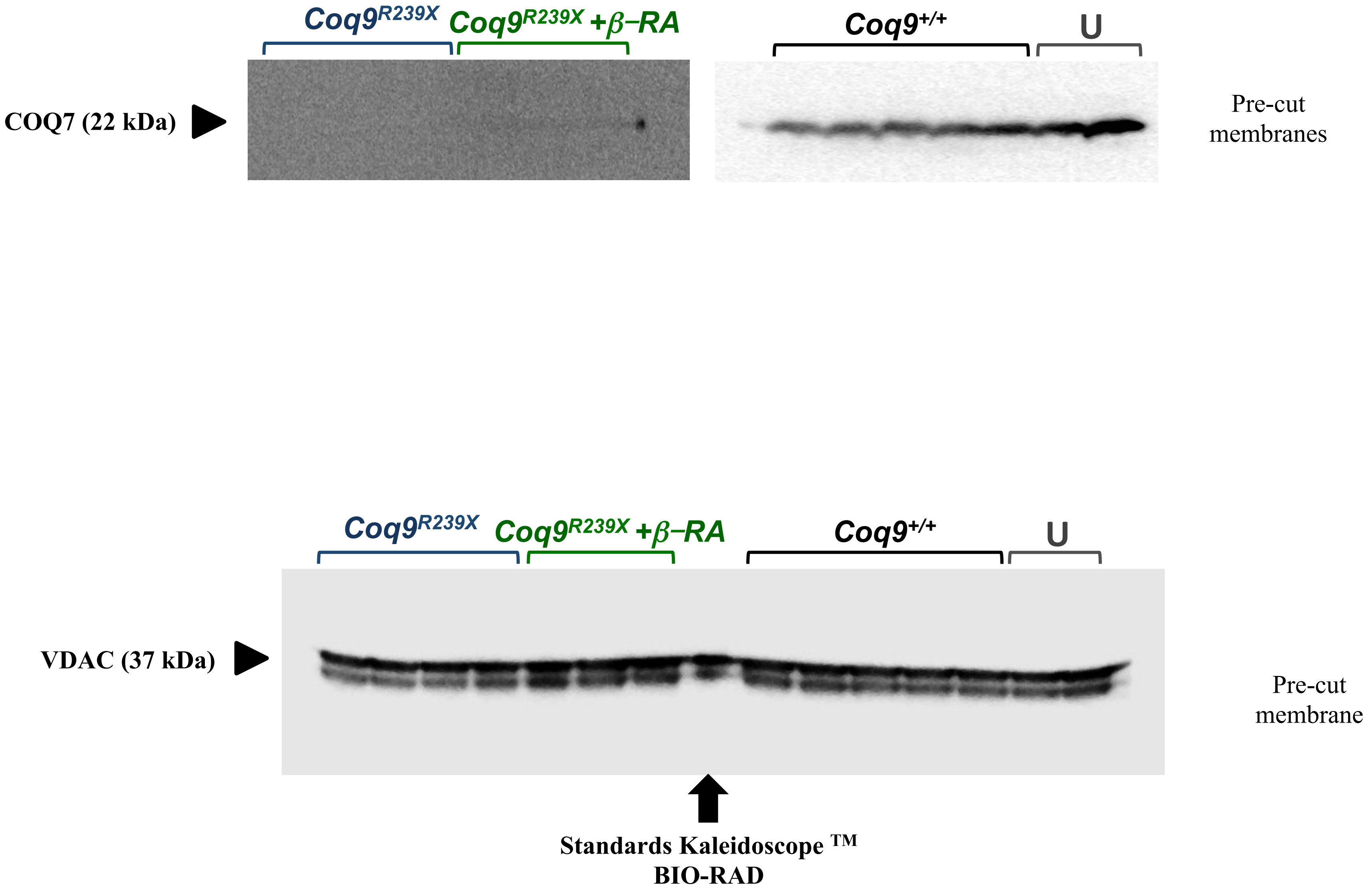

Note: lines 1, 2, 5, 6, 12 and 13 are represented in Figure 6D in the main text.

Figure 6E. COQ8A in brain of wild-type and mutant mice with and without treatment.

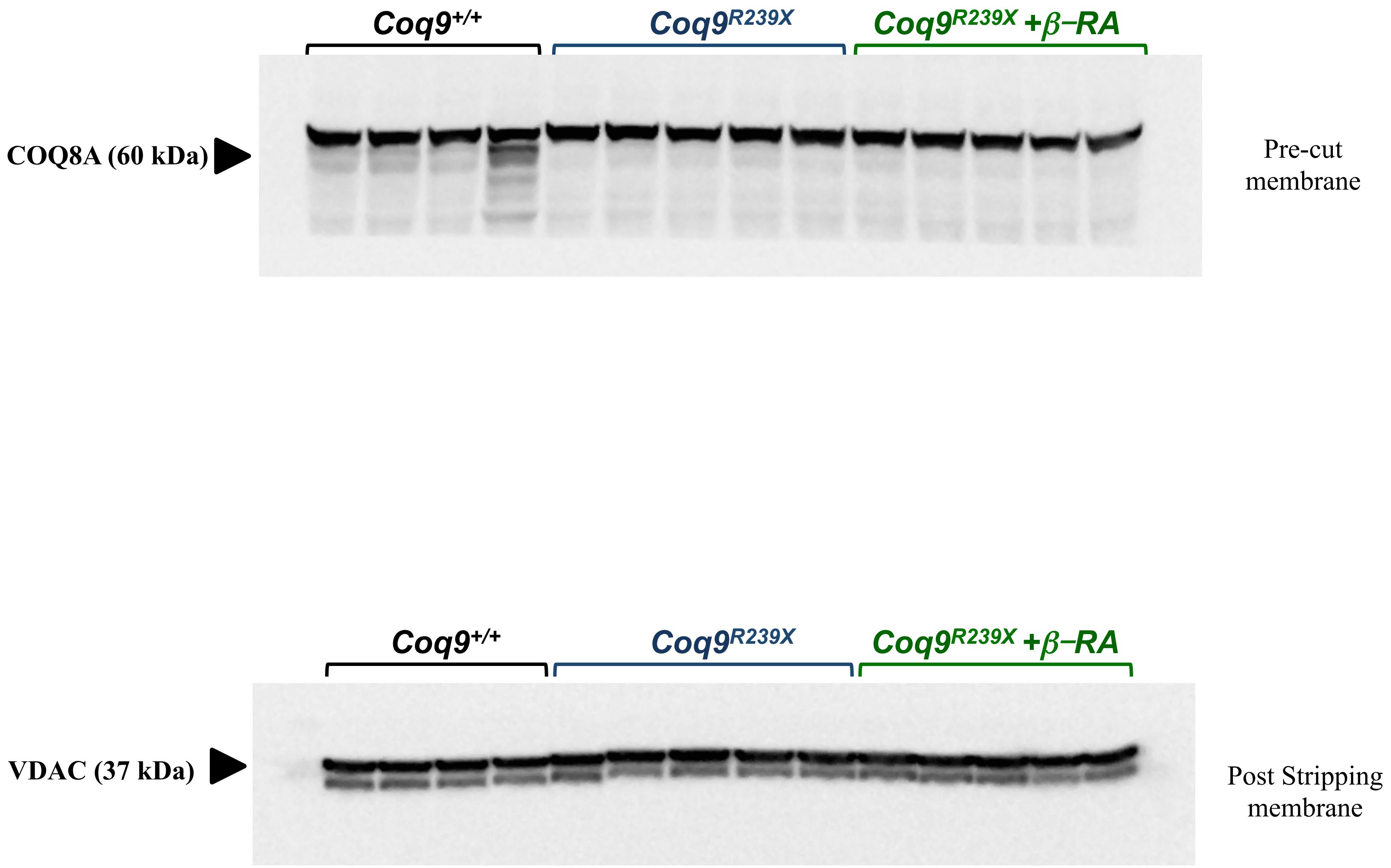

Note: lines 2, 3, 5, 6, 11 and 12 are represented in Figure 6E in the main text.

Figure 6F. COQ4 in kidney of wild-type and mutant mice with and without treatment.

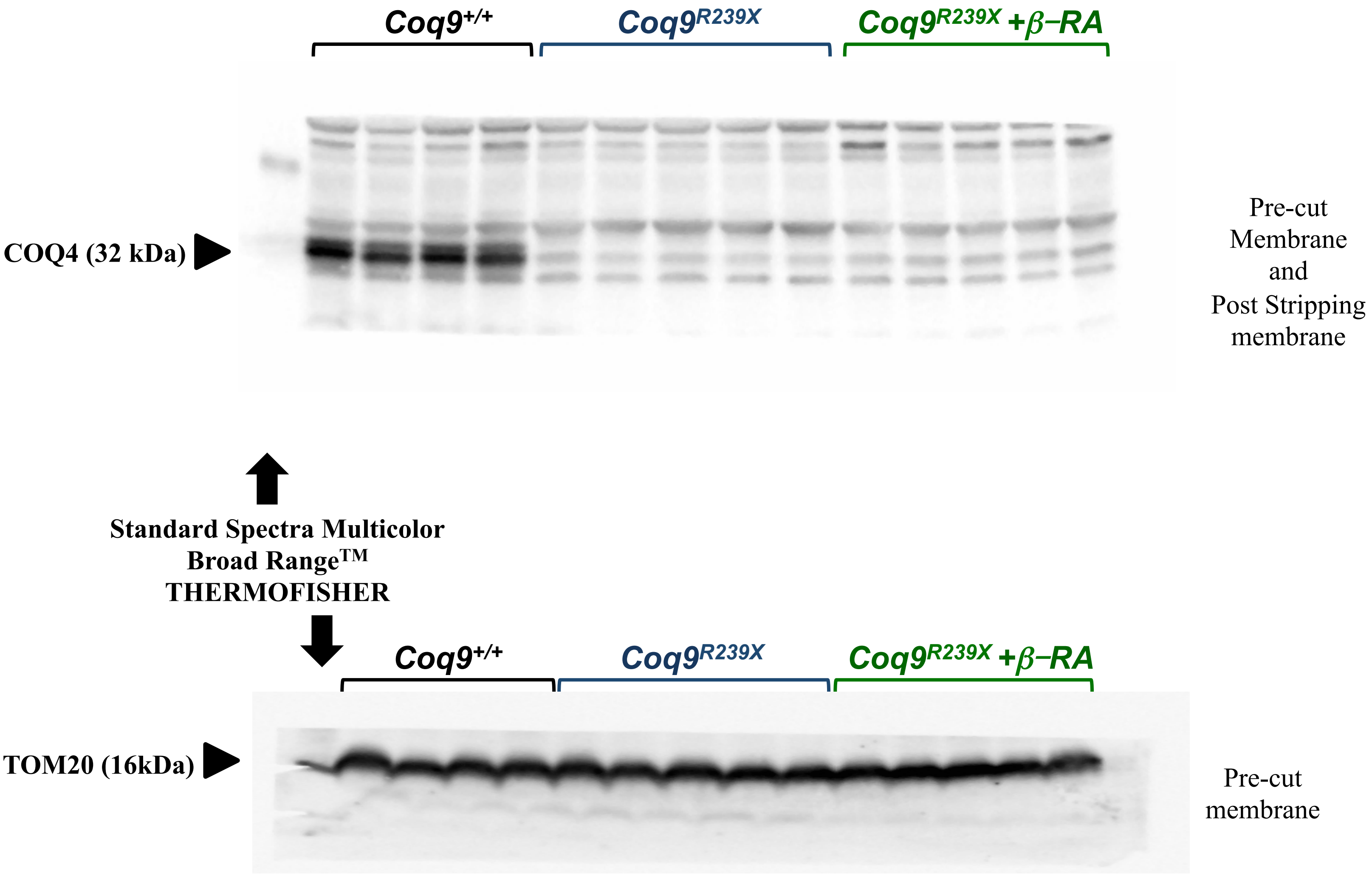

Note: lines 4, 5, 7, 8, 14 and 15 are represented in Figure 6G in the main text.

Figure 6G. COQ5 in kidney of wild-type and mutant mice with and without treatment.

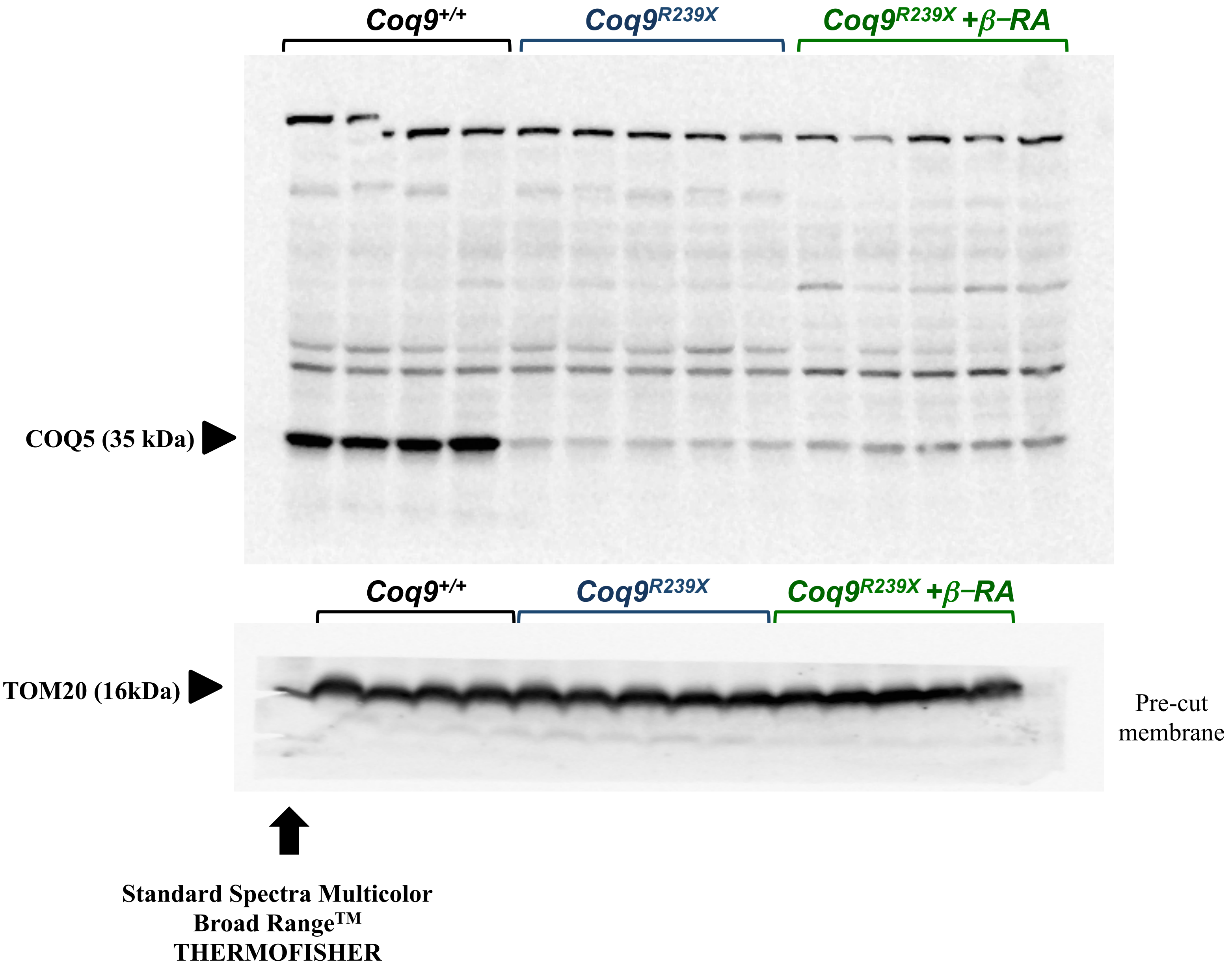

Note: lines 4, 5, 7, 8, 14 and 15 are represented in Figure 6H in the main text.

Figure 6H. COQ6 in kidney of wild-type and mutant mice with and without treatment.

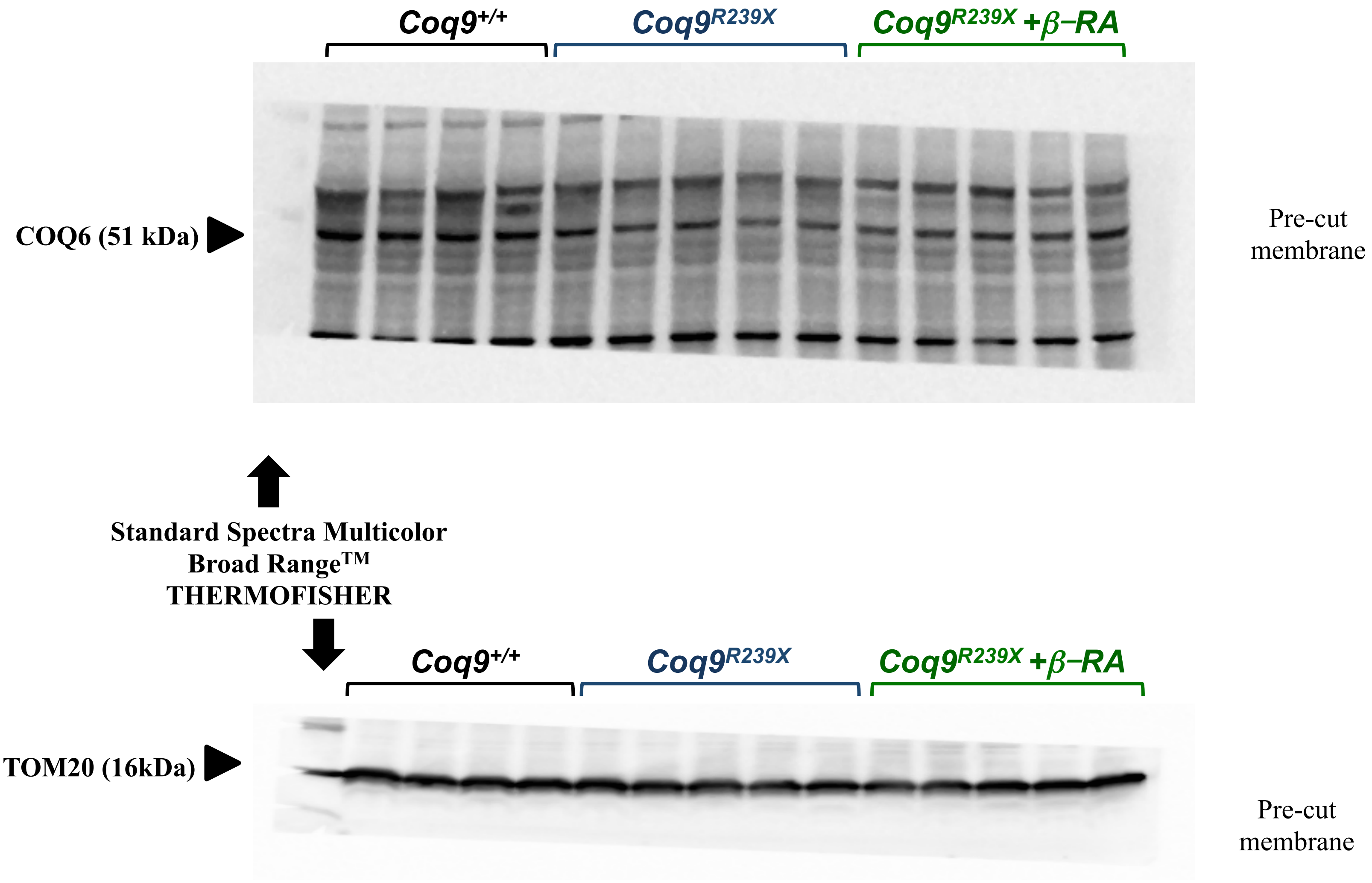

Note: lines 4, 5, 9, 10, 13 and 14 are represented in Figure 6I in the main text.

Figure 6I. COQ7 in kidney of wild-type and mutant mice with and without treatment.

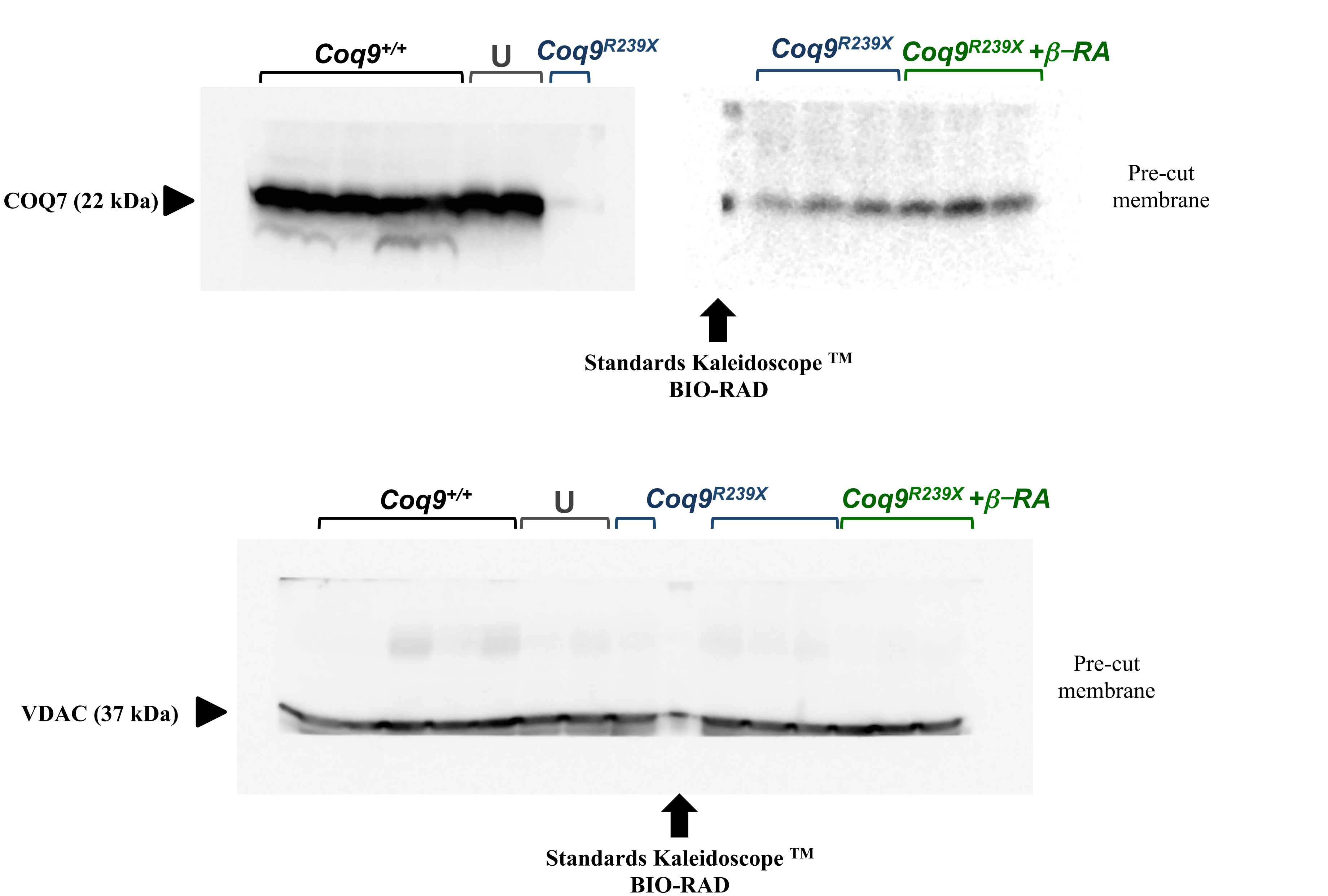

Note: lines 3,4, 10, 11, 13 and 14 are represented in Figure 6J in the main text.

U=Unrelated to this study

Figure 6J. COQ8A of wild-type and mutant mice with and without treatment.

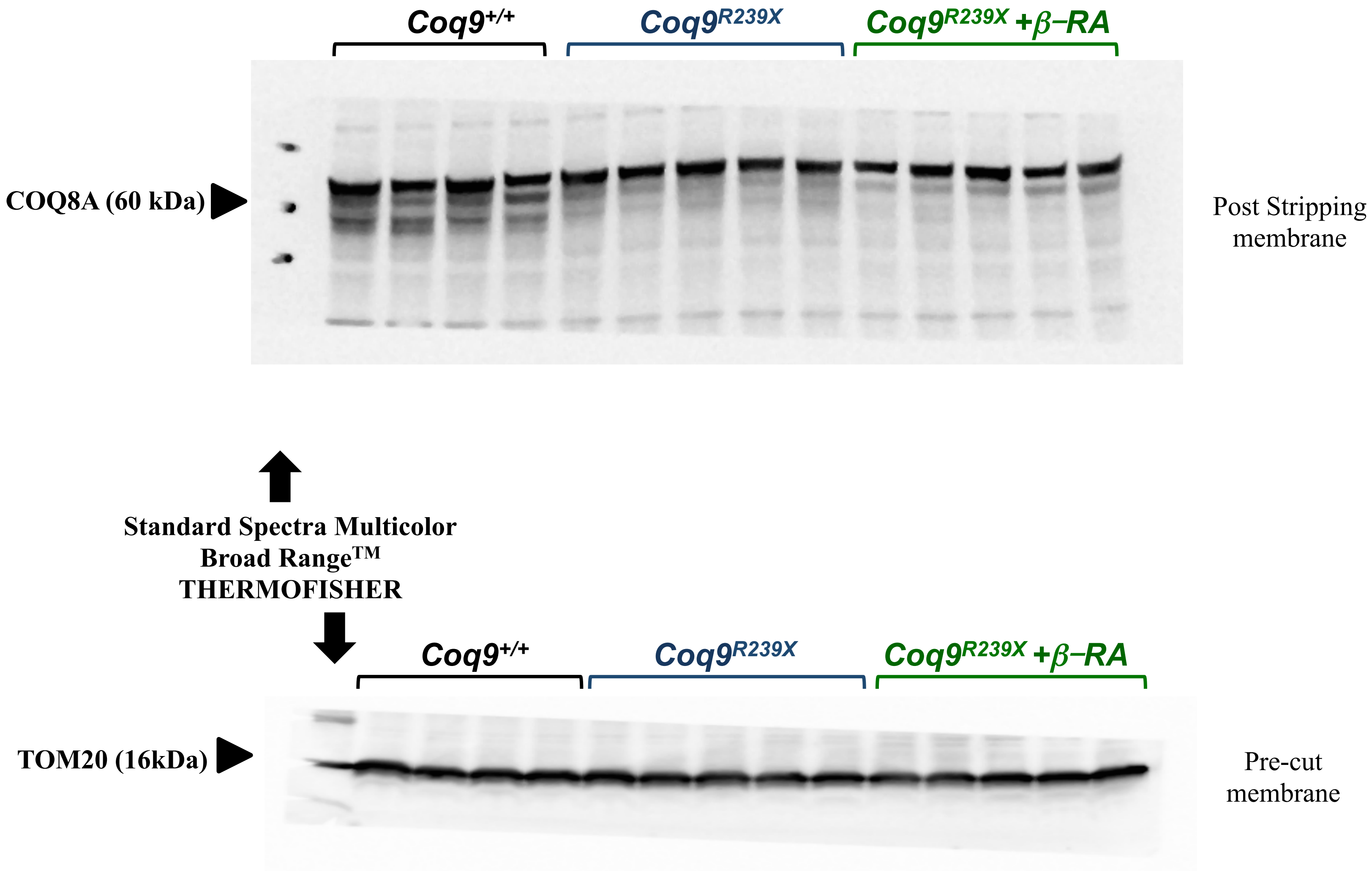

Note: lines 4,5, 7, 8, 12 and 13 are represented in Figure 6K in the main text.

Figure 6K. COQ4 in heart of wild-type and mutant mice with and without treatment.

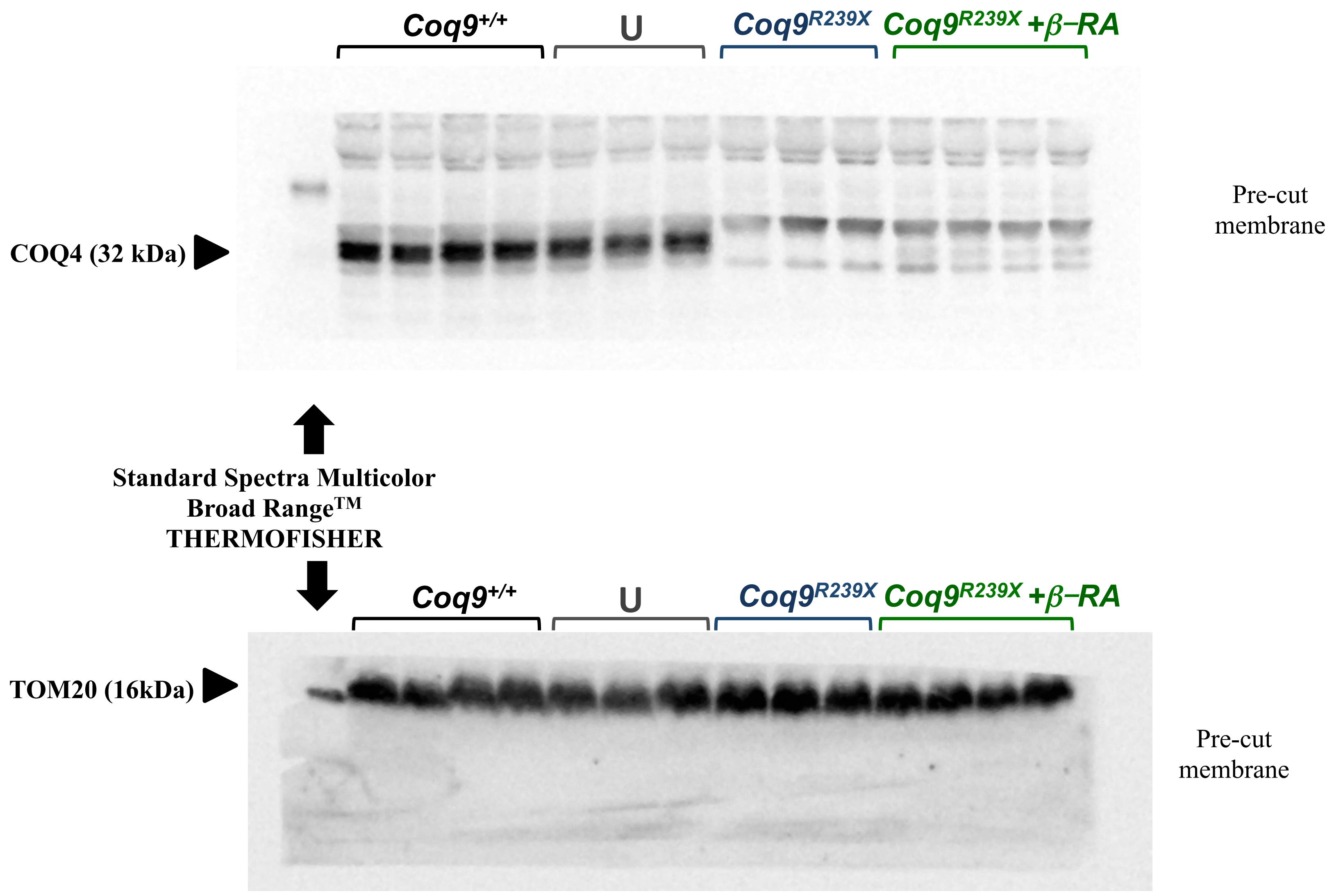

Note: lines 1,2 and 9-12 are represented in Figure 6L in the main text.

U=Unrelated to this study

Figure 6L. COQ5 in heart of wild-type and mutant mice with and without treatment.

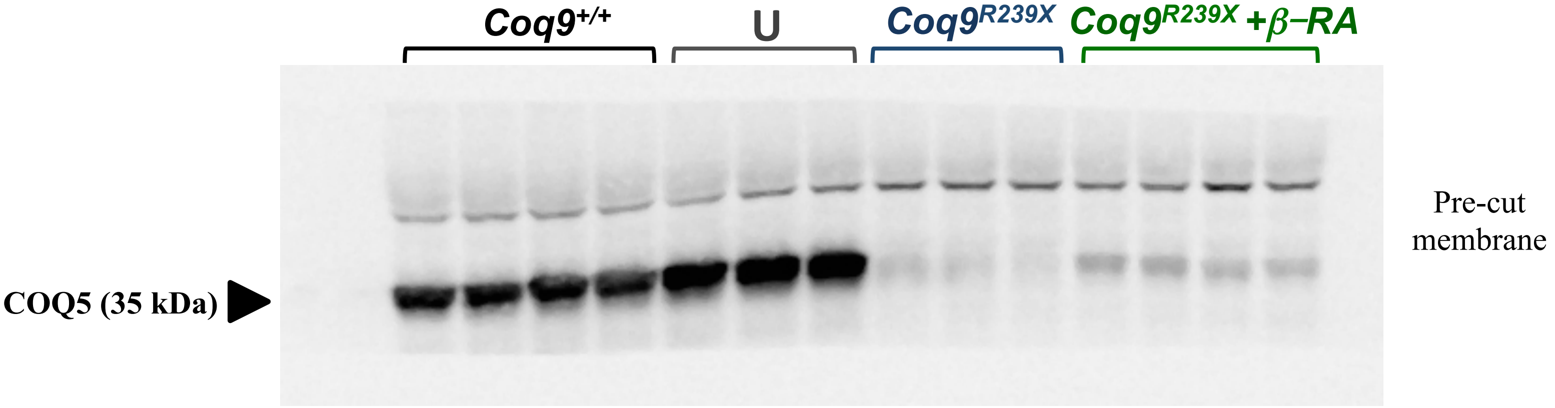

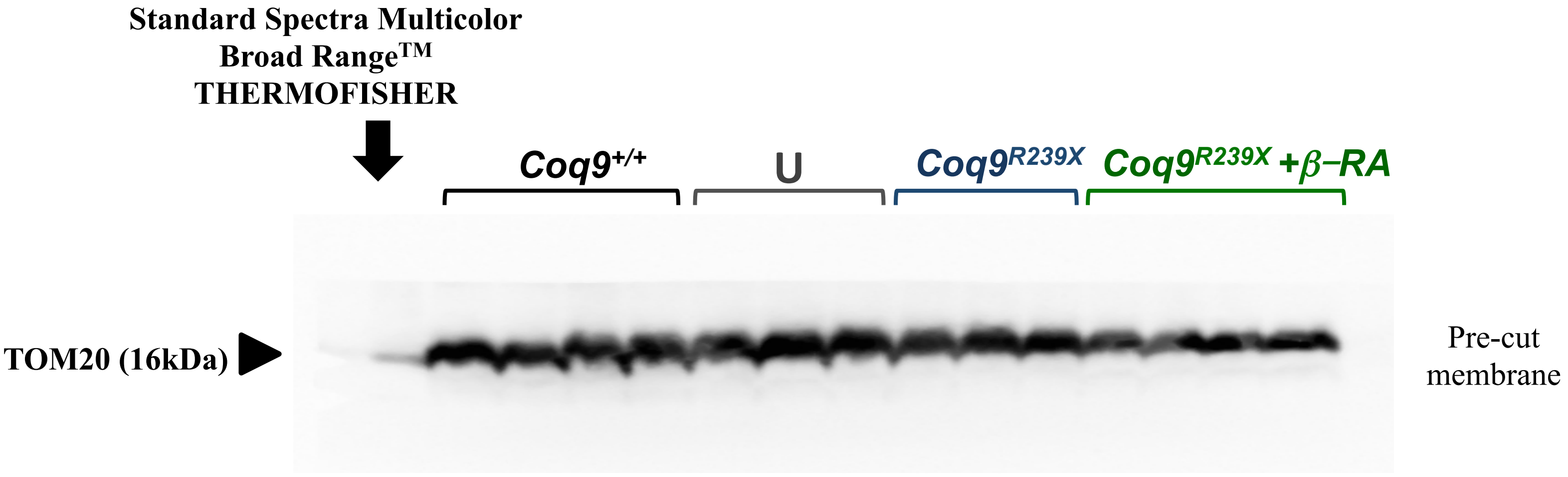

Note: lines 4, 5, 10, 11, 12 and 13 are represented in Figure 6M in the main text.

U=Unrelated to this study

Figure 6M. COQ6 in heart of wild-type and mutant mice with and without treatment.

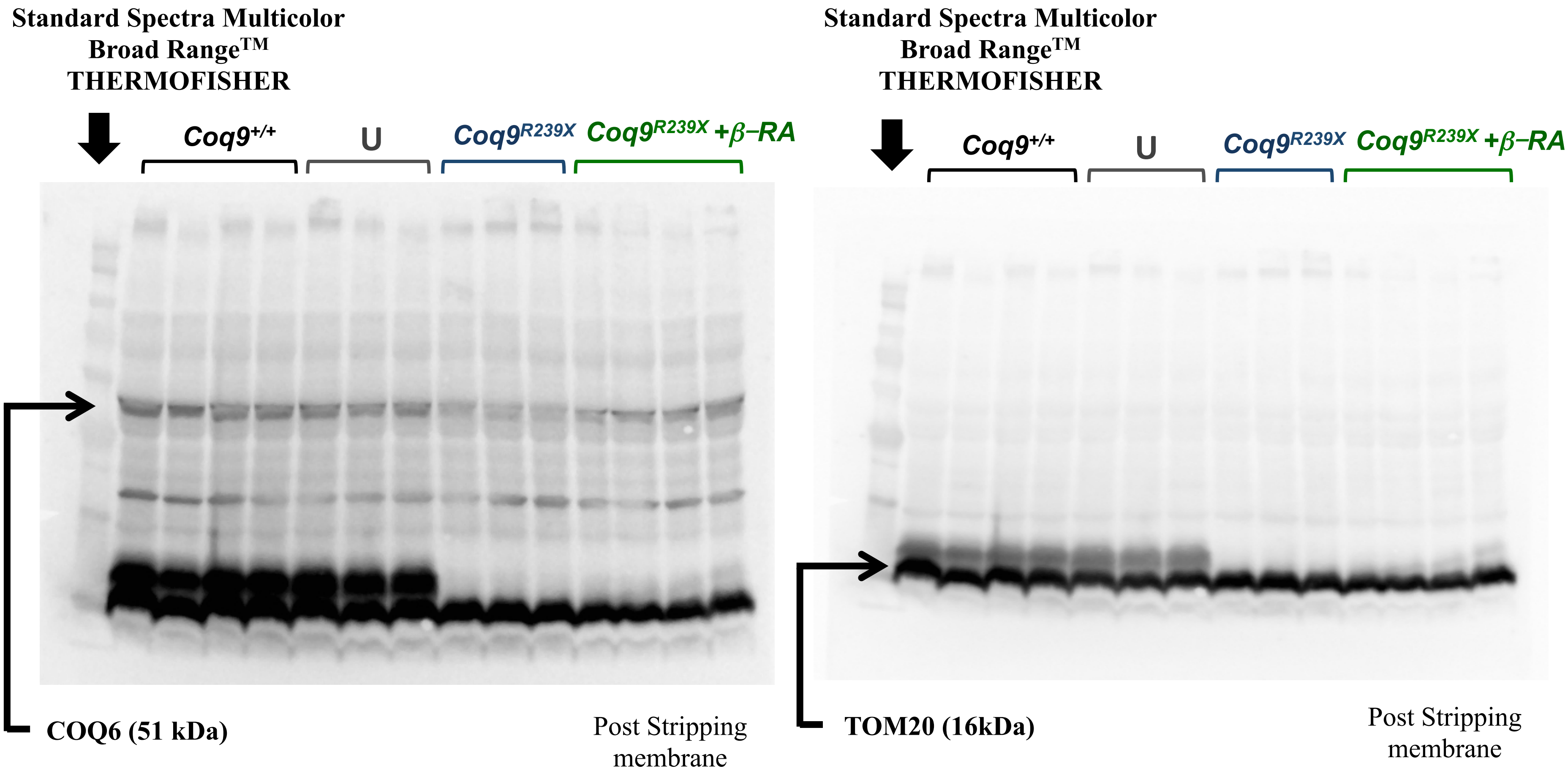

Note: lines 3, 4, 9, 10, 13 and 14 are represented in Figure 6N in the main text.

U=Unrelated to this study

Figure 6N. COQ7 in heart of wild-type and mutant mice with and without treatment.

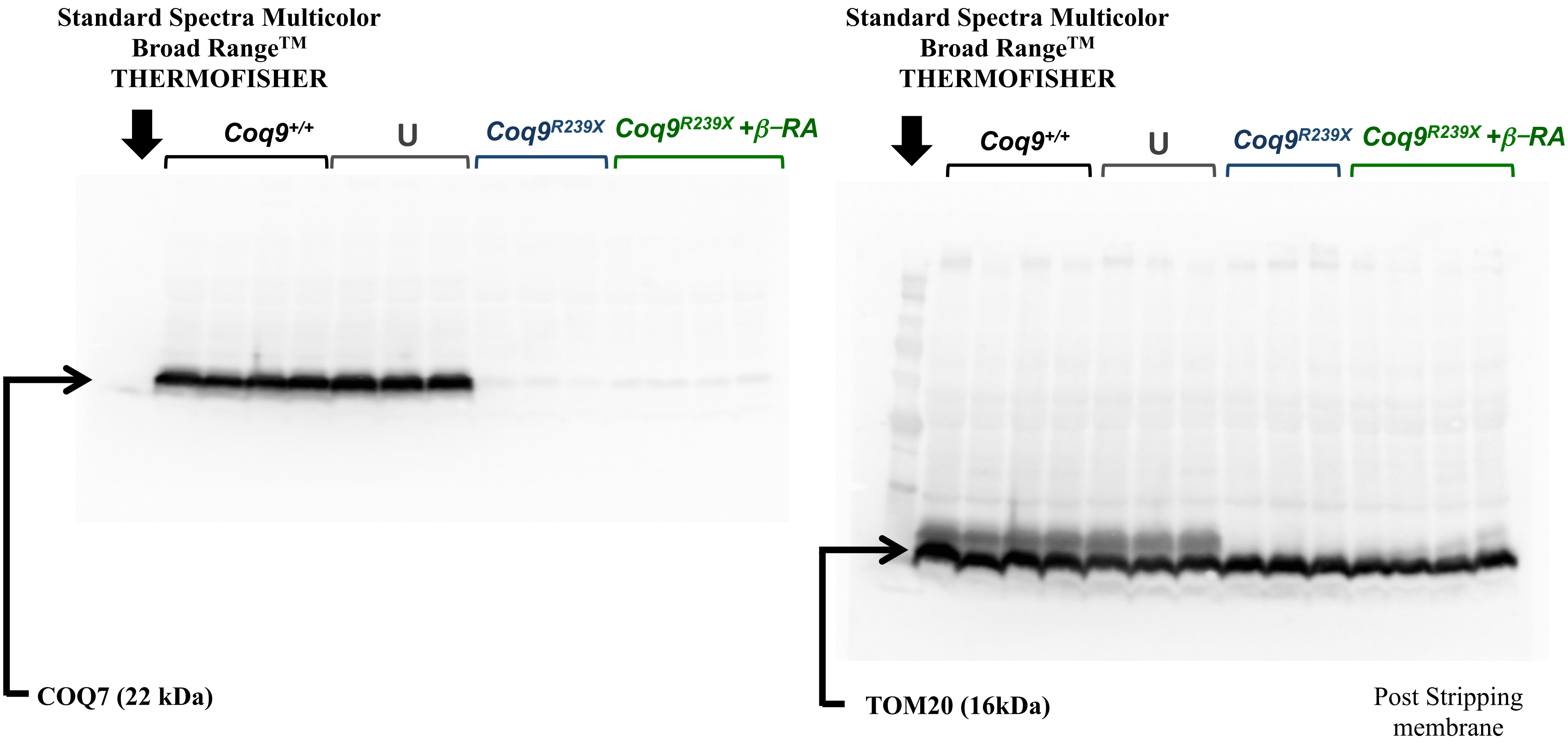

Note: lines 4, 5, 9, 10, 13 and 14 are represented in Figure 6O in the main text.

U=Unrelated to this study

Figure 6O. COQ8A in heart of wild-type and mutant mice with and without treatment.

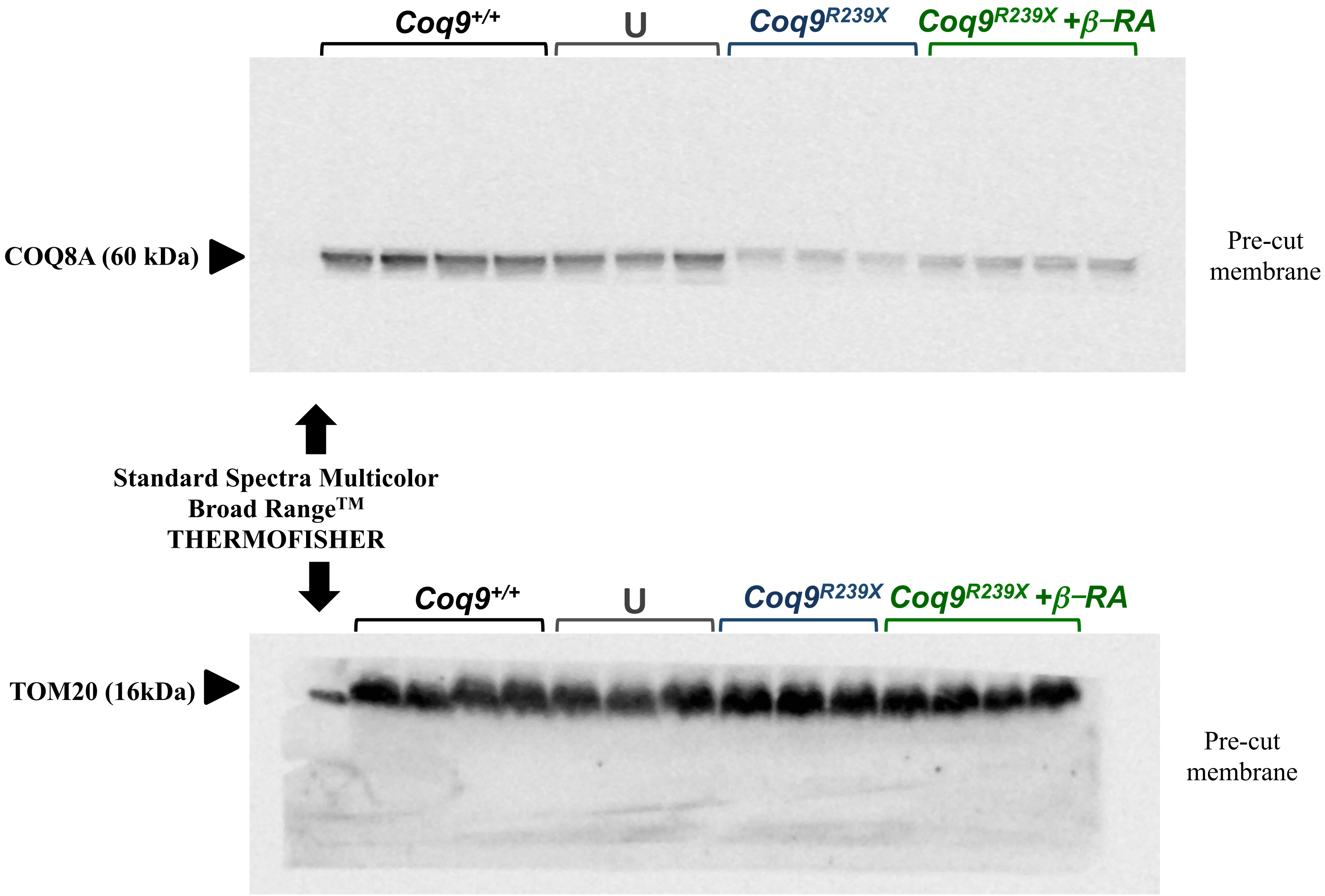

Note: lines 4, 5, 9, 10, 13 and 14 are represented in Figure 6P in the main text.

U=Unrelated to this study
